# The genome-wide identification and transcriptional levels of DNA methyltransferases and demethylases in globe artichoke

**DOI:** 10.1371/journal.pone.0181669

**Published:** 2017-07-26

**Authors:** Silvia Gianoglio, Andrea Moglia, Alberto Acquadro, Cinzia Comino, Ezio Portis

**Affiliations:** Department of Agricultural, Forest and Food Sciences, University of Torino, Grugliasco, Italy; New England Biolabs Inc, UNITED STATES

## Abstract

Changes to the cytosine methylation status of DNA, driven by the activity of C5 methyltransferases (C5-MTases) and demethylases, exert an important influence over development, transposon movement, gene expression and imprinting. Three groups of C5-MTase enzymes have been identified in plants, namely MET (methyltransferase 1), CMT (chromomethyltransferases) and DRM (domains rearranged methyltransferases). Here the repertoire of genes encoding C5-MTase and demethylase by the globe artichoke (*Cynara cardunculus* var. *scolymus*) is described, based on sequence homology, a phylogenetic analysis and a characterization of their functional domains. A total of ten genes encoding C5-MTase (one MET, five CMTs and four DRMs) and five demethylases was identified. An analysis of their predicted product's protein structure suggested an extensive level of conservation has been retained by the C5-MTases. Transcriptional profiling based on quantitative real time PCR revealed a number of differences between the genes encoding maintenance and *de novo* methyltransferases, sometimes in a tissue- or development-dependent manner, which implied a degree of functional specialization.

## Introduction

Alterations to the methylation status of cytosine is a common epigenetic modification used by both prokaryotic and eukaryotic organisms. While in bacteria cytosine methylation is used primarily to defend against invading viral DNA, in eukaryotic species it influences gene expression, transposon movement, gene imprinting and paramutation [1–3]. Typically, a high level of cytosine methylation obtains in and around the centromere, at the telomere and within transposon and pseudogene sequences. Major changes to the pattern of methylation are known to occur during both seed development [4,5] and fruit ripening [6,7]. Despite being a relatively stable mark which can be inherited by daughter cells after cell division, cytosine methylation is still reversible and dynamically regulated [8,9]. The global level of cytosine methylation in a genome depends on the activity of two groups of enzymes, the cytosine-5 methyltransferases (C5-MTases) and a group of DNA glycosylases which act as demethylases. Unlike in animal genomes, where cytosine methylation occurs almost exclusively at CpG dinucleotides, in plants different methylation contexts are known, namely CpG, CpHpG and CpHpH (where H is either A, C or T). In plants, DNA presents the highest methylation rate, probably due to their genome size and complexity and to the great number of transposons [10,11].

Plant C5-MTases have been categorized into three distinct families, namely the METs, which are homologs of the animal DNMT1 family; the chromomethyltransferases (CMTs), a plant-specific group of proteins which feature a chromatin organization modifier and a bromo-adjacent homology (BAH) domain in their N-terminal region; and the domains rearranged methyltransferases (DRMs), which are homologs of the animal DNMT3 family. All C5-MTases share a catalytic domain harboring ten conserved small motifs, but the three families differ at their N-termini, reflecting a degree of functional specialization [12]. In Arabidopsis, the methylation at the symmetric CpG and CpHpG contexts is maintained by MET1 and CMT3, respectively. They replicate the previously established methylation patterns during cell division, using older strands as templates. Indeed, in Arabidopsis *met1-*mutants, a decrease of CG methylation has been showed [13]. Methylation at the asymmetric CpHpH context and *de novo* methylation in all contexts is catalysed by DRM2 and CMT2 that methylate in a manner completely or partially dependent of 24-nt siRNA, respectively [14,15]. Eukaryotic species also harbor the conserved DNMT2 family, which despite having a strong sequence similarity to the C5-MTases and their inclusion of a methyltransferase domain, do not normally show any DNA methyltransferase activity; rather they are thought to be involved in RNA binding and methylation [16–18].

Demethylation can be a passive or an active process. The maintenance methyltransferases do not intervene in the former, so after a number of replication cycles, sites become progressively demethylated. Active demethylation is a more complex process, in which methylcytosine is removed via the enzyme-driven introduction of sequence mismatches, which are then repaired by proof-reading enzymes. In plants, the best known form of active demethylation relies on the activity of DNA glycosylases/lyases, which break the glycosidic bond between a methylated cytosine and the DNA's deoxyribose skeleton; the nucleotide is then removed by endonuclease activity and the site repaired by DNA polymerase and DNA ligase. In *A*. *thaliana*, four proteins take part in this process, namely ROS1, DME, DML2 and DML3 [19–21].

The globe artichoke (*Cynara cardunculus* var. *scolymus*) is a diploid species (2n = 2x = 34) with a genome size of ~1.07 Gbp. It is a perennial crop, cultivated mostly for its immature inflorescence (*capitulum*), which is part of the traditional Mediterranean diet, but is also regarded as a source of pharmaceutically active secondary metabolites and as a potential biofuel and oil crop [22,23]. Its genomic sequence has recently been acquired [24], along with a wealth of transcriptomic and mapping data [25–27]. The biosynthesis of some key secondary compounds (*i*.*e*. phenolic acids and sesquitepenes lactones) was also elucidated [28–31]. However, no information is available on the functional genomics of important protein families involved in epigenetic regulation. Here, the repertoire of genes encoding C5-MTases and demethylases has been identified, by exploiting genome sequence, and their transcriptional profiles evaluated in different vegetative tissues and in the *capitulum* at different developmental stages.

## Materials and methods

### Identification of globe artichoke C5-MTase and demethylase sequences

C5-MTase and demethylase sequences encoded by the genes in *A*. *thaliana* and tomato (*Solanum lycopersicum*) were retrieved from, respectively the TAIR (www.arabidopsis.org/) and the Sol Genomics Network (solgenomics.net/) databases. These sequences (S1 and S2 Files) were used to search for globe artichoke homologs through a Blastp search of the proteome (www.artichokegenome.unito.it); the chosen E-value threshold was 1e^-5^. The corresponding mRNA sequences were also retrieved.

### Phylogenetic analysis

A phylogenetic analysis of the globe artichoke C5-MTases and demethylases was conducted by comparison with the polypeptide sequences of ten C5-MTases and four demethylases from *A*. *thaliana*, seven C5-MTases and three demethylases from tomato [32], five C5-MTases from maize (*Zea mays*) [33], nine C5-MTases from soybean (*Glycine max*), seven C5-MTases from rice (*Oryza sativa*) [34] and two C5-MTases from strawberry (*Fragaria x ananassa*) [35]. The globe artichoke and *A*. *thaliana* DNMT2-like proteins were also included. All sequences used for tree construction are listed in S1 and S2 Files. Multiple sequence alignments were carried out using Clustal Omega online software (www.ebi.ac.uk/tools/msa/clustalo/), and the subsequent phylogenetic analysis using MEGA7 software [36]. Two unrooted phylogenetic trees were generated for both the C5-MTase and the demethylase sequences, based on applying the neighbor-joining (NJ) algorithm in conjunction with either the *p*-distance or the JTT matrix method, and pairwise deletion of gaps for the computation of evolutionary distances. A confidence level was established for each node by performing a bootstrap analysis with 1,000 iterations.

### The structure and chromosomal location of genes encoding C5-MTase and demethylase

The domain structure of the globe artichoke genes encoding C5-MTases and demethylases was established using hmmer software (hmmer.org/) in combination with the Pfam database (pfam.xfam.org/) and the motif prediction tool MEME (meme-suite.org/tools/meme); for the latter a window width of 10–25 residues was set, and the number of motifs was increased from 3 (default) to 17, to guide MEME in the recovery of all the already known domains (>10 motifs). Motifs uncovered by MEME were checked manually and named following the nomenclature system suggested by Pavlopoulou et al. (2007) [12]. Loci were assigned to their chromosomal position based on the globe artichoke genome sequence (www.artichokegenome.unito.it/). The exon/intron graphical structure was inferred using structural information derived from the globe artichoke genome sequence and with the script available at http://wormweb.org/exonintron. The CoGe platform (genomevolution.org) was used to identify paralogs likely generated by a whole genome duplication event [37]. To compute chains of syntenic genes, the DAGchainer module (with the ‘relative gene order’ option activated and the ‘maximum distance between two matches’ parameter set to 20) was used in conjunction with the Quota-Align algorithm (with the maximum distance between two blocks set to 20 genes), both of which are implemented within the CoGe SynMap function. The chromosomal locations of ohnologous methylase/demethylase sequences were visualized using CIRCOS software (circos.ca). The presence and location of nuclear localization signals (NLS) were predicted using cNLS Mapper software (nls-mapper.iab.keio.ac.jp/cgi-bin/NLS_Mapper_form.cgi), with the cut-off score set to 5.

### Protein modeling

RaptorX (raptorx.uchicago.edu/) software was used to generate-likely secondary and tertiary protein structures, which were then visualized using Chimera software (www.cgl.ucsf.edu/chimera/) to allow for structural comparisons to be made. The Chimera MatchMaker tool was used to superimpose the structures of related globe artichoke and *A*. *thaliana* proteins in order to reveal the extent of structural conservation/divergence in their catalytic and regulatory domains. Global alignments were obtained using the Needleman-Wunsch algorithm based on default settings, while alignments restricted to a single domain, in case structures couldn't be superposed globally, were obtained using the Smith-Waterman algorithm based on default settings. Alignments were refined by iterated pruning. Single domains as identified by Prosite (http://prosite.expasy.org/) and hmmer were highlighted on each structure.

### Plant materials

The F_1_ hybrid globe artichoke variety ‘Concerto’ (www.nunhems.com) was grown to the heading stage in the field at Carmagnola (Turin. Italy). Plant tissue was harvested at a range of developmental stages, snap-frozen in liquid nitrogen and stored at -80°C. The tissues sampled were leaves and roots of a year old plant, immature inflorescences both before (stage 1) and after (stage 2) the formation of the stem and at the commercial harvesting stage (stage 3), and the receptacle at stage 3.

### RNA isolation and quantitative real-time PCR (qRT-PCR) analysis

Frozen tissue was ground to powder using the Tissue Lyser II (Qiagen, Hilden, Germania), from which RNA was extracted using an E.Z.N.A.^®^ Plant RNA kit (OMEGA bio-tek, Norcross, USA), following the manufacturer's protocol. The single cDNA strand was synthesized from a 2 μg aliquot of RNA using a High Capacity RNA-to-cDNA kit (Applied Biosystems, Foster City, USA) as directed by the manufacturer. Transcript abundances were quantified by running qRT-PCRs on an iCycler Real-Time PCR Detection System (Bio-Rad Laboratories, Hercules, USA). The relevant primer sequences, designed with Primer3 (http://bioinfo.ut.ee/primer3/), are listed in S1 Table. Each 20 μL reaction was based on GoTaq^®^ qRT-PCR Master Mix (Promega, Madison USA). The amplification protocol comprised an initial denaturation of 95°C/5 min, followed by 40 cycles of 95°C/5s and 60°C/45s. The output was analyzed by iCycler iQ software. Relative transcript abundances were calculated using the 2^-ΔΔCt^ method, based on the abundance of Actin transcript. The data were subjected to a One-Way analysis of variance (ANOVA), and means (of three biological replicates) were separated from one another using Tukey’s HSD (least significant difference) test.

## Results

### Gene identification, structure and nuclear localization

**Fig 1:**
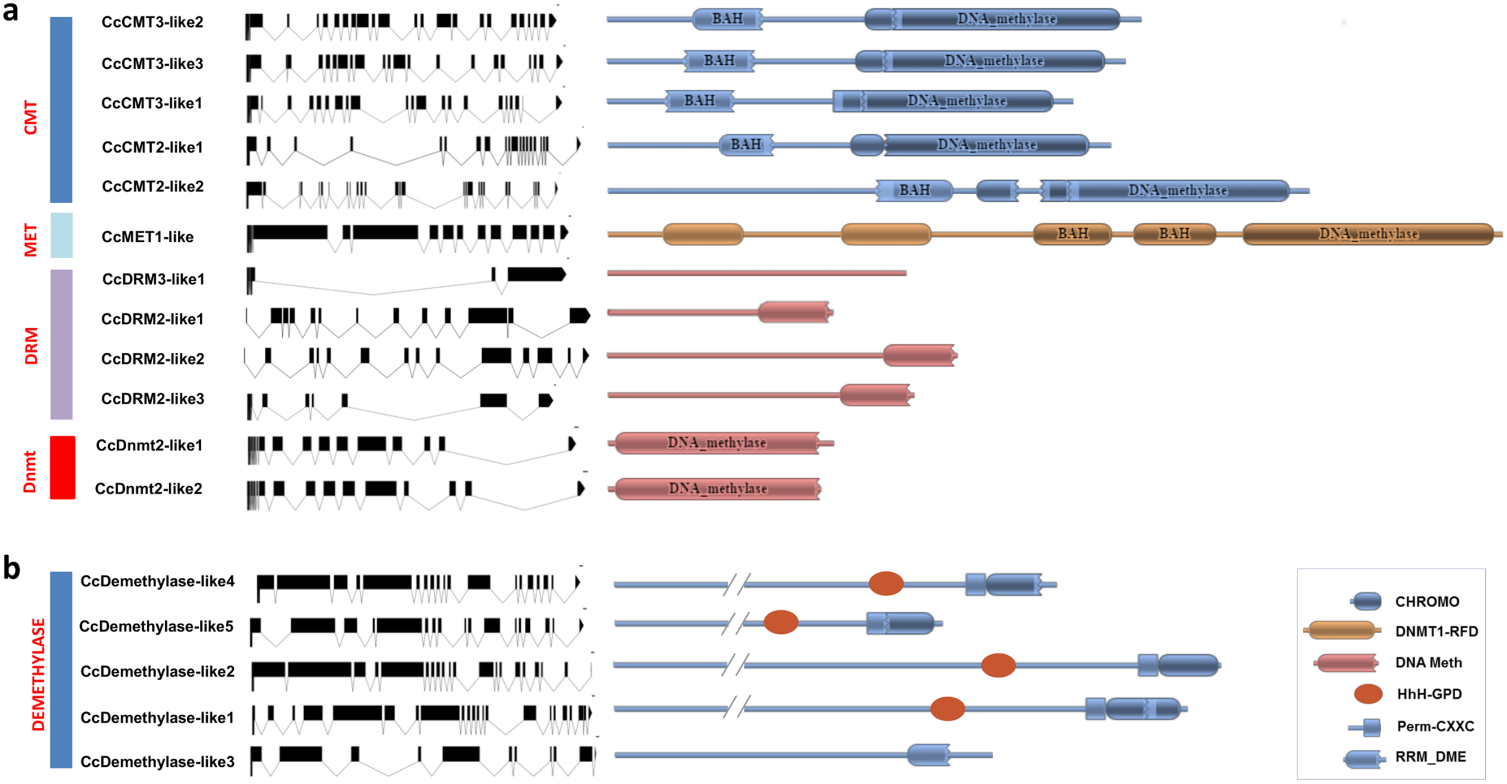
Gene and protein structure of the globe artichoke set of (A) C5-MTases, (B) demethylases. The exon/intron structures are shown in the left hand panel, with exons displayed as black boxes and introns as lines. The graphical representation of gene structure was generated using the tool provided at http://wormweb.org/exonintron. The domains and motifs are shown in the right hand panel, as well as a box explaining the depicted domains (PFAM). The acronym explanations are: DNMT1-RFD = Cytosine specific DNA methyltransferase replication foci domain; DNA meth = C-5 cytosine-specific DNA methylase; CHROMO = Chromo (CHRromatin Organisation MOdifier) domain; HhH-GPD = HhH-GPD superfamily base excision DNA repair protein; Perm-CXXC = Permuted single zf-CXXC unit; RRM_DME = RRM in Demeter.

A total ten globe artichoke genes putatively encoding a C5-MTase, two a DNMT2-like protein (CcDNMT2-like1 and -like2) and five a demethylase were identified (Fig 1). The former were named, on the basis of the identity of their closest homolog, as *CcMet1-like*, *CcCmt2-like1*, *-like2*, *CcCmt3-like1*, *-like2*, -*like3*, *CcDrm2-like1*, *-like2*, *-like3* and *CcDrm3-like1* (Table 1). There were no *Cmt1-like* homologs. The five genes encoding putative demethylases were named CcDemethylase-like1 through 5 (Table 1). The predicted product of the putative C5-MTase genes ranged in size from 364 to 1,535 residues, while the range for the demethylases was 1,405 to 2,029 (Table 1). The 17 loci were each located to a globe artichoke chromosome and their gene models were derived (Table 1, Fig 1). As is also the case for Cmt genes in other plant species, this class of C5-MTase gene has a complex structure, with the coding sequence split into at least 20 exons. DNA demethylases present from 16 to 22 exons.

**Table 1:** The identification of the loci encoding globe artichoke C5-MTases and demethylases and the annotation of the various protein domains.

Locus	Gene name	Chr	Chromosome location	ORFlength (bp)	N° exons	Size (aa)	Domains	Pfam domains
**C5-Methyltransferases**								
Ccrd_v1.0_010519	CcMET1-like	2	57617156..57623706	6,551	12	1535	DNMT1-RFD (2)—BAH (2)—DNA methylase	PF12047-01426-00145
Ccrd_v1.0_007368	CcCMT3-like1	11	4490869..4500026	9,158	20	799	BAH—Chromo—DNA methylase	PF01426–00385–00145
Ccrd_v1.0_002059	CcCMT2-like1	15	11790225..11806714	16,490	20	862	BAH—Chromo—DNA methylase	PF01426–00385–00145
Ccrd_v1.0_001283	CcCMT3-like2	12	37695683..37703077	7,395	22	916	BAH—Chromo—DNA methylase	PF01426–00385–00145
Ccrd_v1.0_006352	CcCMT2-like2	16	5194466..5215990	16,525	27	1203	BAH—Chromo—DNA methylase (2)	PF01426–00385–00145
Ccrd_v1.0_001941	CcCMT3-like3	15	10274366..10283410	9,045	21	889	BAH—Chromo—DNA methylase	PF01426–00385–00145
Ccrd_v1.0_016019	CcDRM2-like1	13	1624979..1627548	2,262	13	753	DNA methylase—UBA	PF00145–00627
Ccrd_v1.0_005163	CcDRM2-like2	7	687910..695172	2,286	14	761	DNA methylase—UBA	PF00145–00627
Ccrd_v1.0_019228	CcDRM2-like3	5	36453314..36463637	10,324	9	524	DNA methylase—UBA	PF00145–00627
Ccrd_v1.0_022006	CcDRM3-like1	10	28887788..28895481	7,694	4	512	DNA methylase—UBA	PF00145–00627
**Demethylases**								
Ccrd_v1.0_014789	CcDemethylase-like1	3	11841625..11853790	12,166	22	1972	HhH-GPD—Perm-CXXC—RRM DME (2)	PF00730—PF15629—PF15628
Ccrd_v1.0_011967	CcDemethylase-like2	1	6701494..6712809	11,316	21	2029	HhH-GPD—Perm-CXXC—RRM DME	PF00730—PF15629—PF15628
Ccrd_v1.0_011203	CcDemethylase-like3	2	68736502..68748601	12,100	11	1405	HhH-GPD—Perm-CXXC—RRM DME	PF00730—PF15629—PF15628
Ccrd_v1.0_007522	CcDemethylase-like4	11	6171240..6180634	9,395	21	1747	HhH-GPD—Perm-CXXC—RRM DME	PF00730—PF15629—PF15628
Ccrd_v1.0_013688	CcDemethylase-like5	1	43768804..43778865	10,062	17	1551	HhH-GPD—Perm-CXXC—RRM DME	PF00730—PF15629—PF15628
**Dnmt2-like genes**								
Ccrd_v1.0_007707	CcDnmt2-like1	11	8713475..8717129	3,655	10	385	DNA methylase	PF00145
Ccrd_v1.0_007710	CcDnmt2-like2	11	8733650..8737296	3,647	10	364	DNA methylase	PF00145

The structurally important domains within the gene products are shown in Table 1 and Figs 1 and S1. All of the putative C5-MTases included the DNA methylation domain PF00145 at their C-terminus. The CMTs harbored both a chromo (chromatin organization modifier) and a BAH (bromo adjacent homology) domain (PF01426), while the single MET1 homolog (CcMET1-like) carried two BAH and two DNMT1-RFD (replication foci, PF012047) domains. CMT2-like2 was the only globe artichoke protein to include two DNA methylation domains (PF00145). UBA (ubiquitin-associated) domains (PF00627) were restricted to the DRM-like proteins. MEME analysis revealed that several conserved motifs were required both for the transfer of a methyl group from S-adenosyl methionine to DNA and for cytosine methylation [20]. Six highly conserved motifs (I, IV, VI, VIII, IX and X, following the notation of Kumar et al. 1994 [38]) were present in all of the C5-MTase and DNMT2-like proteins (S1 Fig). The distribution of these motifs was similar to that noted previously in other plant species [12]. In CcMET1-like, the motifs were ordered I-IV-VI-VIII-IX-X, while in the CMT-like proteins, the chromo domain lay between motifs I and IV, with rest of the arrangement resembling that in CcMET1-like (S1 Fig). Of the five CMTs, four shared a very similar structure, including all of the conserved motifs, while the exception (CcCMT2-like2) was somewhat longer and harbored some non-conserved sequences (S1 Fig). In the DRM-like proteins, the cytosine methyltransferase domain (motifs VI through X) preceded motifs I-IV (S1 Fig). All four CcDRMs harbored a single UBA domain, while DRM3-like1, which has been reported as being catalytically inactive [39], lacked some of the non-conserved domains. DNMT-like proteins conserved the motif order I-IV-VI-VIII-IX-X, but has diverged with respect to the spacing between adjacent motifs (S1 Fig).

The identification of demethylases was somewhat more difficult, because these proteins do not possess well defined, conserved functional domains; nonetheless, all of those identified harbored the two domains Perm-CXXC (permuted single zf-CXXC, PF15629) and HhH-GPD (helix-hairpin-helix Gly/Pro, PF00730) (included in the motif 1 and 2, respectively, S2 Fig), although the copy present in CcDemethylase-like3 was substantially diverged from the canonical HhH-GPD domain sequence (Fig 1). Cc_Demethylase-like1 (Table 1) was the only protein harboring two RRM DME domains (recognition motif demethylase—PF15628, partially included in motif 3, S2 Fig). MEME analysis revealed three major conserved motifs (S2 Fig): two of the demethylases (Cc_Demethylase-like1 and 2) carried all three, Cc_Demethylase-like4 carried motifs 1 and 2, Cc_Demethylase-like5 motifs 2 and 3, and Cc_Demethylase-like3 only motif 2.

Table 2 documents the presence of NLSs in the globe artichoke C5-MTases and demethylases; all included at least one NLS, and most of them displayed both mono- and bipartite NLSs. The presence of multiple NLSs, their position in protein sequences and their "strength" are likely regulating factors of nuclear import and hence protein function. Different NLSs (monopartite or bipartite) can be predicted for a given peptide: those with the highest score are shown.

**Table 2:** The identification of mono- and bipartite NLSs (cut-off score = 5). An NLS with a score >8 is predicted to localize exclusively in the nucleus.

Protein name	Monopartite NLSs	Starting monopartite NLS	Score monopartite NLS	Bipartite NLSs	Starting position of bipartite NLS	Score bipartite NLS
**C5-Methyltransferases**						
CcMET1-like	1	353	6.5	7	35–604–930–1068–1286–1410	12.2–5.9–7–5.7–6.1–5.1
CcCMT3-like1	1	36	7.5	2	166–610	5.9–6.5
CcCMT2-like1	-	-	-	3	52–227–389	6.9–5.2–5.7
CcCMT3-like2	-	-	-	2	10–208	7–5
CcCMT2-like2	1	219	5.5	4	155–216–346–1091	5.3–7.5–5.4–5.1
CcCMT3-like3	-	-	-	2	9–1896	5.5–5.5
CcDRM2-like1	1	8	7	2	89–114	6–5.8
CcDRM2-like2	1	235	11.5	1	223–336	5.5–5.4
CcDRM2-like3	2	157–170	8.5–12.5	2	159–255	19.3–5.1
CcDRM3-like1	-	-	-	1	239	5.1
**Demethylases**						
CcDemethylase-like1	1	926	5	-	-	-
CcDemethylase-like2	1	1484	7	-	-	-
CcDemethylase-like3	4	153–407–477–627	9.5–7–8–8	2	152–171	5.2–10.5
CcDemethylase-like4	2	463–667	6–8	1	244	7
CcDemethylase-like5	-	-	-	1	443	6.6
**Dnmt2-like proteins**						
CcDnmt2-like1	1	286	6	-	-	-
CcDnmt2-like2						

### Syntenic region analysis

**Fig 2:**
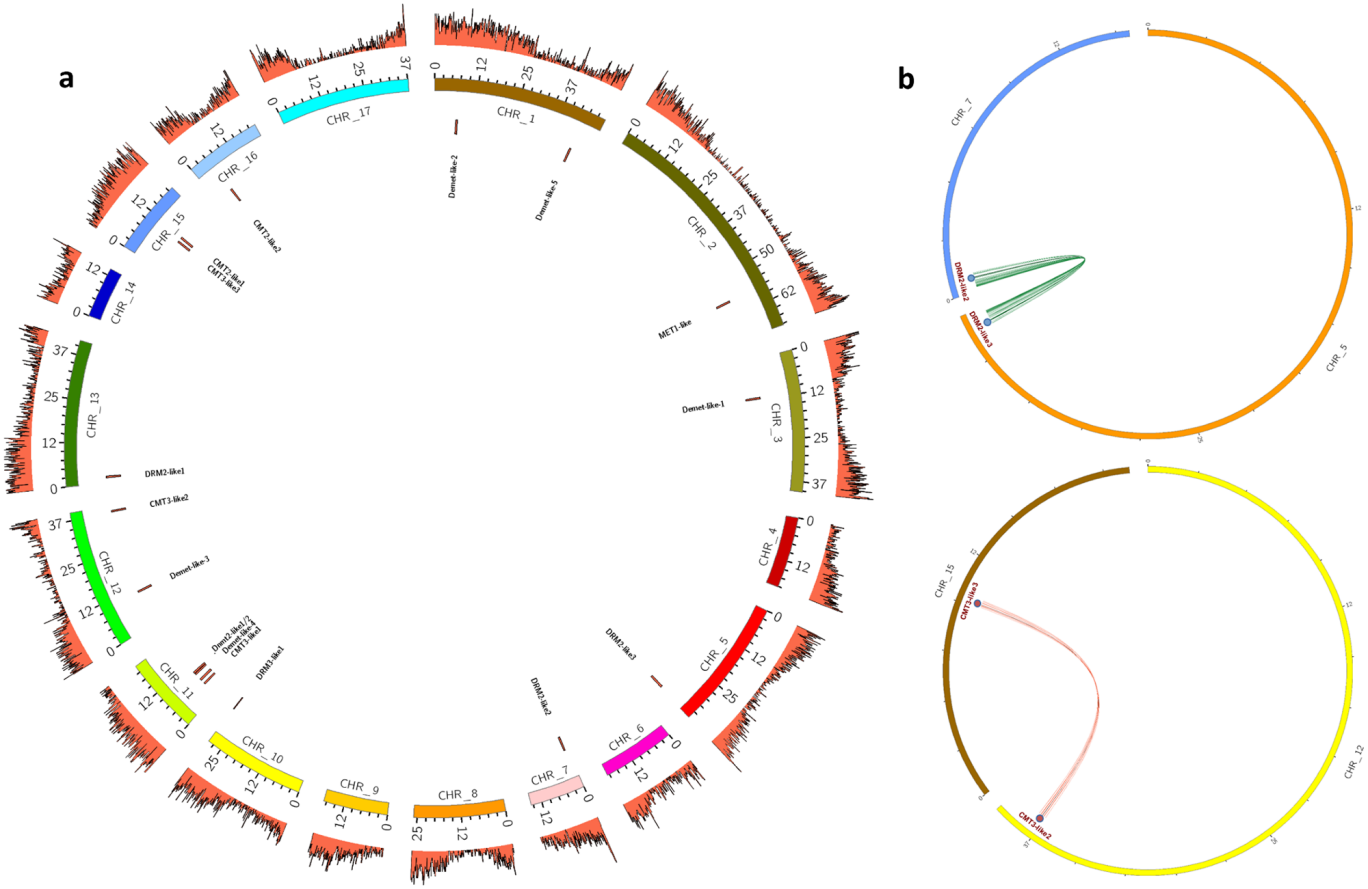
The genomic distribution of genes encoding C5-MTase and demethylase. (A) Chromosomal location: the 17 pseudomolecules (chromosomes) are depicted by the set of colored bars in the center, and gene density (1 Mbp windows) by the outer orange track. The location of each gene is showed by a black tag. (B) Ohnologous gene distribution over the globe artichoke genome (*Drm-like* on chromosomes 7 and 5, *Cmt-like* on chromosomes 12 and 15). Syntenic regions are indicated.

Some C5-MTase and demethylase multiple gene copies were observed in globe artichoke genome (Fig 2A). The pairs CcCMT3-like2, CcCMT3-like3 and CcDRM2-like-2, CcDRM2-like-3 shared the same functional domains and a high level of sequence similarity (~80% and ~70%, respectively), were well separated from one another in the genome, but lay within a syntenic segment; thus they are likely the product of a whole genome duplication event (Fig 2A). CcDRM2-like-2 and CcDRM2-like-3 (on chromosome 7 and chromosome 5, respectively) are possible ohnologs [37], as are CcCMT3-like-2 (chromosome 12) and CcCMT3-like-3 (chromosome 15) (Fig 2B). In contrast, CcDNMT2-like1 was only separated from CcDNMT2-like2 by 16.5 Kbp; these two genes both displayed ten exons and their sequences were 95% identical at the nucleotide level, suggestive of localized duplication event.

### Phylogenetic analysis

**Fig 3:**
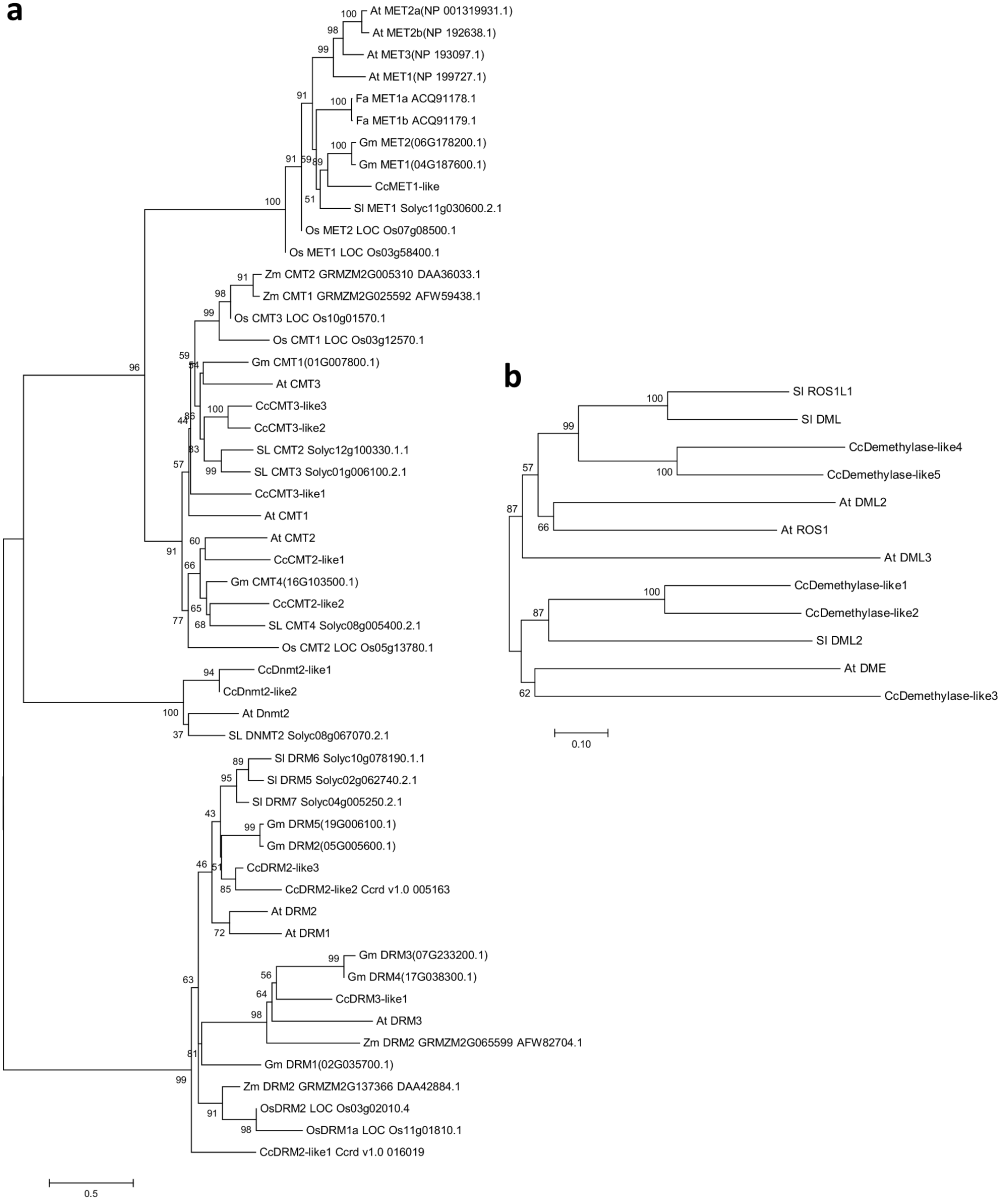
Phylogenetic analysis of the genes encoding (A) C5-MTase, (B) demethylase. An unrooted, neighbour-joining tree was constructed by aligning the protein sequences (given in S1 and S2 Files) present in globe artichoke (Cc), *A*. *thaliana* (At), rice (Os), tomato (Sl), maize (Zm), soybean (Gm) and strawberry (Fa). The number at each node represents the bootstrap value from 1,000 replicates.

The globe artichoke C5-MTase and demethylase polypeptide sequences were aligned with those of their homologs from *A*. *thaliana*, tomato, soybean, strawberry, rice and maize. The resulting p-distance and JTT matrix-based unrooted trees were well correlated with one another, and the latter was presented in Fig 3A. The four clades revealed corresponded to the three plant DNA C5-MTase families (MET, CMT and DRM) and the DNMTs, with bootstrap support values close to 100. The MET and CMT clades showed as quite closely related to one another, with each member of each of the clades harboring a BAH domain. The MET clade formed two sub-clades, both distinct from the rice protein OsMET: one grouped together all of the *A*. *thaliana* METs and the other METs from other dicotyledonous species (including CcMET1-like). The CMT clade also formed two sub-clades, one including only the CMT2s (along with the two CcCMT2-like sequences), and the other the CMT1s and CMT3s (including the three CcCMT3-like sequences). Four sequences, of which two were from globe artichoke, clustered in the DNMT2 clade, while the rest of the sequences belonged to the DRM clade. The latter formed three sub-clades, with a clear separation between the DRM2s (including two of the three CcDRM2-like sequences), the DRM3s (including CcDRM3-like1) and the rice and maize proteins. The sequence CcDRM2-like1 was placed distinctively from all the others of the DRM clade. The equivalent analysis for the demethylases involved five globe artichoke, four *A*. *thaliana* and three tomato sequences (Fig 3B). The sequences fell into two clades, one centered on ROS1 (including CcDemethylase-like4 and -like5) and one on DME (including CcDemethylase-like1, -like2 and -like3), separated with high bootstrap support values.

### Protein modeling

**Fig 4:**
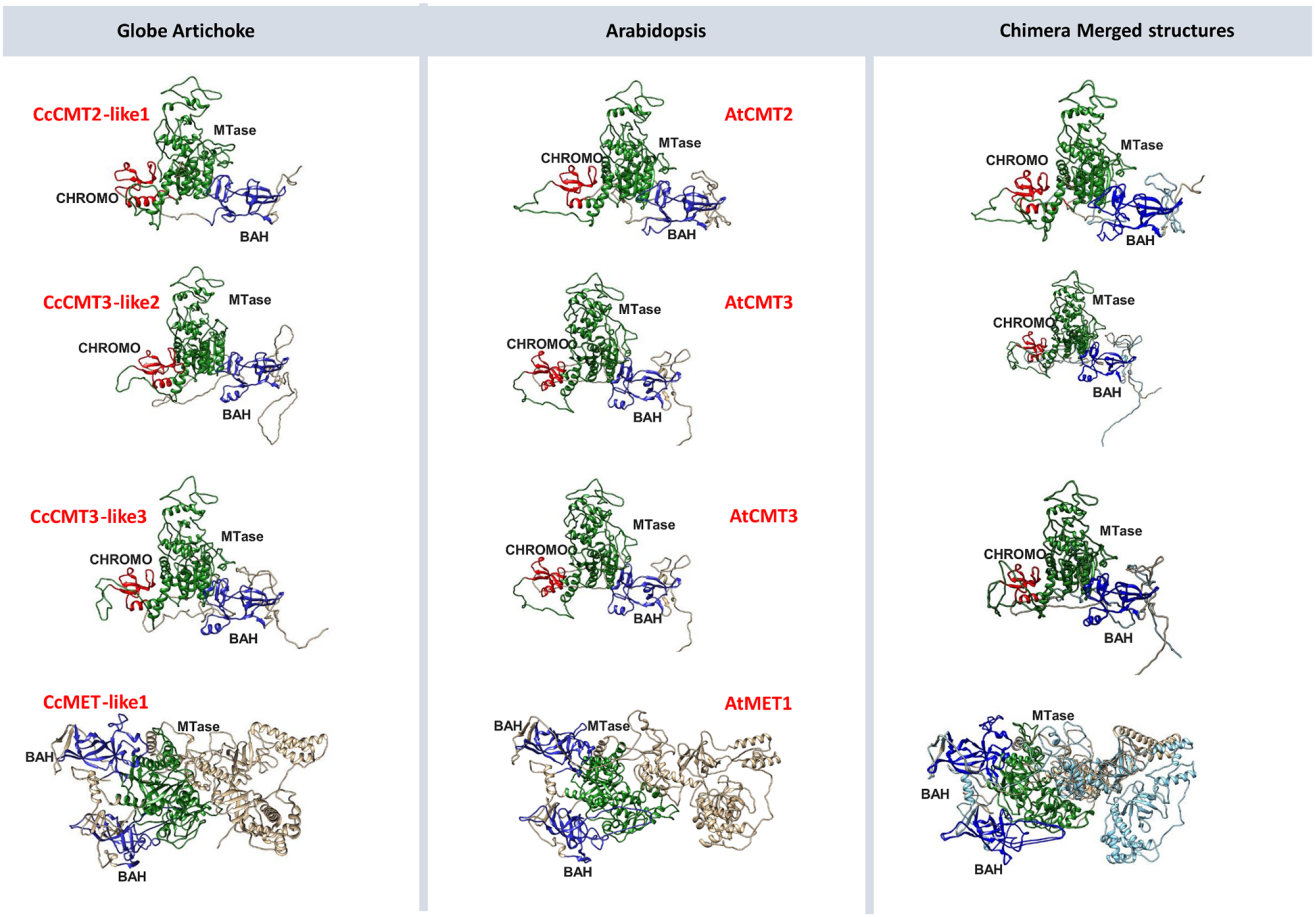
Protein models of the globe artichoke MTases. Their three dimensional structures have been compared to those of their *A*. *thaliana* homologs. MTase domains are highlighted in green, BAH domains in blue and chromo domains in red. The third column represents an overlay of the two structures.

A high degree of conservation was generally obtained with respect to the C5 methyltransferase domain across all the relevant gene families, both within and between species. The BAH and chromo domains were also well conserved. Less can be concluded regarding conservation of the entire sequence, since the analysis performed by the RaptorX program tends to be most reliable for the C-terminal end of the protein and is strongly focused on the catalytic domains. A comparison of the three dimensional structures of the globe artichoke and *A*. *thaliana* METs and CMTs indicated a high degree of structural conservation (Fig 4), which was not the case for the DRMs (S3 Fig). For the latter, it was only possible to consider a localized alignment of the domains to achieve accurate superposition. This analysis suggested that while their C5-methyltransferase domains have been well conserved, structural similarity in their N-terminal portion was limited to the 45 residues making up the UBA domain—which was absent altogether from CcDRM2-like3. Structural conservation in the demethylases, both within globe artichoke and between globe artichoke and *A*. *thaliana*, appeared to be restricted to the three RRM DME, Perm-CXXC and HhH GPD domains (S4 Fig), which are common to all members and are a characteristic of base excision DNA repair proteins. Of the five globe artichoke CcDemethylase-like proteins, three (CcDemethylase-like1, -like2 and -like3) were assigned to the DME family as a result of their alignment with AtDME, while CcDemethylase-like4 and -like5 were assigned to the ROS1 family. The CcDemethylase-like5 HhH GPD domain displayed a particularly high level of structural similarity with AtROS1.

### Transcriptional profiling

**Fig 5:**
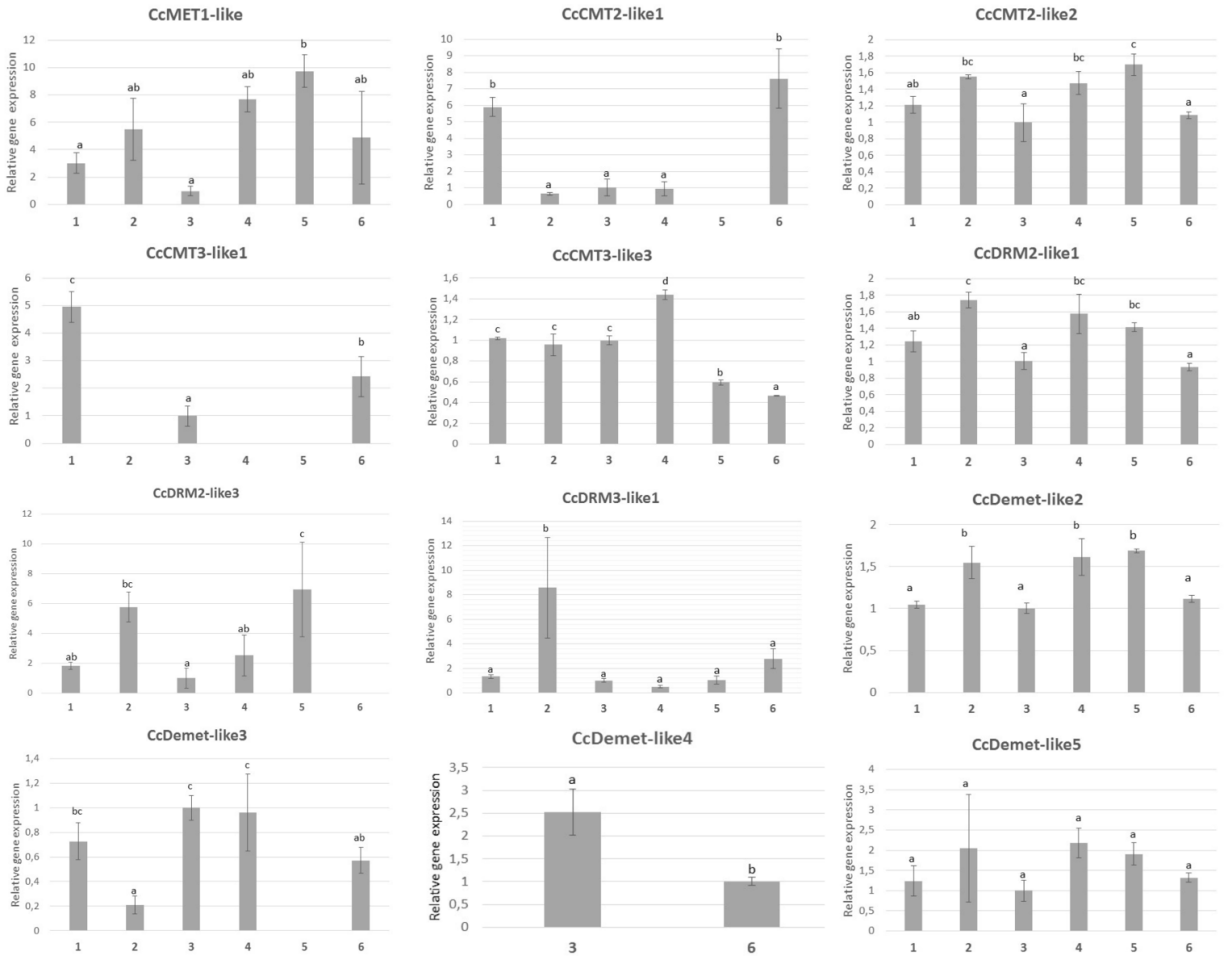
qRT-PCR based transcription profiling of the globe artichoke genes *Met1*, *Cmt2-like*, *Cmt3-like*, *Drm2-like*, *Drm3-like* and *Demethylase-like* in the leaf, root, receptacle and bracts. The globe artichoke gene actin was used as the reference sequence. Numbers on “X” axis represent tissue names:1- leaf; 2-root; 3-receptacle, 4- stage 1 bracts; 5- stage 2 bracts, and 6- stage 3 bracts. Error bars represent SD (*n* = 3, biological replicates). Different letters associated with the set of means indicate a significant difference based on Tukey’s test (P≤0.05) on the basis of the ANOVA analysis.

Transcript abundances were estimated for the various genes in a range of plant organs (mature leaves, roots and receptacle) and in bracts sampled at three different stages of inflorescence development (Fig 5). The analysis excluded genes belonging to the DNMT2 family because their products are thought to be involved in rRNA rather than in DNA methylation [16]. Among the maintenance methyltransferases, some were transcribed at a constant level in all of the assayed material, while others were more organ/developmental stage-specific. *CcMet1* for example was strongly transcribed during the early stages of bract development (stages 1 and 2), with its transcript respectively 7.7 and 9.6 fold more abundant than in the receptacle (Fig 5). The transcription of *CcCmt3-like1* and *CcCmt2-like1* was most noticeable in the leaf and in stage 3 bracts, where its transcript was, respectively, 5.0 and 7.6 fold more abundant than in the receptacle (Fig 5). The transcription of *CcCmt2-like2* was at a similar level across the various organs assayed. The level of *CcCmt3-like3* transcription was similar in the mature leaf, root and receptacle, but strongly decreased during bract development. *CcDrm2-like1* was strongly transcribed in the root and was reduced in the bracts as they developed from stage 1 to stage 3. *CcDrm2-like3* transcription was strongest in the root and in stage 2 bracts, while that of *CcDrm3-like1* was particularly high in the root, reaching an abundance 8.6 fold greater than that in the receptacle. *CcDemethylase-like2* was strongly transcribed in the root and stage 1 and 2 bracts, as was *CcDemethylase-like3* in the receptacle and stage 1 bracts (Fig 5). *CcDemethylase-like4* transcription was confined to the receptacle and leaf, while that of *CcDemethylase-like5* was consistent across all of the organs sampled.

## Discussion

DNA methylation is a common phenomenon in all plant genomes, although its extent varies according to species, ploidy level, genome size and transposon abundance. A positive correlation between cytosine methylation at CpG and CpHpG sites has been established with the content of repetitive DNA elements and consequently also with genome size [40,41]. Within a given genome, the level of DNA methylation is determined by the balance between C5-MTase and demethylase activity. Thanks to the acquisition of its genome sequence, it has been possible here to identify, for the first time in an Asteraceae species, the full repertoire of genes encoding these two classes of enzyme (Fig 1).

In this study we were able to isolate 10 C5-MTases, 5 demethylases and 2 Dnmt2 proteins (Fig 1). The range in size of their predicted products (C5-MTases: 364–1,535 residues, and demethylases: 1,405–2,029 residues) is consistent with those determined for their *A*. *thaliana* and tomato homologs [32].

Each of the globe artichoke C5-MTase genes belonged to one of the three distinct groups MET, CMT or DRM, as is also the case for homologs present in both mono- and dicotyledonous species [12]. Indeed, the domain pattern proves to be conserved across C5 methyltransferase families in *C*. *cardunculus* (S1 Fig) and to reflect that of other plant species, suggesting common evolutionary origin and conserved function. The number of members within each of these groups does vary from species to species: in *A*. *thaliana*, for example, there are four MET group members, while in strawberry, soybean, carrot and rice, the number is only two [12,34,35,42], and—as in globe artichoke—in tomato, tobacco, pea, poplar and peach, the number is just one [12,32,43]. Membership of the CMT and DRM groups is rather higher: respectively, five and four in globe artichoke. Both the *A*. *thaliana* and tomato genomes harbor three members in each group, while the rice genome harbors three CMT and two DRM sequences. The globe artichoke genome lacks a CMT1-like homolog; this gene in *A*. *thaliana* appears to be defective in many ecotypes or practically silent if present [44]. Unlike in *A*. *thaliana*, tomato and rice, where only one DNMT2 gene is present [12,32,42], the globe artichoke genome houses two, highly similar members; their neighboring map locations suggests that they represent the outcome of a localized duplication event. The number and chromosomal distribution of CMT and DRM sequences in globe artichoke (Fig 2B) likely results from the whole genome duplication (WGD) event timed at 40–45 million years ago [24], and shared with most members of the Compositae [45]. WGD events, driving gene family expansion and bursting functional diversification, have created in the course of evolution the potential for novelty and success in many crops [46–48], affecting several pathways (e.g.: glucosinolates, methyltransferases, fruit-controlling genes, resistance genes analogues). In globe artichoke, WGD influenced many pathways [24,49], and in time the duplication of CMT/DRM (and neighbor genes) could have ensured functional redundancy. The conservation of the domain pattern is retained also at the structural level, which can be observed by merging protein models. The methyltransferase domain is remarkably conserved in all members of the different subtypes of C5-MTases. The Chromo domain is involved in chromatin interaction: its architecture is characterized by three beta strands and an alpha helix and is thought to mediate the recognition and binding to target DNA. The BAH domain is also characteristic of chromatin-associated proteins, mediates protein-protein interactions and is involved in gene silencing and duplication; in C5-MTases the BAH domain is found in METs and CMTs. On the other hand, the UBA domain (a 40-aminoacid domain consisting of three helices connected by two conserved loop regions) is found only in DRMs and is generally considered a protein-protein interaction domain. Since domain structure is highly retained but functional specializations, especially regarding methylation context, occur also among the same protein subtype (e.g. CMT2- and CMT3-like proteins), it can be hypothesized that these depend either on punctual differences in aminoacid sequences, or on more divergent regions, especially at the protein N-terminus. There are also cases, like that of CcDRM2-like 3, where the lack of a regulatory domain such as UBA might point to a distinct activity pattern than its close homolog CcDRM2-like 2.

The demethylases belong to the family of DNA glycosylase-lyases, bifunctional glycosylases involved in DNA repair [19]. In all plant species analyzed to date, the number of demethylase genes present is much smaller than that in the other two groups. The globe artichoke demethylases feature three recognizable domains, namely Perm-CXXC, RRM DME and HhH GPD. Perm-CXXC is a permuted version of a single unit of the zf-CXXC domain, a hallmark of the Demeter-like proteins found in land plants; RRM DME is a variant of a 90 residue sequence found in a number of both RNA- and ssDNA-binding proteins; and HhH GPD is associated with the base excision repair DNA glycosylases. Mok et al. (2010) [50], through functional studies in bacterial systems, proved that both these domains are necessary for protein function. Whereas CcDemeth-like1, -like2 and-like3 appear to be related to Demeter-like proteins, CcDemeth-like4 and-like5 lie phylogenetically closer to ROS proteins.

DNA methylation profiles are known to vary during development, for instance during fruit ripening in tomato and sweet orange [32,43,51] and during organ development in rice [42]. Thus it was of interest to track transcription profiles of the various globe artichoke genes encoding C5-MTase and demethylase (Fig 5). MET1 is the major enzyme responsible for the maintenance of cytosine methylation at CpG sites; since CcMET1 was the only member of the MET family to be identified in globe artichoke, the implication is that it maintains CpG cytosine methylation throughout the whole plant. The qRT-PCR assay was able to confirm that the gene was transcribed in all of the tissues samples, most particularly in the root and the mature leaf. The abundance of its transcript declined, however, during bract development. The reduced transcription of DNA methylation-associated genes, as *Met1*, likely might reflect the DNA methylation levels decrease observed in some plant tissues with age [52,53]. The abundance of both *CcCmt2-like2* and *CcCmt3-like3* transcript declined during bract development, while that of *CcCmt3-like1* and *CcCmt2-like1* rose; the suggestion is that these enzymes are associated with a degree of substrate specificity. In *A*. *thaliana*, CMT2 has little impact on the methylation status of CpHpG sites, but a large one on the status of CpHpH sites [14], implying that CMT2s differ from CMT3s with respect to favored target sequence. The similar trend of transcription detected for CcCMT3-like1 and CcCMT2-like1 suggests their involvement in the maintenance of both methylation contexts (i.e. CpHpG and CpHpH, respectively) in the analyzed tissues.

Certain members of the plant DRM family are involved in *de novo* cytosine methylation as a stress response and in the regulation of developmental events such as dormancy. Among all globe artichoke analyzed tissues, *CcDrms* were predominantly expressed in the roots. It is interesting to notice that in the root the low level of transcription of *CcCmt3-like1* and *CcCmt2-like1* is counterbalanced by the high transcription levels of *CcDrms*; this has been previously reported in legumes [34], confirming how few members of different sub-family could express in tissue/developmental stage-specific manner. The dissimilar profiles of *CcDrm2-like1* and *CcDrm3-like1* transcription during bract development are suggestive of their being modulated in a differential manner. Active DNA demethylation in plant genomes is largely carried out by DNA glycosylases belonging to the DME family. Of the five globe artichoke demethylases identified, transcript abundance was measurable for four; for *CcDemethylase-like2*, *-like3* and -*like5*, the level of transcription decreased during bract development, while *CcDemethylase-like4* was transcribed only in the receptacle and the leaf. On the basis of our data we can hypothesize that a reprogramming of DNA methylation patterns occurs during bract developments through: a) the up-regulation of CMT3-like1 and CMT2-like1, responsible for the maintenance of both CHG and CHH context, respectively; b) the down-regulation of DNA demethylases activity.

In conclusion, we report on the identification of 10 C5-MTases and 5 DNA demethylases in globe artichoke. Their genic structure and genomic localization have also been analyzed. Differential transcript abundance of C5-MTase and DNA demethylase genes in different tissues and different developmental stages highlighted their involvement in regulating developmental processes. These information led the way in the C5-MTase and DNA demethylase proteins study in the Asteraceae family that, besides the globe artichoke, includes important food and industrial crops such as *Lactuca sativa*, *Cichorium intybus* and *Helianthus annuus*.

## Supporting information

S1 Fig Conserved motifs in the globe artichoke C5-MTases. (TIF)

S2 Fig Conserved motifs in the globe artichoke demethylases (1, 2, 3). Motif 1 included Perm-CXXC (PF15629) domain, motif 2 included HhH-GPD (PF00730), while motif 3 included part of the RRM DME (PF15628).
(TIF)

S3 Fig Protein models of the globe artichoke DRM-like proteins. Their three dimensional structures have been compared to that of *A*. *thaliana* AtDRM2. MTase domains are highlighted in green and BAH domains in purple.
(TIF)

S4 Fig Protein models of the globe artichoke demethylase-like proteins. Their three dimensional structures have been compared to that of *A*. *thaliana* AtDME. RRM DME domains are highlighted in orange, Perm-CXXC domains in light blue and HhH-GPD domains in magenta.
(TIF)

S1 Table Primer sequences used in the qRT-PCR assays. (DOCX)

S1 File Methyltransferase sequences used for tree construction. *A*. *thaliana* and *S*. *lycopersicum* sequences have also been used for searching for globe artichoke homologs.
(DOCX)

S2 File Demethylase sequences used for tree construction. *A*. *thaliana* and *S*. *lycopersicum* sequences have also been used for searching for globe artichoke homologs.
(DOCX)
