# Supplementary figures and images for: The genome-wide identification and transcriptional levels of DNA methyltransferases and demethylases in globe artichoke
Source: PLoS One. 2017 Jul 26;12(7):e0181669. doi: 10.1371/journal.pone.0181669 (PMC5529103; doi:10.1371/journal.pone.0181669)

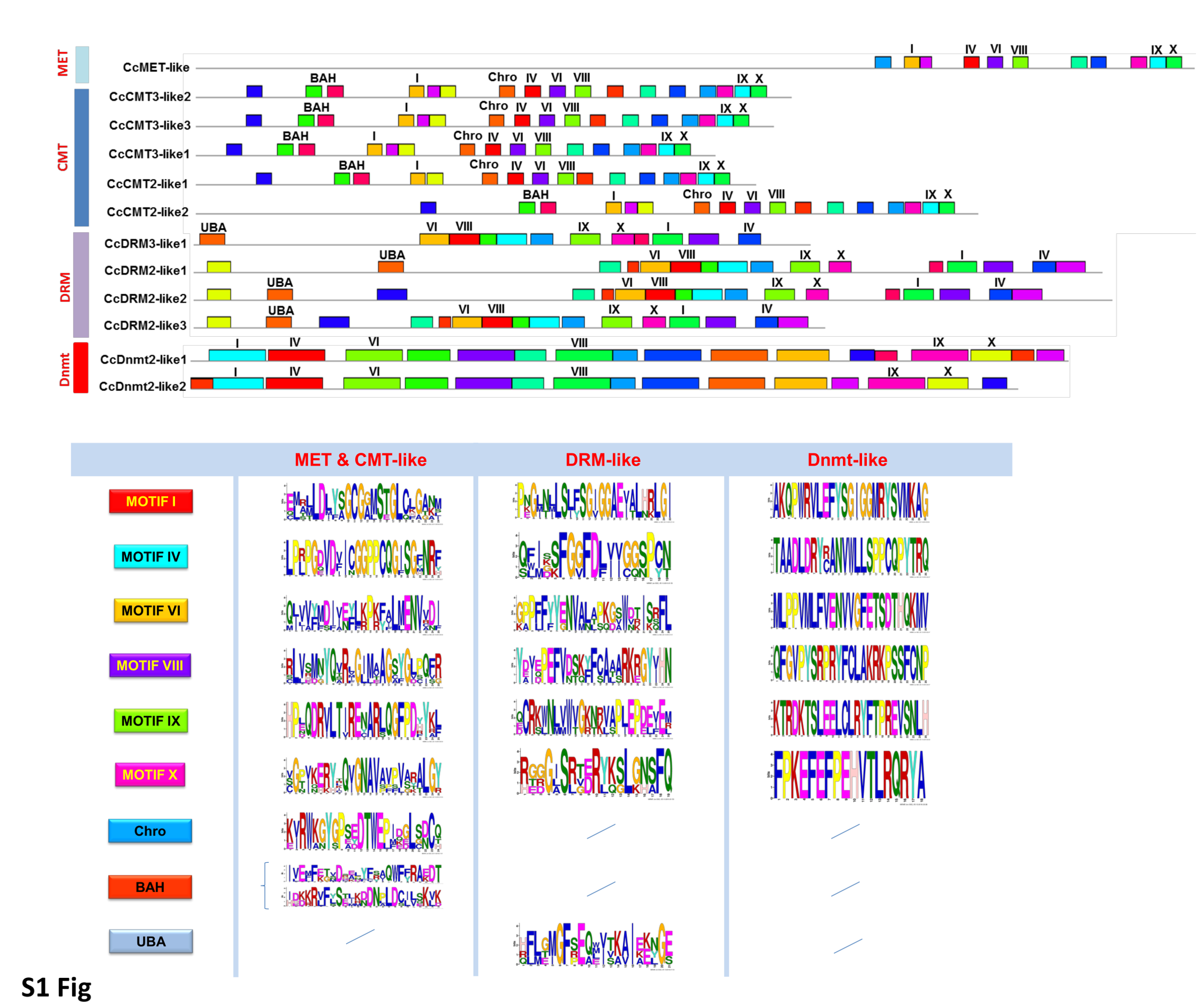

Supplement: S1 Fig — (TIF) [file pone.0181669.s001.tif]

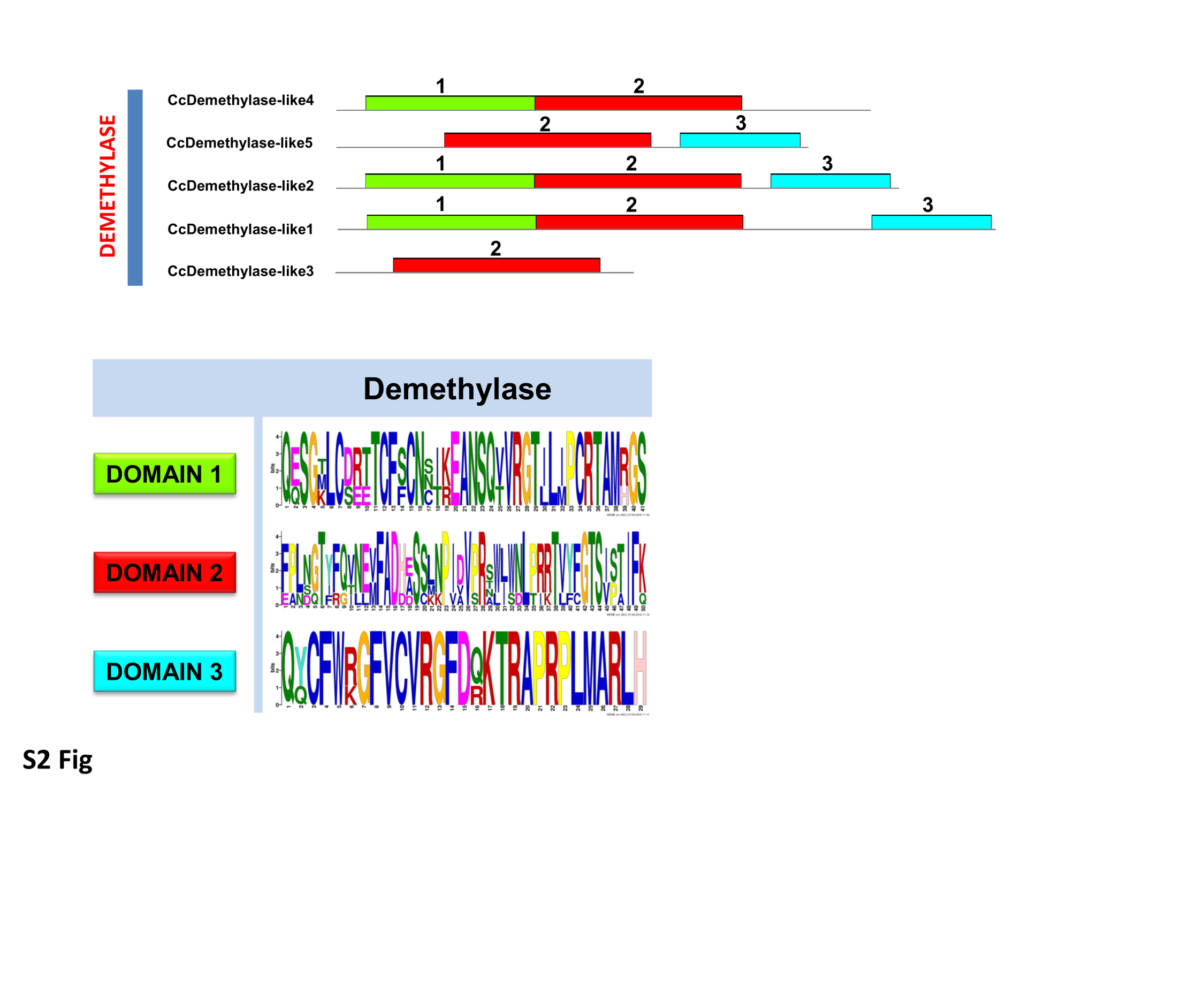

Supplement: S2 Fig — Motif 1 included Perm-CXXC (PF15629) domain, motif 2 included HhH-GPD (PF00730), while motif 3 included part of the RRM DME (PF15628). (TIF) [file pone.0181669.s002.tif]

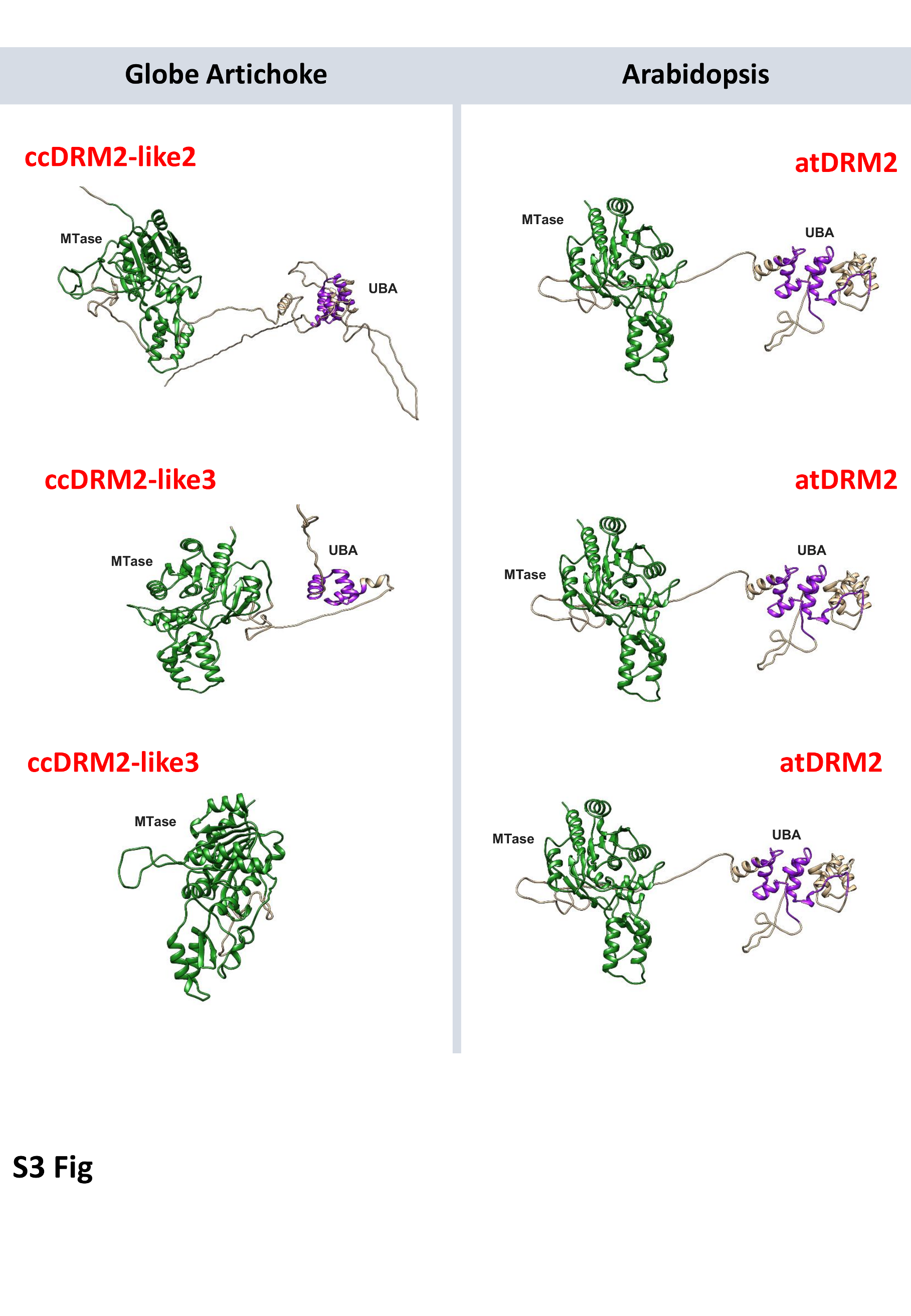

Supplement: S3 Fig — Their three dimensional structures have been compared to that of A. thaliana AtDRM2. MTase domains are highlighted in green and BAH domains in purple. (TIF) [file pone.0181669.s003.tif]

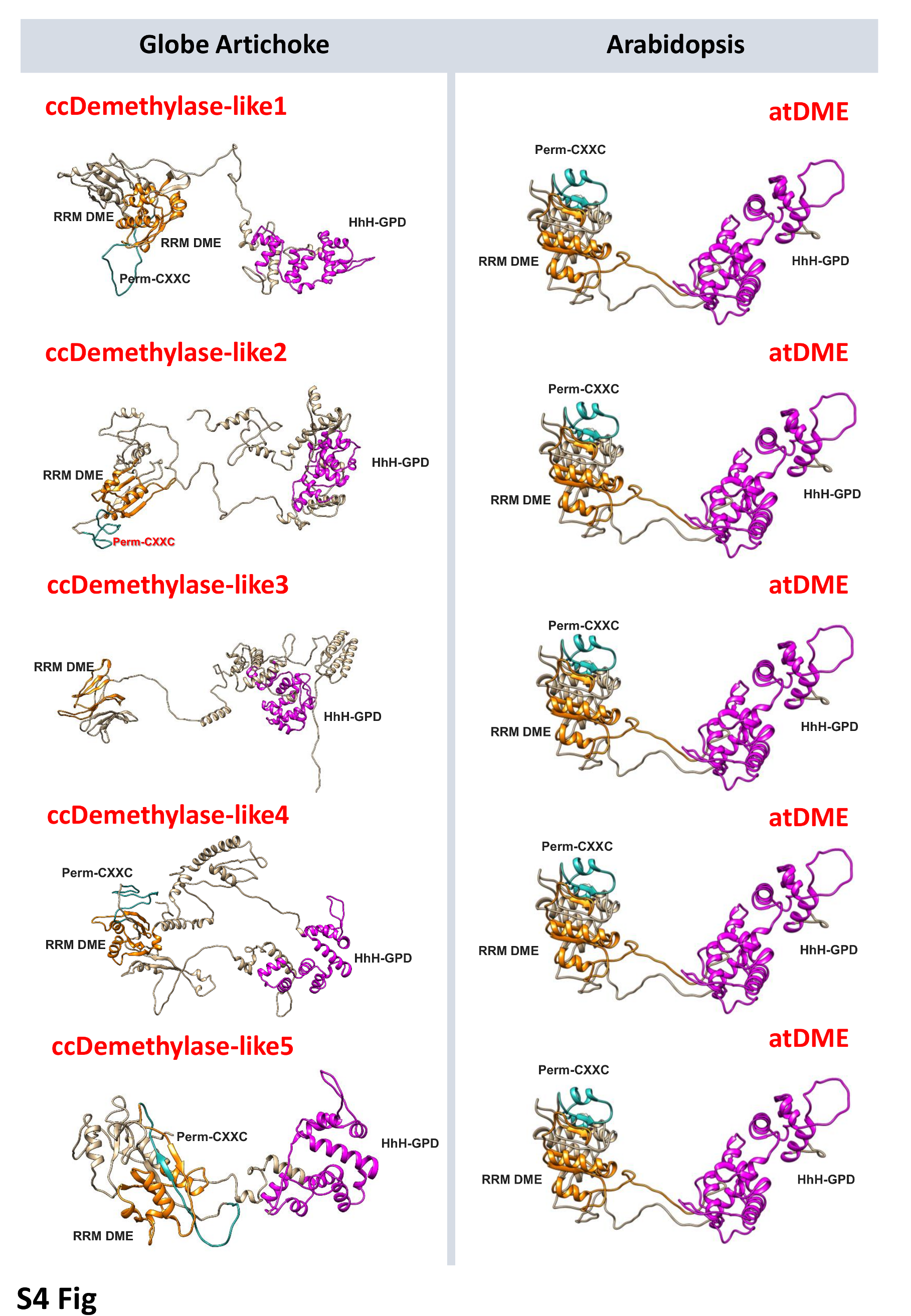

Supplement: S4 Fig — Their three dimensional structures have been compared to that of A. thaliana AtDME. RRM DME domains are highlighted in orange, Perm-CXXC domains in light blue and HhH-GPD domains in magenta. (TIF) [file pone.0181669.s004.tif]
