# Supplementary material for: The genome-wide identification and transcriptional levels of DNA methyltransferases and demethylases in globe artichoke
Source: PLoS One. 2017 Jul 26;12(7):e0181669. doi: 10.1371/journal.pone.0181669 (PMC5529103; doi:10.1371/journal.pone.0181669)
Supplement: S1 Table — (DOCX) [file pone.0181669.s005.docx]

**S1 Table - Primer sequences used in the qRT-PCR assays.**

| **Locus** | **Gene name** | **Left primer** | **Right primer** |
| --- | --- | --- | --- |
| **C5-Methyltransferases** |  |  |  |
| Ccrd_v1.0_010519 | CcMET1-like | GCATTCATTTGGGCAGCCTC | AGTTCTGGACCGGCAAAGAC |
| Ccrd_v1.0_007368 | CcCMT3-like1 | CCCCACGCACAAGTTTTCAG | TTCAGTTCCAGCGGCCTATG |
| Ccrd_v1.0_002059 | CcCMT2-like1 | CCCGGGCATTGTTGTTGATG | TCATCCCCACCATAACCGTGC |
| Ccrd_v1.0_001283 | CcCMT3-like2 | CTGATCTTCCTCCTGTGCCG | TTGACCGGATGCTGAGAACC |
| Ccrd_v1.0_006352 | CcCMT2-like2 | TTGGCTCTGACACTGCACTC | CGATTTTCCATGCGCCAGAC |
| Ccrd_v1.0_001941 | CcCMT3-like3 | ATGCAGCTGTTACCGAGACC | AGCAAAGCCCCGTAGACATC |
| Ccrd_v1.0_016019 | CcDRM2-like1 | CATTCTTCAGGGGTGGTGGG | TCCGCCAAAAGAGTCGATCC |
| Ccrd_v1.0_005163 | CcDRM2-like2 | ATGTCCTGGTGCCTCGATTG | TTTCGTTTCCAGGCCTCGAG |
| Ccrd_v1.0_019228 | CcDRM2-like3 | CGCTTCTTCCTCTTCCTCCG | TCGTTTATCCCATTCCGGCC |
| Ccrd_v1.0_022006 | CcDRM3-like1 | GCAGTTGTGTCAGTTGAGCC | AATCTGCACCAACTCCCCTG |
| **Demethylases** |  |  |  |
| Ccrd_v1.0_014789 | CcDemethylase-like1 | GCACTGAATCCACAAGCTGC | GTAGGGGCTTGGATCATCGG |
| Ccrd_v1.0_011967 | CcDemethylase-like2 | GAATTCCCGTCCCACCTGAG | CCACAGTTCCACTTCCTCCG |
| Ccrd_v1.0_011203 | CcDemethylase-like3 | GGCTGGTTTTGGTGAACGTG | CTTCCTCCTCTCGTGCACAG |
| Ccrd_v1.0_007522 | CcDemethylase-like4 | CTCTTGGAAGGGCTGGACAC | TGTGCCAGATTCTTGGGAGC |
| Ccrd_v1.0_013688 | CcDemethylase-like5 | GGCTGGTTTTGGTGAACGTG | CGCTCTGTTCACTTGCACAC |
|  |  |  |  |
| **Dnmt2-like genes** |  |  |  |
| Ccrd_v1.0_007707 | CcDnmt2-like1 | CATCGGCCATTTCAGGGTAAC | ATACGGTTGGCATGGTGGAG |
| Ccrd_v1.0_007710 | CcDnmt2-like2 | TGCCTCAGATACTTCACGCC | CCACTGCTGCACTCAAACTG |
|  |  |  |  |
| **Actin gene** |  | TACTTTCTACAACGAGCTTC | ACATGATTTGAGTCATCTTC |
|  |  |  |  |
