# Supplementary material for: The genome-wide identification and transcriptional levels of DNA methyltransferases and demethylases in globe artichoke
Source: PLoS One. 2017 Jul 26;12(7):e0181669. doi: 10.1371/journal.pone.0181669 (PMC5529103; doi:10.1371/journal.pone.0181669)
Supplement: S1 File — A. thaliana and S. lycopersicum sequences have also been used for searching for globe artichoke homologs. (DOCX) [file pone.0181669.s006.docx]

**S1 File**

>At_CMT1

MAARNKQKKRAEPESDLCFAGKPMSVVESTIRWPHRYQSKKTKLQAPTKKPANKGGKKEDEEIIKQAKCHFDKALVDGVLINLNDDVYVTGLPGKLKFIAKVIELFEADDGVPYCRFRWYYRPEDTLIERFSHLVQPKRVFLSNDENDNPLTCIWSKVNIAKVPLPKITSRIEQRVIPPCDYYYDMKYEVPYLNFTSADDGSDASSSLSSDSALNCFENLHKDEKFLLDLYSGCGAMSTGFCMGASISGVKLITKWSVDINKFACDSLKLNHPETEVRNEAAEDFLALLKEWKRLCEKFSLVSSTEPVESISELEDEEVEENDDIDEASTGAELEPGEFEVEKFLGIMFGDPQGTGEKTLQLMVRWKGYNSSYDTWEPYSGLGNCKEKLKEYVIDGFKSHLLPLPGTVYTVCGGPPCQGISGYNRYRNNEAPLEDQKNQQLLVFLDIIDFLKPNYVLMENVVDLLRFSKGFLARHAVASFVAMNYQTRLGMMAAGSYGLPQLRNRVFLWAAQPSEKLPPYPLPTHEVAKKFNTPKEFKDLQVGRIQMEFLKLDNALTLADAISDLPPVTNYVANDVMDYNDAAPKTEFENFISLKRSETLLPAFGGDPTRRLFDHQPLVLGDDDLERVSYIPKQKGANYRDMPGVLVHNNKAEINPRFRAKLKSGKNVVPAYAISFIKGKSKKPFGRLWGDEIVNTVVTRAEPHNQCVIHPMQNRVLSVRENARLQGFPDCYKLCGTIKEKYIQVGNAVAVPVGVALGYAFGMASQGLTDDEPVIKLPFKYPECMQAKDQI

>At_CMT2

MLSPAKCESEEAQAPLDLHSSSRSEPECLSLVLWCPNPEEAAPSSTRELIKLPDNGEMSLRRSTTLNCNSPEENGGEGRVSQRKSSRGKSQPLLMLTNGCQLRRSPRFRALHANFDNVCSVPVTKGGVSQRKFSRGKSQPLLTLTNGCQLRRSPRFRAVDGNFDSVCSVPVTGKFGSRKRKSNSALDKKESSDSEGLTFKDIAVIAKSLEMEIISECQYKNNVAEGRSRLQDPAKRKVDSDTLLYSSINSSKQSLGSNKRMRRSQRFMKGTENEGEENLGKSKGKGMSLASCSFRRSTRLSGTVETGNTETLNRRKDCGPALCGAEQVRGTERLVQISKKDHCCEAMKKCEGDGLVSSKQELLVFPSGCIKKTVNGCRDRTLGKPRSSGLNTDDIHTSSLKISKNDTSNGLTMTTALVEQDAMESLLQGKTSACGAADKGKTREMHVNSTVIYLSDSDEPSSIEYLNGDNLTQVESGSALSSGGNEGIVSLDLNNPTKSTKRKGKRVTRTAVQEQNKRSICFFIGEPLSCEEAQERWRWRYELKERKSKSRGQQSEDDEDKIVANVECHYSQAKVDGHTFSLGDFAYIKGEEEETHVGQIVEFFKTTDGESYFRVQWFYRATDTIMERQATNHDKRRLFYSTVMNDNPVDCLISKVTVLQVSPRVGLKPNSIKSDYYFDMEYCVEYSTFQTLRNPKTSENKLECCADVVPTESTESILKKKSFSGELPVLDLYSGCGGMSTGLSLGAKISGVDVVTKWAVDQNTAACKSLKLNHPNTQVRNDAAGDFLQLLKEWDKLCKRYVFNNDQRTDTLRSVNSTKETSGSSSSSDDDSDSEEYEVEKLVDICFGDHDKTGKNGLKFKVHWKGYRSDEDTWELAEELSNCQDAIREFVTSGFKSKILPLPGRVGVICGGPPCQGISGYNRHRNVDSPLNDERNQQIIVFMDIVEYLKPSYVLMENVVDILRMDKGSLGRYALSRLVNMRYQARLGIMTAGCYGLSQFRSRVFMWGAVPNKNLPPFPLPTHDVIVRYGLPLEFERNVVAYAEGQPRKLEKALVLKDAISDLPHVSNDEDREKLPYESLPKTDFQRYIRSTKRDLTGSAIDNCNKRTMLLHDHRPFHINEDDYARVCQIPKRKGANFRDLPGLIVRNNTVCRDPSMEPVILPSGKPLVPGYVFTFQQGKSKRPFARLWWDETVPTVLTVPTCHSQALLHPEQDRVLTIRESARLQGFPDYFQFCGTIKERYCQIGNAVAVSVSRALGYSLGMAFRGLARDEHLIKLPQNFSHSTYPQLQETIPH

>At_CMT3

MAPKRKRPATKDDTTKSIPKPKKRAPKRAKTVKEEPVTVVEEGEKHVARFLDEPIPESEAKSTWPDRYKPIEVQPPKASSRKKTKDDEKVEIIRARCHYRRAIVDERQIYELNDDAYVQSGEGKDPFICKIIEMFEGANGKLYFTARWFYRPSDTVMKEFEILIKKKRVFFSEIQDTNELGLLEKKLNILMIPLNENTKETIPATENCDFFCDMNYFLPYDTFEAIQQETMMAISESSTISSDTDIREGAAAISEIGECSQETEGHKKATLLDLYSGCGAMSTGLCMGAQLSGLNLVTKWAVDMNAHACKSLQHNHPETNVRNMTAEDFLFLLKEWEKLCIHFSLRNSPNSEEYANLHGLNNVEDNEDVSEESENEDDGEVFTVDKIVGISFGVPKKLLKRGLYLKVRWLNYDDSHDTWEPIEGLSNCRGKIEEFVKLGYKSGILPLPGGVDVVCGGPPCQGISGHNRFRNLLDPLEDQKNKQLLVYMNIVEYLKPKFVLMENVVDMLKMAKGYLARFAVGRLLQMNYQVRNGMMAAGAYGLAQFRLRFFLWGALPSEIIPQFPLPTHDLVHRGNIVKEFQGNIVAYDEGHTVKLADKLLLKDVISDLPAVANSEKRDEITYDKDPTTPFQKFIRLRKDEASGSQSKSKSKKHVLYDHHPLNLNINDYERVCQVPKRKGANFRDFPGVIVGPGNVVKLEEGKERVKLESGKTLVPDYALTYVDGKSCKPFGRLWWDEIVPTVVTRAEPHNQVIIHPEQNRVLSIRENARLQGFPDDYKLFGPPKQKYIQVGNAVAVPVAKALGYALGTAFQGLAVGKDPLLTLPEGFAFMKPTLPSELA

>At_DRM1

MVMSHIFLISQIQEVEHGDSDDVNWNTDDDELAIDNFQFSPSPVHISATSPNSIQNRISDETVASFVEMGFSTQMIARAIEETAGANMEPMMILETLFNYSASTEASSSKSKVINHFIAMGFPEEHVIKAMQEHGDEDVGEITNALLTYAEVDKLRESEDMNININDDDDDNLYSLSSDDEEDELNNSSNEDRILQALIKMGYLREDAAIAIERCGEDASMEEVVDFICAAQMARQFDEIYAEPDKKELMNNNKKRRTYTETPRKPNTDQLISLPKEMIGFGVPNHPGLMMHRPVPIPDIARGPPFFYYENVAMTPKGVWAKISSHLYDIVPEFVDSKHFCAAARKRGYIHNLPIQNRFQIQPPQHNTIQEAFPLTKRWWPSWDGRTKLNCLLTCIASSRLTEKIREALERYDGETPLDVQKWVMYECKKWNLVWVGKNKLAPLDADEMEKLLGFPRDHTRGGGISTTDRYKSLGNSFQVDTVAYHLSVLKPLFPNGINVLSLFTGIGGGEVALHRLQIKMNVVVSVEISDANRNILRSFWEQTNQKGILREFKDVQKLDDNTIERLMDEYGGFDLVIGGSPCNNLAGGNRHHRVGLGGEHSSLFFDYCRILEAVRRKARHMRR

>At_DRM2

MVIWNNDDDDFLEIDNFQSSPRSSPIHAMQCRVENLAGVAVTTSSLSSPTETTDLVQMGFSDEVFATLFDMGFPVEMISRAIKETGPNVETSVIIDTISKYSSDCEAGSSKSKAIDHFLAMGFDEEKVVKAIQEHGEDNMEAIANALLSCPEAKKLPAAVEEEDGIDWSSSDDDTNYTDMLNSDDEKDPNSNENGSKIRSLVKMGFSELEASLAVERCGENVDIAELTDFLCAAQMAREFSEFYTEHEEQKPRHNIKKRRFESKGEPRSSVDDEPIRLPNPMIGFGVPNEPGLITHRSLPELARGPPFFYYENVALTPKGVWETISRHLFEIPPEFVDSKYFCVAARKRGYIHNLPINNRFQIQPPPKYTIHDAFPLSKRWWPEWDKRTKLNCILTCTGSAQLTNRIRVALEPYNEEPEPPKHVQRYVIDQCKKWNLVWVGKNKAAPLEPDEMESILGFPKNHTRGGGMSRTERFKSLGNSFQVDTVAYHLSVLKPIFPHGINVLSLFTGIGGGEVALHRLQIKMKLVVSVEISKVNRNILKDFWEQTNQTGELIEFSDIQHLTNDTIEGLMEKYGGFDLVIGGSPCNNLAGGNRVSRVGLEGDQSSLFFEYCRILEVVRARMRGS

>At_DRM3

MADMRRRNGSGGSSNHERNEQILFPKPETLDFDLPCDTSFPQQIGDNAASSSGSNVKSLLIEMGFCPTLVQKAIDENGQDDFELLLEILTKSTETEPPGPSFHGLMEPKPEPDIEYETDRIRIALLTMKFPENLVDFALDRLGKDTPIDEMVDFIVAAQLAEKYAEESEDSLDGAEINEEDEDVTPVTARGPEVPNEQLFETMDKTLRLLEMGFSNDEISMAIEKIGTKGQISVLAESIVTGEFPAECHDDLEDIEKKVSAAAPAVNRTCLSKSWRFVGVGAQKEDGGGGSSSGTANIKPDPGIESFPFPATDNVGETSRGKRPKDEDENAYPEEYTGYDDRGKRLRPEDMGDSSSFMETPWMQDEWKDNTYEFPSVMQPRLSQSLGPKVARRPYFFYGQLGELSPSWWSKISGFLFGIHPEHVDTRLCSALRRTEGYLHNLPTVNRFNTLPNPRLTIQDAMPHMRSWWPQWDIRKHFNSGTCSNMKDATLLCERIGRRIAECKGKPTQQDQTLILRHCHTSNLIWIAPNILSPLEPEHLECIMGYPMNHTNIGGGRLAERLKLFDYCFQTDTLGYHLSVLKSMFPQGLTVLSLFSGIGGAEIALDRLGIHLKGVVSVESCGLSRNILKRWWQTSGQTGELVQIEEIKSLTAKRLETLMQRFGGFDFVICQNPSTPLDLSKEISNSEACEFDYTLFNEFARVTKRVRDMM

>At_MET1(NP_199727.1)

MVENGAKAAKRKKRPLPEIQEVEDVPRTRRPRRAAACTSFKEKSIRVCEKSATIEVKKQQIVEEEFLALRLTALETDVEDRPTRRLNDFVLFDSDGVPQPLEMLEIHDIFVSGAILPSDVCTDKEKEKGVRCTSFGRVEHWSISGYEDGSPVIWISTELADYDCRKPAASYRKVYDYFYEKARASVAVYKKLSKSSGGDPDIGLEELLAAVVRSMSSGSKYFSSGAAIIDFVISQGDFIYNQLAGLDETAKKHESSYVEIPVLVALREKSSKIDKPLQRERNPSNGVRIKEVSQVAESEALTSDQLVDGTDDDRRYAILLQDEENRKSMQQPRKNSSSGSASNMFYIKINEDEIANDYPLPSYYKTSEEETDELILYDASYEVQSEHLPHRMLHNWALYNSDLRFISLELLPMKQCDDIDVNIFGSGVVTDDNGSWISLNDPDSGSQSHDPDGMCIFLSQIKEWMIEFGSDDIISISIRTDVAWYRLGKPSKLYAPWWKPVLKTARVGISILTFLRVESRVARLSFADVTKRLSGLQANDKAYISSDPLAVERYLVVHGQIILQLFAVYPDDNVKRCPFVVGLASKLEDRHHTKWIIKKKKISLKELNLNPRAGMAPVASKRKAMQATTTRLVNRIWGEFYSNYSPEDPLQATAAENGEDEVEEEGGNGEEEVEEEGENGLTEDTVPEPVEVQKPHTPKKIRGSSGKREIKWDGESLGKTSAGEPLYQQALVGGEMVAVGGAVTLEVDDPDEMPAIYFVEYMFESTDHCKMLHGRFLQRGSMTVLGNAANERELFLTNECMTTQLKDIKGVASFEIRSRPWGHQYRKKNITADKLDWARALERKVKDLPTEYYCKSLYSPERGGFFSLPLSDIGRSSGFCTSCKIREDEEKRSTIKLNVSKTGFFINGIEYSVEDFVYVNPDSIGGLKEGSKTSFKSGRNIGLRAYVVCQLLEIVPKESRKADLGSFDVKVRRFYRPEDVSAEKAYASDIQELYFSQDTVVLPPGALEGKCEVRKKSDMPLSREYPISDHIFFCDLFFDTSKGSLKQLPANMKPKFSTIKDDTLLRKKKGKGVESEIESEIVKPVEPPKEIRLATLDIFAGCGGLSHGLKKAGVSDAKWAIEYEEPAGQAFKQNHPESTVFVDNCNVILRAIMEKGGDQDDCVSTTEANELAAKLTEEQKSTLPLPGQVDFINGGPPCQGFSGMNRFNQSSWSKVQCEMILAFLSFADYFRPRYFLLENVRTFVSFNKGQTFQLTLASLLEMGYQVRFGILEAGAYGVSQSRKRAFIWAAAPEEVLPEWPEPMHVFGVPKLKISLSQGLHYAAVRSTALGAPFRPITVRDTIGDLPSVENGDSRTNKEYKEVAVSWFQKEIRGNTIALTDHICKAMNELNLIRCKLIPTRPGADWHDLPKRKVTLSDGRVEEMIPFCLPNTAERHNGWKGLYGRLDWQGNFPTSVTDPQPMGKVGMCFHPEQHRILTVRECARSQGFPDSYEFAGNINHKHRQIGNAVPPPLAFALGRKLKEALHLKKSPQHQP

>At_MET2a(NP_001319931.1)

MEMETKAGKQKKRSVDSDDDVSKERRPKRAAACTNFKEKSLRISDKSETVEAKKEQILAEEIVAIQLTSSLESNDDPRPNRRLTDFVLHDSEGVPQPVEMLELGDIFIEGVVLPLGDEKKEEKGVRFQSFGRVENWNISGYEDGSPVIWISTALADYDCRKPSKKYKKLYDYFFEKACACVEVFKSLSKNPDTSLDELLAAVSRSMSGSKIFSSGGAIQEFVISQGEFIYNQLAGLDETAKNHETCFVENRVLVSLRDHESNKIHKALSNVALRIDESKVVTSDHLVDGAEDEDVKYAKLIQEEEYRKSMERSRNKRSSTTSGGSSRFYIKISEDEIADDYPLPSYYKNTKEETDELVLFEAGYEVDTRDLPCRTLHNWTLYNSDSRMISLEVLPMRPCAEIDVTVFGSGVVAEDDGSGFCLDDSESSTSTQSNDHDGMNIFLSQIKEWMIEFGAEMIFVTLRTDMAWYRLGKPSKQYAPWFGTVMKTVRVGISIFNMLMRESRVAKLSYANVIKRLCGLEENDKAYISSKLLDVERYVVVHGQIILQLFEEYPDKDIKRCPFVTSLASKMQDIHHTKWIIKKKKKILQKGKNLNPRAGIAPVVSRMKAMQATTTRLVNRIWGEFYSIYSPEVPSEAINAENVEEEELEEVEEEDENEEDDPEENELEAVEIQNSPTPKKIKGISEDMEIKWDGEILGKTSAGEPLYGRAFVGGDVVVVGSAVILEVDDQDDTQLICFVEFMFESSNHSKMLHGKLLQRGSETVLGMAANERELFLTNECLTVQLKDIKGTVSLEIRSRLWGHQYRKENIDVDKLDRARAEERKTNGLPTDYYCKSLYSPERGGFFSLPRNDMGLGSGFCSSCKIRENEEERSKTKLNDSKTGFLSNGIEYHNGDFVYVLPNYITKDGLKKGSRRTTLKCGRNVGLKAFVVCQLLDVIVLEESRKASKASFQVKLTRFYRPEDISEEKAYASDIQELYYSQDTYILPPEAIQGKCEVRKKSDMPLCREYPILDHIFFCEVFYDSSTGYLKQFPANMKLKFSTIKDETLLREKKGKGVETGTSSGMLMKPDEVPKEKPLATLDIFAGCGGLSHGLENAGVSTTKWAIEYEEPAGHAFKQNHPEATVFVDNCNVILRAIMEKCGDVDDCVSTVEAAELAAKLDENQKSTLPLPGQVDFINGGPPCQGFSGMNRFSHGSWSKVQCEMILAFLSFADYFRPKYFLLENVKKFVTYNKGRTFQLTMASLLEMGYQVRFGILEAGTYGVSQPRKRVIIWAASPEEVLPEWPEPMHVFDNPGSKISLPRGLRYDAGCNTKFGAPFRSITVRDTIGDLPPVENGESKINKEYGTTPASWFQKKIRGNMSVLTDHICKGLNELNLIRCKKIPKRPGADWRDLPDENVTLSNGLVEKLRPLALSKTAKNHNEWKGLYGRLDWQGNLPISITDPQPMGKVGMCFHPEQDRIITVRECARSQGFPDSYEFSGTTKHKHRQIGNAVPPPLAFALGRKLKEALYLKSSLQHQS

>At_MET2b(NP_192638.1)

METKVGKQKKRSVDSNDDVSKERRPKRAAACRNFKEKPLRISDKSETVEAKKEQNVVEEIVAIQLTSSLESNDDPRPNRRLTDFVLHNSDGVPQPVEMLELGDIFLEGVVLPLGDDKNEEKGVRFQSFGRVENWNISGYEDGSPGIWISTALADYDCRKPASKYKKIYDYFFEKACACVEVFKSLSKNPDTSLDELLAAVARSMSGSKIFSSGGAIQEFVISQGEFIYNQLAGLDETAKNHETCFVENSVLVSLRDHESSKIHKALSNVALRIDESQLVKSDHLVDGAEAEDVRYAKLIQEEEYRISMERSRNKRSSTTSASNKFYIKINEHEIANDYPLPSYYKNTKEETDELLLFEPGYEVDTRDLPCRTLHNWALYNSDSRMISLEVLPMRPCAEIDVTVFGSGVVAEDDGSGFCLDDSESSTSTQSNVHDGMNIFLSQIKEWMIEFGAEMIFVTLRTDMAWYRLGKPSKQYAPWFETVMKTVRVAISIFNMLMRESRVAKLSYANVIKRLCGLEENDKAYISSKLLDVERYVVVHGQIILQLFEEYPDKDIKRCPFVTGLASKMQDIHHTKWIIKRKKKILQKGKNLNPRAGLAHVVTRMKPMQATTTRLVNRIWGEFYSIYSPEVPSEAIHEVEEEEIEEDEEEDENEEDDIEEEAVEVQKSHTPKKSRGNSEDMEIKWNGEILGETSDGEPLYGRALVGGETVAVGSAVILEVDDPDETPAIYFVEFMFESSDQCKMLHGKLLQRGSETVIGTAANERELFLTNECLTVHLKDIKGTVSLDIRSRPWGHQYRKENLVVDKLDRARAEERKANGLPTEYYCKSLYSPERGGFFSLPRNDIGLGSGFCSSCKIKEEEEERSKTKLNISKTGVFSNGIEYYNGDFVYVLPNYITKDGLKKGTSRRTTLKCGRNVGLKAFVVCQLLDVIVLEESRKASNASFQVKLTRFYRPEDISEEKAYASDIQELYYSHDTYILPPEALQGKCEVRKKNDMPLCREYPILDHIFFCEVFYDSSTGYLKQFPANMKLKFSTIKDETLLREKKGKGVETGTSSGILMKPDEVPKEMRLATLDIFAGCGGLSHGLEKAGVSNTKWAIEYEEPAGHAFKQNHPEATVFVDNCNVILRAIMEKCGDVDDCVSTVEAAELVAKLDENQKSTLPLPGQADFISGGPPCQGFSGMNRFSDGSWSKVQCEMILAFLSFADYFRPKYFLLENVKKFVTYNKGRTFQLTMASLLEIGYQVRFGILEAGTYGVSQPRKRVIIWAASPEEVLPEWPEPMHVFDNPGSKISLPRGLHYDTVRNTKFGAPFRSITVRDTIGDLPLVENGESKINKEYRTTPVSWFQKKIRGNMSVLTDHICKGLNELNLIRCKKIPKRPGADWRDLPDENVTLSNGLVEKLRPLALSKTAKNHNEWKGLYGRLDWQGNLPISITDPQPMGKVGMCFHPEQDRIITVRECARSQGFPDSYEFSGTTKHKHRQIGNAVPPPLAFALGRKLKEALYLKSSLQHQS

>At_MET3(NP_193097.1)

MKTKAGKQKKRSVDSDDDVSRERRPKRATSGTNFKEKSLRFSEKYETVEAKKEQIVGDDEKEEKGVRFQSFGRVENWTISGYEDGSPVIWISTVIADYDCRKPSKKYKKLYDYFFEKACACVEVCKNLSTNPDTSLKELLAAVVRSMNGRKIFSSGGVIQEFVISQGEFIYNQLAGLDETSKNHETKFVDNRVLVSLRDESRKIHKAFSNVALRIDESKVLTSDQLMDGGEDEDLKYAKLLQEEEHMKSMDRSRNKRSSTTSAPNKFYIKINEDEIAHDYPLPSYYKNTKDETDELVLFNAGYAVDARNLPCRTLHNWALYNSDLMLISLEFLPMKPCADIDVTYLGQIKEWKIDFGEDMIFVLLRTDMAWYRLGKPSEQYAPWFEPILKTVRIGTSILALLKNETRMAKLSYTDVIKRLCGLEENDQAYISSTFFDVERYVIVHGQIILQFLTECPDEYIKRCPFVTGLASKMQDRHHTKWIIKKKRKMLQKGENLNLRRGKAPKVSKMKAMQATTTRLINRIWGEFYSIYSPEDPLEEIGAEEEFEEVEDVEEEDENEEEDTIQKAIEVQKADTLKKIRGSCKEMEIRWEGEILGETCAGEPLYGQALVGGRKMDVGGAVILEVDDQGETPLIYFVEYMFESSDNSKKLHGKLLQRGSETVLGTAANERELFLTNECLTVQLKDIKGTVSFEIRSRPWGHQYKKEHMAADKLDRARAEERKAKDLPIEYYCKSLYSPEKGGFFSLPRSDMGLGSGFCSSCKIRENEEERSKTKLNDSKTRFLSNGIKYSVGDFVYQIPNYLSKDRGKRRPVFKYGRNVGLRAFVVCQILDIVDLKEPKKGNTTSFEVKVRRFYRPDDVSAEEAYASDIQEVYYSEDTYILPPEAIKGKCEVMKKTDMPLCREYPILDHVYFCDRFYDSSNGCLKKLPYNMMLKFSTIKDDTLLREKKTETGSAMLLKPDEVPKGKRLATLDIFAGCGGLSYGLEKAGVSDTKWAIEYEEPAAQAFKQNHPKTTVFVDNCNVILRISWLRLLINDRAIMEKCGDVDDCISTTEAAELATKLDENQKSTLPLPGQVDFISGGPPCQGFSRLNRFSDGSWSKNQCQMILAFLSFADYFRPKYFLLENVKTFVSFNEGHTFHLTVASLLEMGYQVRFGLLEAGAYGISQPRKRAFIWAAAPNEVLPEWPEPMHVFNNPGFKIPLSQGLHYAAVQSTKFGAPFRSITVRDAIGDLPPIESGESKINKEEMRGSMTVLTDHICKKMNELNLIRCKKIPKTPGADWRDLPDEHVNLSNGIVKNIVPNLLNKAKDHNGYKGLYGRLDWHGNLPTCITNLQPMGLVGMCFHPDQDRIISVRECARSQGFPDSYKFSGNIKDKHRQVGNAVPPPLAFALGRKLKEALHLRNI

>At_Dnmt2

MAEQELQRINEKKPWQVLEFYSGIGGMRYSLMASGIVSEVVEAFEINDSANDVYQHNFKHRPYQGNIQSLTAADLDKYNADAWLLSPPCQPYTRQGLQKHSGDARAFSFLRILELIPHTTKPPQMLFVENVVGFETSDTHMEMIGTLTKLDYVTQEFILSPLQFGVPYSRPRYFCLAKRKPLPFKSQHSNNKLLWSPDPLYGRDDQVEFGKCQAEEGLDKLLEFCKPVEKFLELAAHVDGEPSSVDDSENGSKDCCGQEGDSVPDSVHQYLVPVSLIERWGNAMDIVYPDSKRCCCFTKSYYRYVKGTGSLLATVQPKIKGKESCLKEQRLRYFTPREVANFHSFPEDFEFPKHISLRQRYAMLGNSLSVAVVAPLLRYLFDS

>CcCMT3-like2

MARGKRKSSAANDDASASSSILEKKPKLVEDKNEEISSNVGVVEASAVLDRVSPAKRSGQKLDKPKKEDEEDEDEVESRFIGDPVPDDEARQRWPHRYATINNNDINFAKKKAGKSGSLETLKGMAPQKELMQARRHFSEALVDGRVNFKLGDDGYVQAGEGEDSYICRIVEMFEGMDCALYFSAQWFYRAKDTIIQACSDLIDDKRVFLSEIKDDNPLDCLLEKLKIVRVPLDADIADKQARLADGDYYYDMSYLVPYSTYQNLPPVYGKHLSYILVSERLKFLLYEQPITDNDGDGNESDSTISSESDVNGVVSEVPQVQDGKRSEMRMLDLYSGCGAMSTGLCLGANMGDVNLVTRWAIDLNRYACESLKLNHPETEVRNESAEDFLMLLKEWEKLCQSFSLVGGGDSQQRMNPASIEEDEGEADDDDDDDSDGLDEEVFEVEKVLSICYGDPKEIKKPGLYLKIRWKGYGPEEDTWEPMEGLCDCHDKIKAFVVKGFNSKILPLPGDVDVICGGPPCQGISGFNRFRNKDKPLEDEKNKQLVVYMDIVEYLKPRFALMENVVDIVKFAKGFLGRYALGRLVSMNYQARVGLMVAGSYGLPQFRRRMFMWGARPSEFNLFNTSFDVQKLPQYPLPTHNVVTRGVSPLEFESNTVVHEEGQKVELEKELFLGDAISDLPPVPNDETRDEMPYEEMPKTEFQKFIRLKKDDMPGFSASGQDSSDHLLYDHRPLKLNDDDYQRVCQIPKRKGANFRDLKGVRVRKDNHVEWDPDVERVYLPSGKPLVPDYAMTFVDGRSSKPFGRMWWDETVPTVVTRAEPHNQAILHPLQDRVLTIRENARLQGFPDYYKLVGPIKERYIQVGNAVAVPVARALGYSLAMSCKGSSGAEATFTLPSKFPNIQPVTSPSVDQQNQ

>CcCMT3-like3

MPSSKRKSRASLKDADLASPTSEKRPKSEEEEKGEILPNADAVEAPAVNNGDSSVRSSGRRSENNKEEAELNDDEESRLVGDPIPDDEARQRWPHRYLGKNDAICYLYGTSLTEPQKELIQARRHFTEALVDGCIPFKLGDDGYVQAGEGEESYICRIVELFEGVDGAPYFYAQWFYRAKDTACSNLIDDKRVFLSEIKDDNPLDCLLQKLKIVRVPLDVDVASKRAMLLDGNYYYDMLYLVPYSTYQNLPLVGSHSSNVLKLKTFIGTQQLYLYTDNEADGNESESTISSESDSNAAVTETSKIQEDDKSEMRMLDLYSGCGAMSTGLCLGANMADVNLVTRWAVDLNRYACESLKLNHPETEVRNESAEDFLWLLKEWHKLCQSFSLVGGGDSQLSGDPMIVKEDETEDAVDDDDDNDGLDEEVFEVDKILSICYGDPKGLKKPGLYLKIRWKNYGPAEDTWEPIDGLGDCQEKIKQFVVNGFKSKILPLPGDVDVICGGPPCQGISGFNRYRNKDKPLEDEKNKQLVVYMDIVEYLKPRFALMENVVDIVKFAKGFLGRYALGRLVSMNYQVRIGLMTAGSYGLPQFRMRMFMWGARPSEKLPQYPLPTHNVVARGVSPVEFESNAVVYDEVSGIELEKELFLGDAISDLPPVANDEERDEIPYQEMPKTEFQKFIRLKKEDMPGFSMVGVESSDHPLYDHRPYKLNDDDYLRVCQIPKRKGANFRDLKGVQVRDDNHVEWDPDVERVYLPSGKPLVPDYAMTFVDGRSSKPFGRLWWDEIVPTVVTRAEPHNQAILHPLQDRVLTIRENARLQGFPDYYKLVGPIKERYTQVGNAVAVPVARALGYSLAMSCKGTASEGPVFRLPERFPNIEAVPVAVAVEENQ

>CcCMT2-like1

MVNSAGTGEGSGSCPPSKPPMAPSSSQPSSITANYDSPALHFGQISRRRSPRLLNCSKDGKDSSVDFNKCPKQKKLRTTSPSDSFPSSPNDAPFLIGDPVSDEEARRRWPWRYECKVLSVMEVLIFSRVPDRGDDNEWALLFFLEHEGYLRSDLFRPSVTQTKKEPFKDDDDKLIANVKCHYLQAKVETHVFDLGDCACVKGEEDGHNHVGRILEFFKTNDSKDFFRIQWFFRAEDTDEAASHHKKRLFYSTLRNDNALDCIVSKVDFRSIPACDYYYDMKYNIDYSTYCTIKDDDCCLFSSHNKKEMHSNGSKTNLNGTLTSFKPHKLELSLLDMYSGCGGMSTGLCFGAKLSGVDLSTIRNESAEDFLDLIKEWDKLCKKYMVKEEKTQGNDSTFAGSADDKPSKAKKIVPEDEYEVERLVDICYSDLDGTSKRGLKFKVRWAGYGPSDDTWEPIKELSNCQEKIREFVQKGIKTNKLPRPGDVDIICGGPPCQGISGFNRHRNFESPLEDEKNYQIVVFMDIINFLRPKYILMENVVDILRFANGRLARYAISCLVRDYYQVRLGIMAAGCYGLPQFRLRQLPPFPLPTHDVVFKYGGASGFERNVVAYDEGQFRNLEKPVLLKDAISDLPLVSNSEVRDKMMYRSAPETEFQKYIRATKSDMLGIASGSSSEVEKSVLHDHRPLQLNEDDYLRGANFRELPGIVVDDDNVVSRAPEAELMPSGKHWVPDYAINLHERKSTKPFARLWWDETVSTVICTPNFRCEAVLHPEQDRVLTIRENARLQGFPDFYALCGTVNERYRQVGNAVAVPVGRALGYTLGMAVQKLCGDEPLITLPPEFAHSTTLDLLQASSLATEP

>CcDRM2-like2_Ccrd_v1.0_005163

MGDHAPGDDSENIDWDTEDELEIQNIAPSSCAQLITRDTEAVISNGKASSSAGPSNTKLVEHFLGMGFREQWVTKAIEKNGEGDHESILDTLFAYQRFKCGDYELPAKRVVAPGLVGSHSGLDIQGVQIFGLLMRISVLQALEDPQQGHNSCHPNHLNSPQQQQCVNDDDLSSDYDESLLDDFSESDSWLGSETEDFDSLPEHEKTLKYLVAMGYTEEEASIAMERCELAYISTNPMGPGEQRNLLWPCGTRTGPGASIAVLTDFIGAAQNAKTEPVFFEDEKRKNLGENGKLKKRKLYELEAWKRNKQKGPLTEQDEVIRLPNPMTGFGVPSEPSSMVTHRTLPDDAIGPPFFYYENVALAPKGSWDTISRFLYDVEPEFVDSKYFCAAARKRGYVHNLPINNRFCILPLPPRTISDALPLTKRWWPEWDKRTKLNCLQTVIASAKLTERIRKDLEKYSDYVPLDVQKKVIEDCRKWNLVWVGKNKVAPLEPDEVEMLLGFPRNHTRGGGISRTDRYKSLGNSFQGNPCQIGFGDWLRVFMPSLPKRLDSTSNPGYRWSTHSLGYRSANNPLVDTVAYHLSVLKDIFPKGINMLSLFSGIGGAEVALHRLGILLNNIVSVEISEANRDIVRSWWEQTNQKGNLVHLPDVQQLNGDQLEQFISSFGGFDLVVGGSPCNNLTGSNRVSRDGLQALDDCLVVGYWLNDIESGYLSNAACIAPSKCFAFGTCVGIVLRPNVKVRLITGLDWTGWARVKKTGEEL

>CcCMT2-like2

MFTGKKSEPCDEVSRTNSASKMVVRGSTRMNSDINVAKWGKFSTTGSDALALTLHSLSSDFYYNSDESPVPLKVYDPSLAHGKSVRRSPRFTMGESIITTKGASKNKSVLGERVEGSSSGRSSRLSSSTALIVGTLDRQRSPKSKALPRRRLRRSPRLSPVTSHAENAGSNKLKRLPQKCLRRSPRLSPIPSCPQIEWEGLHTRVKGERTTDGPKLKRIKSCVEELNSNVVTEKCTKKSSARVQHGNGNCEKLAVPALSSAETVKDDFIPNGDTARPCGLEFHVRKKEDSEVKSTSIRTHDKKAASVGNIDFGYNKAPPVRCKTSVSSTKQQSSTKKQRKLKRASFFVGEPVPEDEAQERWHWRYELKFFAVSKIRVTSEMGFEEEVNGVFQRKLGAETDLNKGKTGQRRKGQSWILNAGEEDELHLNVMCHYLQANVDGCIYRLGDCARIKLHGENNNYKDKDEHRTYTSESASKNRARAQLLIHGEGKREHVGRIVEFFKTSDDENYFRVQWFFRAEDTVMKQAAAFHEKKRLFSSTLMNDNLLDCILSKVKITEKAPALGLQSAIQPSEYYCDMEYSVKYSTFRSLATATNSLVARCDLTLPSSLDANNVTITTTPLELSSCEPYKAELALLDLYAGCGGMSTGLCLGAKISGVKLVTRWAIDYHKSACDSFKQNHPETQVRHITAEDFLELLKEWEKLCKLYVLNDTDRGLETSSNRTKKSIIRESSLLDAEVAPGEYEVSSLVDICYGDPSSTGKHGLKFKVRWKGYSPSEDTWELIQDLSDCQGHIKDFVRSGYMSKILPRPGEVDVICGGPPCQGISGYNRFRNTDDPLTDERNQQIIVFFDIVNFLKPKYALMENVADILRYDKASLGRYAISRLVHMNYQSRLGIMAAGSYGLPQFRLRVFLWGALSSETLPQFPLPTHEVIVRYFPSAEFEQNTVAYDEGQPRELKEATVLRDAISDLPAVTSHEDREEMAYDMPPETEFQKYIRLTKDEINGSTLKGVTDWRSSVLTDHRPYKLSEDDFHRVCHVPRRKGANFRDFPGLVVGADNLVRRDPTKEPVLLPSGRPLVPDYVFTFEKGKSKRPFSRIWWDENVPTVVTFPNLHSQRAIHPEQDRVLTIREYARLQGFPDHYRFCGTVKERYCQVGNAVAVFVSKTLGYALGMAFQKLSGDEALMTLPPDFAFQVPPLDQFSAQL

>CcCMT3-like1

MAKRVKRDLKQSEEAIDSSVPSSDSKSSPPSKKSKTSPAAAAADDDARFIGKPVPADQARAKWPHRYESKNKVKVIASSNGELDGKEIIQAKCHYTKAVVDGIAFDLNDDAFVKAEEGKPDFIARIVEMFETVDRELYCSAQWFFRAEDTVIKSQAHLIDKRRVFYSDMKDDNPLDSIVSKVKIVQLSPNVDLAEKEKALSSFDLYYDMKYSMPVTFTTLHTEKSITESDESSVISGDASSDGVVEKSNKKAKSTEVKECVESQMTLLDLYSGCGAMSTGLCHGTNMSGVKLVTKWAVDINKHACESLKLNHTETEVRNEAAEDFLSLLKEWKNLCKEFCLLGSQHAEDTSIKSEESDSQEKEGNPDPSDGEFEVGKLLAVCYGDPNKVNNKKLHFKVFYFPIVVEVRWKGYGPSYDTWEPFDGLSNCTDAMKEFVSRGYQSRILPLPGDVDFICGGPPCQGISGHNRFRNYTDPLKDPKNHQLVVYMDIIEFLKPKFVLMENVCDIVKFADGILGYHAVGRLVSMNYQTRMGIMAAGSYGDCIVGSDNDKSYKLEKSILLGDAISDLPEVTNNNGKDEMEYAGAPRTSFQKYIRMRKQAVAKDASKRKMLYDHRPLELNEDDYARVCQIPKIKGANFRNLPGVMVGKNNKVEWDPSVERVMLPSGKPLVPNYAMTFVRGTSRKPFGRLSMDDIVTTVVGRAEPHNQVLLHPNQDRVLTIRENARLQGFPDHYKLSGPVKERYLQIGNAVSFSVSTALGYTLAKAVQGVCTSKPLTLPIKFPDCLGQSTVKQAPQESE

>CcMET1-like

MKKKGKQVKATTEMSDVTVDLKTKAATKPKQKRGRSGSSEDVPVSRKMPKRAASCTNFKSKPLRLSEKSATIENKKVQVVEEEIAAISLIAGPDDPRPNRRLTDFVFHDADGMPQPVEMLEVDDIFISGIILPLEKASEKEKETGVRCDGFGRIEDWSISGYEDGSPVIWISTELADYDCVKPAASYKKLFGLFYEKAHACVEVYKRLSKSAGGNPELSLDELLAALVRSMSGSKNFPHGASIRDLIISWGGFIFEQLVGLDEAPNGTDQPFVEIPVLAALRDESKKDEECAGMNVPPGGLMNAPLKISDGEKASKSNGPKPAVDEDEDMKLARVLQENENWQALKQKKRQRTSTSSSKLYIQINEDEIANDYPLPAYYKTTAQETDEYIIFDDFDTVDPDQLPRSMLHNWSLYNCDSRLISLELLPMKPCADIDVTVFGSGIMTADDGSGFCLDNEAGSASGSSGVQNEDGIPIYLSAIKEWMIEFGSSMVFISIRTDMAWYRLGKPSKQYAPWYQTVLKTARLAIAIITLLKEQTRASKLSFSEVIKRLYVVVHGQIILQQFAEFPDDTIRRSAFVSGLEDKMEERHHTKWLVKKKAILTKAENMNPRAAMGPVISKRKAMPATTTQLINRIWGEFYSNYSPEEVKEGDSLDAKKDEEEEEQEENEQEDCEEPEEENPILPQEPEKPSPASKQKKTRCSKTDINWVGQAVRKMCDGKALYKEAVIRGEVVALGSSVLVETSGSEEDSIYYVEYLFEDSDSRKFVHGRLMLRGRQTVLGDIANEREVFLTNDCMEFELDDVIQTVVVQIRTLPWGFEHRKANANFDKMDRAKAEDRKNKGLPIEFYCKSLYWPQRGAFFCLQTDKMGLGNGVCHSCEFMEAEKKKEAFVLDESKTGFTYMATEYHVDDFLYVGPHHFDTDERGNETYKGGRNVGLKAYVVCQLQQIEAPKTSKRADPDSVMVQVRRFYRPEDLSSDKAYRSDIQEVYYSEQVHKLPLSAIEGKCEVRRKKDLSSLDSTYIFEHVFFCERLYDPAKGSLKQLPVHIKLTPPKESLVSDAAIRKRKGKSKEGENDVDMIDNQESSASKNCLATLDIFAGCGGLSEGLQKAGASVTKWAIEYEEPAGDAFKLNHPDALAFVHNCNVILRAIMTACGDTDDCISTSEADELAAKLEEDVINNLPRPGQVDFINGGPPCQGFSGMNRFNQSTWSKVQCEMILAFLSFAEYFRPKFFLLENVRNFVSFNKGQTFRLALASLLEMGYQVRFGILEAGAFGVSQSRKRAFIWAASPEEVLPEWPEPMHVFAGPELKVTLNRNTQYAAARSTATGAPFRAITVRDTIGDLPAVGNGASAATIEYKNESVSWFQKRIRGDASVLTDHISKEMNELNVIRCQRIPKRPGADWRDLPEEKVKLSTGQMVDLIPWCLPNTAKRHNQWKGLFGRLDWEGNFPTSITDPQPMGKVGMCFHPDQDRILTVRECARSQGFPDRYKFSGNIQHKHKQIGNAVPPPLAYALGRKLKEAVEAKQRQLDHHSCL

>CcDRM2-like1_Ccrd_v1.0_016019

MDGDSSHEGSDNIDWNTDDELEIANISPSSPVSTSTNGAVISEFGEVVHNCTQIIADYPTPGMIVIYEPLISDGSKRISWSLCYKAFGFGISIGEVAPRDVIGWTHDLRRYFRGYFVISCLTLVVIAKAPFKPRSILQIPPTMSSSSKCPSKLQFMTMGFPEAMVTKVIAELGEDNTDAIVDTLLTYSIDSLGENEMDVVVDETPNQERELNDPYLKSESHPESKFSDLDSIWSDEDSDESFEKDDPLVCLIDMGYSPEEASAAISRCGKNAPLSELVDFISAANVSKEYDAEMNANVLGSCSHLLPKDKKRKFQKDNFWSKNKKFDRKNESKSKDEDHEVLHLPNPMVGFGVPREPFHVVRRTLPEAAIGPPFFYYENVALTPKGAWNRIKSFLYEIDPEFVDSKFFCAAARKRGYIHNLPLENRFPIQPLPPLTIFEALPGTKKWWPSWDKREQLNCILTCIGSAQLTDRIRLALENSNTEPSLHVKNYIIGQCRKWNMVWTGKTRVAPLEPDEIELIMGYPIYHTRGASRVERYKGLGNAFQVGTLKPPTFYFFNRYYHLKAGLHIFQAKCLIGIQHLVIYILHLIFYTFGGKTSRVFQQEFHILEVDTVAYHLSVLKNLYPNGMNILSLFSGIGGAEIALHKLGIPLNNVVSVEKSMVCRNILQGWWEQTNQKGNLVHLSDVQDVTLNKLNQWIDSFGGFDLVIGGSPCNNLAGGNRRTRDGLEGGHSSLFFDYFRILDGVKNLMKNRS

>CcDRM2-like3

MDGNASGEDYENIDWDTEDELEIQNIVPSTCSNLVTRNASIVGNGEASSSAGPSNPNLLQHFLGMGFSEQLIVKAIKENGEANTESILESLLTYAALEDSPDELNPCHLNSPQQQQCVDNGQLSSDYDESFLDDISESDSWSGSEVAKPKISIGANGGLKKRKLYELELWKRKKRKGLINEEDDVLRLPNPMIGFGVPTDTMVVTHRTLPEAAIGPPFFYYENVALAPKGVWDTISRFLYDVQPEFVDSKYFCATARKRGYVHNLPIHNRFPLLPLPPRTINDALPLTKRWWPEWDKRTKLNCLQTVIGSAKLTDRIRKALEKWGDDPPLHVQKYVIEECRKWNLVWVGKNKLAPLEPDEFEMLLGFPRNHTRGGGISRTDRYKSLGNSFQDMFPNGLNLLSLFSGIGGAEVALHRLGIPLKNVVSVEISEANRDIVRSWWEQTNQKGNLIHLADVQQLNGDRLEQFMGSFGGFDLIVGGSPCNNLAGSNRVSRDGLEGEQSSLFYDYFRILDLVKCIMNKQQ

>CcDRM3-like1

MDKTLRLLEIGFSEQEISAAIEKYGSEVSISELADSIVCDRMGGPCIKTEEDPFGANSWMTGNKFKSSSMGAERVLDASFYSNLALRTEESSQAAASQIRDFDIGDSCKGKQPKEETADELITIQRPKPEFDDLNSYSGPACTVPKPPVSSKVLQRQLKYKARRMAATGVPKLIQPVSCSSVDQMVAKAPLFFYGNVMNLSQDSWVKISQFLYAIEPEFVNTQFFSALSRKEGYIHNLPTKNRFHILPKPPMTIEEVIPQTKKYWPSWDTRKQLTCINSETIGISQLCDRLRNILIDSKGLLSVEQQKDLLHQCRSLNLMWVGRNRVSPIEPELVERILGYPMYHTREDGLSLGERLQSLKHSFQTDTLGYHLSVLKSMYPEGLTLLSIYSGVGGAEITLNRLGIRLKAVVSVEPSEIKRKILRQWWDKSDQTGELVQIENIQKLSSSKLESLIKKFGVFDFIICQNPYTYAPKSVTMAAAETESFAGLDFSLFYEFVRVLQRVRSAIKTR

>CcDnmt2-like1

MEEEGGLPAKQPWRVLEFYSGIGGMRYSVMKAGLQTEMVEAFDINDLANDVYEHNFGHRPFQGNIQTLTAADLDRYCANVWLLSPPCQPYTRQGLQKQSADARASSFLRILEIIPQLMLPPVMLFVENVVGFETSDTHQKMVQILEESQFEMQEFILSPLQFGVPYSRPRYFCLAKRKPSSFCNPEFNRQLLHVPELLVGCNESISISENGQLTSTSDKMLQTCLPIERFLEFGNSMNQVENGTSFPPDTCSDESSLKQYFVPSNLIERWGSAMGILDIKFGIDIVYPDSKRCCCFTKSYYRYVKGTGSLLTTVMPKTRDKTSLEELCLRYFTPREVSNLHSFPKEFEFPEHVTLRQRYALPEKCTEFGVRIVSHMYEIINTHGI

>CcDnmt2-like2

MEEEGLPAKQPWRVLEFYSGIGGMRYSVMKAGLQTEMVEAFDINDLANDVYEHNFGHRPFQGNIQTLTAADLDRYRANVWLLSPPCQPYTRQGLQKQSADARASSFLRILEIIPQLMLPPVMLFVENVVGFETSDTHQKMVQILEESQFEMQEFILSPLQFGVPYSRPRYFCLAKRKPSSFCNPEFNGQLLHVPELLFGCNESISISENGQLTSTSDKMXQTCLPIERFLEFGNSMNQVENGTSFPPNTFSDENSLKQYFVPSNLIERWGSAMGILDIKLFISSILIQSAAVASQRPKTRDKTSLEELCLRYFTPREVSNLHSFPKEFEFPEHVTLRQRYALLGNSLSAAVVAPLLHYLFSEP

>Gm_CMT1(01G007800.1)
MPSKRKTRSSASPAAAPPSKRASRSSASRVADSAPVKSEAEEVVAASSVVKEEAQASFTDVTDGNVSDGEGTNARFVGEPVPDEEARRRWPKRYQEKEKKQSAGPKSNRNDEDEEIQQARRHYTQAEVDGCMLYKLYDDAHVKAEEGEDNYICKIVEIFEAIDGALYFTAQWYYRAKDTVIKKLAYLIEPKRVFFSEVQDDNPLDCLVEKLNIARITLNVDLEAKKETIPPCDYYCDTQYLLPYSTFVNLPSENGESGSETSSTISSETNGIGKYEVNSQPKEAFLPEESKDPEMKLLDLYCGCGAMSTGLCLGGNLSGVNLVTRWAVDLNQHACECLKLNHPETEVRNESAENFLSLLKEWQELCSYFSLVEKKVSHEKYVNLFSEDDDDTSSNEEVNSEDDNELNEDDEIFEVSEILAVCYGDPNKKKEQGLYFKVHWKGYESALDSWEPIEGLSNCKEKIKEFVSRGFKSQILPLPGDVDVICGGPPCQGISGFNRFRNKESPLDDEKNKQLVVFMDIVQYLKPKFTLMENVVDLVKFAEGFLGRYALGRLLQMNYQARLGIMAAGAYGLPQFRLRVFLWGAAPSQKLPQFPLPTHDVIVRGVIPLEFEINTVAYNEGQKVQLQKKLLLEDAISDLPRVQNNERRDEIKYDKAAQTEFQRFIRLSKHEMLELQSRTKSSKSLLYDHRPLELNADDYQRVCRIPKKKGGCFRDLPGVRVGADNKVEWDPDVERVYLDSGKPLVPDYAMTFVNGTSSKPFARLWWDETVPTVVTRAEPHNQAILHPEQDRVLTIRENARLQGFPDFYKLCGPVKERYIQVGNAVAVPVARALGYTLGLAFEGSTSTSDDPLYKLPDKFPMIRDRVSSVSSEDDV*

>Gm_CMT4(16G103500.1)
MNDNLIDCIMGKANVTHITPRVGLKLASISSSDFYYDMEYCVDYSTFRNIPTDASTVTESQPCSELNKTELALLDLYSGCGGMSTGLCLGAKTASVNLVTRWAVDSDRSAGESLKLNHSDTHVRNESAEDFLELLKAWEKLCKRYNVSSTERKLPFRSNSSGAKKRGNSEVHEISDGELEVSKLVDICFGDPNETGKRGLYLKVHWKGYSASEDTWEPIKSLSKCKESMQDFVRKGMKSNILPLPGEVDVICGGPPCQGISGYNRFRNCASPLDDERNRQIVIFMDMVKFLKPRYVLMENVVDILRFDKGSLGRYALSRLVHMNYQARLGIIAAGCYGLPQFRLRVFLWGAHPSEVIPQFPLPTHDVIVRYWPPPEFERNVVAYDEEQPRELEKATVIQDAISDLPAVMNTETRDEMPYQNPPETEFQRYIRSTKYEMTGSKSNGTTEKRPLLYDHRPYFLFEDDYLRVCQIPKRKGANFRDLPGVIVGADNVVRRHPTENPLLPSGKPLVPEYCFTFEHGKSKRPFARLWWDENLPTALTFPSCHNQVVLHPEQDRVLTIREFARLQGFPDYYRFYGTVKERYCQIGNAVAVPVSRALGYALGLACRKLNGNEPLVTLPSKFSHSNYLQLSKCVFGNTSNEVNSRQFRALDAEVTPGSIGQDSRVEDSTQLQTCYNNQPGNTD*

>Gm_MET1(04G187600.1)
MGSASLLNPSQPGVKKNDKSKQKSVVSKTEEEVMFKDKQKKRSLSESSEQTAAMRKMPKRAAACKNLKEKSFLIYEKSCLIETEKDHIVEEESLAVRMTAGQDNGCPNRRITEFILHDETGKSQPLEVLEVDDLFITGLVLPLEASSGKKKEKGVKCEGFGRIESWDISGYEDGSPVIWLSTEVADYDCQKPAASYKKVYDLFLEKARACVEVYKKLAKSSGGDPDISLDELLAGMVRSMSGSKCFSGAASIKDFVISQGEFIYKQLVGLDMTSKANDRMFADIPALIALRDESKKQVHAQVMPSNGSLRIDSGVGDEENKNQMDSVASVNEEDEDAKLARLLQEEEYWQSMNQKKNSRSASASNKYYIKINEDEIANDYPLPVYYKTSLQETDEFIVFDNDYDIYDTQDLPRSMLHNWSLYNSDARLVSLELLPMKPCSDIDVAIFGSGIMTSDDGSGFHLDTEAGKSSSVGSGAQVADGMPIYLSAIKEWMIEFGSSMIFISIRTDLAWYRLGKPAKQYAPWYDTVLKTARLAISIITLLKEQSRVSRLSFGDVIRKVSEFDKKDGSYISSDPLTVERYVVVHGQIILQLFAEFPDDKIRKSAFVTGLTNKMEERHHTKWLVKKKKVVPRSEPNLNPRAAVGPVVSKRKAMQATTTRLINRIWGEYYSNHLPEDAKEGIASELKDEDEVEEQEENEDDDNEETILLEGTPKAHSASKQTKKFSAETEIRWEGEPEGKTSSGYPVYKQAIIRGEVISVGRSVLVEVDETDEFPDIYYVEYMFESKIGRKMFHGRMMQRGCQTVLGNAANEREVFLTNECRDLGLHDVNQTVVVNIQNRPWGHQHRKDNIIADRVDRAQAEERKKKGLPTEYYCKSLYWPERGAFFSLPLDTLGLGSGVCPSCKIQDAEKEKDVFKVNSSKSGFLLKGTEYSLNDYIYVSPFEFEEMIEQGTHKSGRNVGLKAYVVCQVLEIVVKKEIKEAEIKSTQVKIRRFFRPEDVSNEKAYCSDIQEVYYSDETHIISVESIEGKCQVRKKNDIPECSALGRMFQNVFFCELLYDPATGSLKKLPAHVKVKYSSGQTSDAAARKRKGKCIEGDDVLESPNEGKTLNEKRLATLDIFAGCGGLSEGLQQSGVSSTKWAIEYEEPAGDAFKANHPEALVFINNCNVILRAVMEKCGDTDDCISTSEAAELAAKLDEKEISSLPMPGQVDFINGGPPCQGFSGMNRFNQSSWSKVQCEMILAFLSFADYFRPRYFLLENVRNFVSFNKGQTFRLTLASLLEMGYQVRFGILEAGAYGVSQSRKRAFIWAASPEDVLPEWPEPMHVFSAPELKITLSENVQYAAVRSTANGAPLRSITVQDTIGDLPAVGNGASKGNMEYQNDPVSWFQKKIRGDMVVLTDHISKEMNELNLIRCQKIPKRPGADWRDLPEEKIKLSTGQVVDLIPWCLPNTAKRHNQWKGLFGRLDWQGNFPTSITDPQPMGKVGMCFHPDQDRILTVRECARSQGFPDSYQFAGNIIHKHRQIGNAVPPPLASALGRKLKEAVDSKSST*

>Gm_MET2(06G178200.1)
MGSASLLNPSQPGVKKNSKSKQKSVVSKTEEKVMVKDKQKKRSLLESSEQPAATRKMPKRAAACKNLKEKSFLISEKSCLIEMEKDQIVEEESLAVRMTAGQDDGRPNRRITEFILHDATGKAQPLEVLEVNELFITGLILPLEVSTGKKKEKGVKCEGFGRVESWDISGYEDGSPVIWLSTDIADYDCQKPAASYKKVYDLFLEKARACIEVYLKLAKSSGGDPDISLDELLAGMVRSMSGSKCFSGTASIKDFVISHGEFIYKQLIGLDMTSKANDRTFADIPALIALRDESKKQANYVHAQVMPSNGSLRIDSGVGDEENKNQMDSVASVNEEDEDAKLARLLQEEEYWQSMKQKKNSRPTSVSNKYYIKINEDEIANDYPLPAYYKTSLQETDEFIVFDNDYDIYDTQDLPRSMLHNWSLYNSDARLVSLELLPMKPCSDIDVAIFGSGIMTSDDGSGFHLDTEAGQSSSVGSGAQVADGMPIYLSAIKEWMIEFGSAMIFISIRTDLAWYRLGKPAKQYAPWYDTVLKTARLAISIITLLKEQSRVSRLSFGDVIRKVSEFNQKDGSYISSDPLTVERYVVVHGQIILQLFAEFPDDNIRKSSFVTGLTNKMEERHHTKWLVKKKKVVPRSEPNLNPRAAVGPVVSKRKAMQATTTRLINRIWGEYYSNHLPEDSKEGIASELKDEDEVEEQEENEDDDNEEETILLEGTPKAHSASKQTKKISADTEIRWEGEPEGKTSSGYPVYKQAIIRGEVISVGRSVLVEVDETDEFPDIYYVEYMFESKIGRKMFHGRMMQCGCQTVLGNAANEREVFLTNECRDLGLHDVKQTVVVNIQNRPWGHQHRKDNIIADRVDRTQAEERKKKGLPTDYYCKSLYWPERGAFFTLPHDTLGLGSGVCPSCKIQDAEKEKDVFKVNSSKSGFLFNGTEYSLDDYVYVSPFEFEEKIEQGTHKSGRNVGLKAYVVCQVLEIVVKKEIKQAEIKSTQVKIRRFFRPEDLSNEKAYCSDIREVYYSDETYIISVESIEGKCQVRKKNDIPECSALGGIFQNVFFCELLYDPATGSLKKLPAHIKVKYSSGQTSDAAARKRKGKCIEGDGVSESTKEGKTLNDKRLATLDIFAGCGGLSEGLQQSGVSSTKWAIEYEEPAGDAFKANHPEALVFINNCNVILRAVMEKCGDTDDCISTSEAAELAAKLDEKEISSLPMPGQVDFINGGPPCQGFSGMNRFNQSSWSKVQCEMILAFLSFADYFRPRYFLLENVRNFVSFNKGQTFRLTLASLLEMGYQVRFGILEAGAFGVSQSRKRAFIWAASPEDVLPEWPEPVHVFSAPELKITLSENVQYAAVRSTANGAPLRAITVRDTIGDLPAVGNGASKGNMEYQNDPVSWFQKKIRGDMVVLTDHISKEMNELNLIRCQKIPKRPGADWRDLPEEKIKLSSGQVVDLIPWCLPNTAKRHNQWKGLFGRLDWQGNFPTSVTDPQPMGKVGMCFHPDQDRILTVRECARSQGFPDSYEFAGNIIHKHRQIGNAVPPPLASALGRKLKEAVDSKSST*

>Gm_DRM1(02G035700.1)
MQFHNYVGSEGDDSSPSDYDWNTDDELEVFGIPPSDPTISSQESCEDSVGESSASCSSAKHSKLIHLFVGMGFSRETVIKAIDENGRDNEEDIMEALLTLTAEKPLTVEKDEALSVLVNMGYPFEEALTAIDKCGPKAHISELADFISASQLEKGLHSPQESPNNKHDASDYTHEKPCQPSGEYYLHTSKKVKLGLGIFNEASQVISRKFPREVANKPYFYFENVALAPKGVWKTISRFLYEIEPEYVDSKYFCAATRKRGYIHNLPTHNRSPLLPIPPLTIQEAFPTTKKWWPSWDRRTKLNCLLTRVAPGPVTERIRKLLEKFGDEPPLHVQENVLVEIRKWNLVWVGKNKLAPLEPDEYEMLLGFPRDHTRGGGVTRTERYKSLGNAFQVNTVAYHLSVLKGRFPNGINVLSLFSGIGGAEVALHRLGMMLKNVVSVEIAEVNRNIIRSWWEQTNQRGNLIEVEDVQKVSSNELSQWITKFGGFDLIIGGSPCNNISGSNRVSRHGLEGEQSSLFYEYFRIVEAVMEIQRDELL*

>Gm_DRM2(05G005600.1)
MGGDDSGLESDNFDWNTEDELEIQNYNSSSSCLTLPNGDAVTGSGEASSSAVLANSKVLDHFVSMGFSREMVSKVIQEYGEENEDKLLEELLTYKALESSSRPQQRIEPDPCSSENAGSSWDDFSDTDIFSDDEEIAKTMSENDDTLRSLVKMGYKQVEALIAIERLGPNASLEELVDFIGVAQMAKAEDALLPPQEKLQYNDYAKSNKRRLYDYEVLGRKKPRGCEKKILNEDDEDAEALHLPNPMIGFGVPTESSFITHRRLPEDAIGPPYFYYENVALAPKGVWQTISRFLYDVEPEFVDSKFFCAAARKRGYIHNLPIQNRFPLLPLPPRTIHEAFPLTKKWWPSWDIRTKLNCLQTCIGSAKLTERIRKAVEIYDEDPPESVQKYVLHQCRKWNLVWVGRNKVAPLEPDEVETLLGFPRNHTRGGGISRTDRYKSLGNSFQVDTVAYHLSVLKEMYPNGINLLSLFSGIGGAEVALHRLGIPLKNVVSVEKSEVNRNIVRSWWEQTNQKGNLYDMDDVRELDGDRLEQLMSTFGGFDLIVGGSPCNNLAGSNRVSRDGLEGKESSLFFDYFRILDLVKNMSAKYR*

>Gm_DRM3(07G233200.1)
MAGNPNRREGKTVMVPKTENLDYELPPYTSFSGDVGDNVASSSGGKLRAFFIGMGFLPCLVDKVIEENGEENSDILLEALLRYSALQKSNSQSSVSLDSLFDDKDPPEISNVNQAKEEPDELSGVVDDTRGSLLMMNFSVEEVEFAIHKLGDEASIPELVDFIFALQIAKKLKKEPDDITFTYYGRGNEVTNEKLFGIMAKTLQLFEMGFSENEVSSAIDKLGSEAPISELANFIFAEQNGIDYVMEYKFPTTSTYSVGIKEEPEMDLYGTAEVKVEGFSHEPPQSSQVNLEETYNDDMVKEEEGIDAFPSNVSDQYLDVVENGRGKRPKYEHDDDPVNCLEPSWVEERVDAVVAEMSRHPKPNPSRCLSSVAAKPPFFLFGNVSNISYDSWTKMSQFLYGIEPEFANAQSFSAMDRIEGYIHNLPVENRFHILPKPPMTIEDAMPQTKKWWPPWDSRKLLSSIYCETNGIAQTCDRLGNFLADSGGVLTSEQQKDILRYCRRLNLVWIGKFKLGPVEPEQLELILGYPLNHTRATEGNVAERLKSLKYCFQTDTLGYHLSVLRPIFPHGLTMLSLFSGLGGAEIALHRLAIKIKAVVSVETSETKRKILEKWWRQSGQTGTLVQIEDIQKLTSKKLEGLISKFGGFDLVIYQNPCSYSSSRLQAGVGLSALDFSVFCECVRVLQRVRGMYQRK*

>Gm_DRM4(17G038300.1)
MAGNSNRREGKPVMVPKTEDLGYELPPYTSFSGDVGDNVASSSGGKLRAFFIGMGFLPCLVDKVIEENGEENSDTLLEALLRYSAHKSNCDSFDSLGVSHNTSRGRSAPNFYPDGHSKEALQKSNSQSSDSLDSLFDDKDPPEISNVNQAKEEPDELSGVIDDKRGSLLMMNFSVEEVELAIHKLGDEASIPELVDFIFAWQIAKKLKKEPDDITFTYYGRGNEVTNEKLFGIMAKTLQLFEMGFSENEVSSAIDKLGSEAPISELANFIFAEQNGIDYVMEYKFPTTSTYSVGIKEEPEMDLYGTAEVKVEDFSNEPPQSSQVNLEETYNEDMVKEEEGIDAFPSYVSDQYLDVVENGRGKRPKYEHDDDSITCLEPSWVEERVDVVVAEMSRRPKPNPSRCLSSVAAKPPFFLFGNVSNISYDSWTKMSKFLYGIEPEFANAQSFSALDRIEGYIHNLPVENRFHILPKPPMTIEDAMPLTKKWWPPWDSRKQLSSIYCETNGIAQTCDRLGNILADSGGVLTSELQKDILRYCRGLNLVWIGKFKLGPVEPEQLELILGYPLNHTRASEGNVAERLKSLKYCFQTDTLGYHLSVLRPIFPHGLTMLSLFSGLGGAEIALHRLGIKIKVVVSVETSETKRKILERWWRQSGQTGTLVQLEDIQKLTSKKLEGLISKFGGFDLVIYQNPCSNLSSRLLAGVGLPALEFSVFCECVRVLQRIRGMYQRK*

>Gm_DRM5(19G006100.1)
MGGDDFDWNTDDELEIENYNSSSSCLTLPNGDAGEASSSAVLANSKVLDHFVNMGFSREMVSKVIQEYGEENEDKLLEELLSYKALESSPQPQQRIEPDPCSSENAGSSWDDFSDTDIFSDDEEIAKTMSENDDTLRSLVKMGYKQEEALVAIERLGPNASLEELVDFIGVAQMAKAEDALLPPEEKLQYNDYAKSNKRRFYDYEVLGRKKPRGCEKKILNEDDDEEDEALHLPNPMIGFGVPTESSFITHRRIPEDAIGPPYFYYENVALAPKGVWQTISRFLYDVQPEFVDSKFFCAAARKRGYIHNLPIQNRFPLLPLPPRTIHEAFPLTKKWWPSWDTRTKLNCLQTCIGSAKLTERIRKAVEIYDEDPPESVQKFVLHQCRKWNLVWVGRNKVAPLEPDEVETLLGFPRNHTRGGGISRTDRYKSLGNSFQVDTVAYHLSVLKEMYPNGINLLSLFSGIGGAEVALHRLGIPLKNVVSVEKSEVNRNIVRSWWEQTNQKGNLYDIDDVRELDGDRLEQLMSTFGGFDLIVGGSPCNNLAGSNRVSRDGLEGKESSLFFDYFRILDLVKNMSAKYR*

>Os_MET1_LOC_Os03g58400.1

MDKCGDSDDCISTSEAAERAAKLSEDKIKNLPVPGEVEFINGGPPCQGFSGMNRFNQSPWSKVQCEMILAFLSFAEYFRPRFFLLENVRNFVSFNKGQTFRLTLASLLEMGYQVRFGILEAGAYGVAQSRKRAFIWAAAPGETLPEWPEPMHVFASPELKITLPDGKFYAAVKSTAAGAPFRSITVRDTIGDLPAVENGAGKPTIQYGSGPVSWFQKKIRSDMASLNDHISKEMNELNLIRCKHIPKRPGCDWHDLPDEKVKLSTGQMVDLIPWCLPNTAKRHNQWKGLYGRLDWEGNFPTSVTDPQPMGKVGMCFHPEQDRIITVRECARSQGFPDSYRFAGNIQNKHRQIGNAVPPPLAYALGRKLKQAIDAKR*

>Os_MET2_LOC_Os07g08500.1

MDKCGDADDCISTSEAAEQAAKFSQDNIMNLPVPGEVEFINGGPPCQGFSGMNRFNQSPWSKVQCEMILAFLSFAEYFRPRFFLLENVRNFVSFNKGQTFRLTVASLLEMGYQVRFGILEAGTFGVAQSRKRAFIWAAAPGETLPDWPEPMHVFASPELKINLPDGKYYAAAKSTAGGAPFRAITVRDTIGDLPKVENGASKLLLEYGGEPISWFQKKIRGNTIALNDHISKEMNELNLIRCQRIPKRPGCDWHDLPDEKVKLSSGQLVDLIPWCLPNTAKRHNQWKGLYGRLDWEGNFPTSVTDPQPMGKVGMCFHPDQDRIITVRECARSQGFPDNYQFAGNIQSKHRQIGNAVPPPLAFALGRKLKEAVDAKRQ*

>Os_CMT1_LOC_Os03g12570.1

MVPEPAPAAATEPRRSTRRRLMTAAAMEAEAEAVADLDEIDREMSRAESRKRQRRTAKEKPGARKGATEWKPEDVEKAAAAEGVAELDEIDREMPRPELRKRQRRTAKEKPSAHEGATEWKPEDVEKAAAQEPEGTELDSGLSPAESRGKRQRGVEKVKRRTRKKTAKEKTKETTEKSAAQAPEKMKVNDAGGALAEDVCADEPDAEQMAMEEEEEAADVLEAEERMGKCVGEGSAEKAATRKRVARPSTARRVEDSDDHFVGDPVPDDEARQRWPVRYSRKGSDSLLKQEPDEDEEMKARCHYLAANVDDEIYHLDDDVYVKAGPDEENYIGRITEFFEGVDRGSYFSCQWFFRTADTVISSKLLKVHDHRHNHKRVFLSKEKNDNLIECIVSKVKIAHVDPNMTPQARAHAISDCDLYYDMSYSVAYSTFANLPADNDGALGSEATSNISCDDADNSSKGKLSADIVAPYSEQTETASLLDLYSGCGAMSTGLCLGFAFSGINLETRWAVDINKYACACLKHNHPYSQVRNEKTEDFLALIQQWDALCRKYVVHKNDTLEPSIDMPLNDADDVNEPLPEDIFDVEELLEICYGDPSNTGKNGLWFKVRWKGYDPSYDTWEPIDGLSDCPERIKEFVEKGHKENILPLPGAVDVICGGPPCQGISGFNRFRKHNDPLEDEKNKQLVVFMDIVKYLRPKYVLMENVVDILKFADGFLGRYAMSCLVAMNYQARLGMMAAGYYGLPQFRMRAFLWGALPSMVLPKFPLPTHDAVVRGIVPTTFSQSVVAYNEVDTRCLRKALLLADAISDLPKVGNDQPKDVIEYSVAPKTEFQRYIRNNRKDIQDYSFRGDDPSEEGKLFDHQPLKLNKDDYERVQRIPVKKGANFRDLKGVIVGPDNTVRLDPNISRERLSSGKPLVPDYAISFVKGKSTKPFGRLWWDETVPTVVTRAEPHNQIILHPSQDRVLTIRENARLQGFPDYYRLIGPLKEKYIQVGNAVAIPVARALGYALGLAYRGESDGDRAVLKLPESFIYADQETVVKSSAGTPGSEIADSEQLFEYFINPNFFCMMFAFLKVNVVIPLILDSKMGLSRRGAEEGEARRAVPFQNILSWEGWNRLDHRRGEIRLDVRREMDDSPLDNLFDGNGLDSSAGADSSSSTGSPPSSTSSSPPSSQSPPPGSSPPPASPPPSTPSAPPTNSSGSAPSPPSPSQSAPPANTGGGGSPPPSHGSPPAPKAVQSQPAPKRSGDGGSSSDSGSSKEGGSSSDRGKSESNGNRPGPEAAIIAGMVIGFFTFALLLAIVACVCCSKKKKRPPHMHMPYYTDENGKVYYANSMPRWQNSVDQGGGWHAQYSPGQAPPSSEMSGSHGAGPLPPPSPGMALGFSKSSFSYDELALATGGFSSANLLGQGGFGYVYRGVLAGSGKEVAVKQLKAGSGQGEREFQAEVEIISRVHHRHLVSLVGYCIAGSSQRLLVYEFVPNDTLEHHLHGLHIFPALFPAIVTRHSEGRAGDGLDDEARHRARLSQGPRVPSRRFLQGQILSVRPSNDLICCIVVVTYEGHPRIIHRDIKAANILLDENFDAKVADFGLAKLTTDTNTHVSTRVMGTFGYLAPEYASSGKLTDKSDVFSFGVMLLELITGRRPVDPTNYMEDSLVDWARPLLARALSEDGSFDELIDQRLENKFDRLEMERMAACAAAAVRHSAKRRPKMKQIVRALEGDASLDDLNEGVKPGQSMMFSTGSEYDSGNYASDINRLRKVAFESSIEDSSEYGTHSSADSGEPPRRQQHR*

>Os_CMT2_LOC_Os05g13780.1

MEASVSQIRRSPPRIEKRKKKNHNSLSGKAPPTRGNCDSLPPVWNAKSASECHWTRRLTSNYLSLVVVVVGEELPPCFAPPPMETPPPDPVSPPPPAADEGSPGGDDGAEDAGGFSAGLDSLWTALFGSPEELEPMWSPPRGFGVGAEFAAAEVEPEIMDVAGGPWDGAPWRSSGVVAGEGAATALVPPTAAAGFAEFEPAAPIDSYPAGAAAASLGDVPEVSALDSGVDCSPDPPPSSSPPVDFDARGFDPVADSAPAMESPLPPSVASSEANLDGRMLDCTLNSVPSPPLASPYEVGLGAEDPIKDSSPSVAWGTTMDAKDPEVDATCANGTALRRSRRIMKIKSAASSMPLNQNGDSSRASKRRVADSRKSRSSEGSKLPAFTGPISVNTVDLINGVKVQGLQEIVAVENVSSSYDNNQKAGGLYNQVVVALPAASNSLLKDKGASVLPRRKTRLASKVLVNSDRVSAISPVVNGGPPVQKSDVCIPTKKHKLAVEECLTSLDGVDGGGIVLCNSKLKSAKSRVVSKTPQGRGRRSPQPPKTQRARTLSVKYLEKLKRAENNNNNGSMSKSPRVPMIPENNGSMSKSPRVPIIPELSTKHELVLDKHMVDSVMLETDDGSCFFVGDAVPDDEARKQWPHRYEINDQIMKKDKRTSSQTFAKLVTVSFCVVFMYVYLQMLDFRGPEGKPNYIGRLLEFFETKTGECYFRVQWFFTAEDTVIGEQAQSHDPRRLFYSDLTDDNLLDCIVSKVTIVQVPPSVDGKSKSVPSSDYYYDMKYSIDYSTFSTIEMEDTDDLMQSCYTSRINDKMKKIDVNKKHKSPVLEKMELSLLDLYCGCGGMSTGLCLGARGGGVNLSARWAIDDDEIACESFRNNHPETRVRNETTDDFLELLKEWEKLCKTYVKHSRTKACVDSTTESNNETPDCSTVPPEEFEVWKLVDICFGDPNKVSKHGLYFKVRWKGYGPHHDTWEPVEGLRNCKEAIRDFVIEGHRQRILPRPRRNIAVFLLRPSKFPLGDVDVVCGGPPCQGISGYNRNREFEAPFKCEKNKQIIVFMDVVQFLKPKYVYMENVLDILKFADATLARYALSRLVAMHYQARLGIMAAGCYGLPQFRMRVFLLGCHSKEKLPPFPLPTHEAIVKNGCPLAFERNLVGWPNDTPMQLARPIVLEDILSDLPEVANGESRDEMLYVKGPQTEFQRYIRSFNVEVHGPRAHVTKDSKSSKLYDHRPLVLDNDNYQRILQIPKRKGANFRDLSGVIVGPDNVARLDPTKERVLLPSGRPLVLDCILAYENGKSLRPFGRVWWDEVVGTVLTVPNARMQALIHPAQDRLLTIRESARLQGFPDNYRFRGTVKDRYRQIGNAVAVPVGRALGYALAMAYLKKSGDDPLMLLPPNFAFSHDLRGFA*

>Os_CMT3_LOC_Os10g01570.1

MVTFMDIVAYLKPKYVLMENVVDILKFADGYLGRYALSRLVAMKYQARLGMMVAGCYGLPQFRMRVFLWGALPTMVLPKYPLPTHNVVVRGGAPNAFSQSIVAYDETQKPTLKNALLLGDAISDLPEVNNHQPNEVMEYGSSPKTEFQRYIRLSRKEMLDSSFEGKDGPDLGKLLDHQPLKLNKDDHERVQQIPVKKGANFRDLKGVRVGANNIVEWDPDVPRVYLSSGKPLVPDYAMSFIKGRSLKPFGRLWWDETVPTVVTRAEPHNQIILHPNQARVLTVRENARLQGFPDYYKMFGPIKEKYIQVGNAVAVPVARALGYSLGLAYQRESEGSSPLFVLPDSFTEVGRQAAPARASSVGIPVGEVVEQ*

>OsDRM1a_LOC_Os11g01810.1

MRIASSSGILMDANGKANGSAPSALVAYFLGMGFSREMVFRAIKEIGDTDSEQILELLLTYQAIGSDPSVGNSSHSACDPQILEEEDEEEDVNWDEDDTVDNFDRATYSDGSGDEDFLQEMSEKDEKIKSLVSMGFPEDEDTEFSSFGGRKKTKLIDGSKKKRERYRSRPQWNQVPFDGSHEEPMPLPNSMVGFSLPNDGLRSVHRNLPDQALGPPFFYYENVALAPKGVWTTISRFLYDIYPEFVYSKYFCAAARKRGYIHNLPIKNRNYTRGVSRTARYRALGNSFQVDTVAYHLSVLRDIFPNGMNVLSLFSGIGGAEVALHRLGICMKTVVLVEISEVNMTLLRSWWDQTQTGTLIEIADVQNLTAERIELFIRRFGGFDLVIGGSPCNNLAGSNRYHRDGLEGKHSALFYHYYRILDSVKTIMASIFGAKGKLFRHVRKALLLKQSSSLTLKTEQDPSNNSDKDSMDK*

>OsDRM2_LOC_Os03g02010.4

MQAIGGDASVGNCSASACAPQTLEVDEEEDDTNWDEYDTAGNCDRTPHSDGSGDEDFFQEMSEKDEKMKSLVNMGFPEDEAKMAIDRCLDAPVAVLVDSIYASQEAGNGYSANLSDYEDTEFSSFGGRKKTRFVDGSKKRKRYGSGPSGNQVPFDGSHEEPMPLPNPMVGFSLPNERLRSVHRNLPDQALGPPFFYYENVALAPKGVWTTISRFLYDIQPEFVDSKYFCAAARKRGYIHNLPIENRSPVLPMPPKTISEAFPNTKRWWPSWDPRRQFNCLQTCMASAKLTERIRCALGRFSDVPTPQVQKYVLDECRKWNLVWVGKNKVAPLEPDEMEFLLGYPRNHTRGVSRTERYRALGNSFQVDTVAYHLSVLRDLFPNGMNVLSLFSGIGGAEVALHRLGIHMKTVISVEKSEVNRTILKSWWDQTQTGTLIEIADVRHLTTERIETFIRRFGGFDLVIGGSPCNNLAGSNRHHRDGLEGEHSALFYDYIRILEHVKATMSAV*

>Sl_MET1_Solyc11g030600.2.1

MASPQPNSESVLELPNNDKSGHKKNKRKQDSVSKRKASATGKKEKKQAVSETIEEPTAGRKRPKRAAACSDFKEKSVHLSKKSSVIETKKDHCVDEEDVAIRLTAGLQESQRPCRRLTDFVFHNSEGIPQPFGMSEVDDLFISGLILPLEDSLDKVKAKGIRCEGFGRIEEWAISGYEDGTPVIWISTETADYDCLKPSGSYKKFYDHFLAKATACVEVYKKLSKSSGGNPDLSLDELLAGVVRAMTGIKCFSGGVSIRDFVITQGGFIYKELIGLDDTSKKTDQLFVELPVLASLRDESSKHETLAQPETISSGNGLRIGPKAGNGGDKIVESGLANGPAPEDEDLKLAKLLHEEEYWCSLKQKKDRNTSSSSSKIYIKINEDEIASDYPLPAYYKTSNEETDEYIVFDSGVETYHIDELPRSMLHNWALYNSDSRLISLELLPMKACADIDVTIFGSGVMTADDGSGYNFDTDANHSSSGGSRSAEIDGMPIYLSAIKEWMIEFGSSMIFISIRTDMAWYRLGKPLKQYAPWYEPVIKTARLAVSIITLLKEQNRVARLSFGEVIKRVSEFKKDHPAYISSNVDAVERYVVVHGQIILQQFSEFPDVSIRNCAFAVGLSRKMEERHHTKWVIKKKKVMQRLEQNLNPRASMAPSVKRKAMQATTTRLINRIWGEYYSNYSPEVSKEVADCEVKDDEEPDEQEENEEDDVPEENLDVPEKAHTPSSTRRHIKSRSDSKEINWDGESIGKTASGEQLFKKARVHGHEIAVGDSVLVEHDEPDELPSIYFVEYMFEKLDGSKMLHGKMMQRGSDTVLGNAANEREVFLINECMNLQLGDVKESIAVNIRMMPWGHQHRNTNADKLERAKAEDRKRKGLPTEFYCKSFYRPEKGAFFRLPFDKMGLGNGLCYSCELQQTDQEKESFKFDMSKSSFVYLGTEYSVDDFVYVSPDHFTAERGGNGTFKAGRNVGLMAYVVCQLLEIVGPKGSKQAKVDSTNVKVRRFFRPEDISSDKAYSSDIREIYYSEDIHTVPVEIIKGKCEVRKKYDISSEDVPAMFDHIFFCEYLYDPLNGSLKKLPAQINLRLSKIKLDDATSRKRKGKGKEGVDEVGELNETSPQNRLSTLDIFAGCGGLSEGLQHSGVTDTNWAIEYEAPAGDAFRLNHPKTKVFIHNCNVILRAVMQKCGDSDDCISTPEASELAAAMDESELNSLPLPGQVDFINGGPPCQGFSGMNRFNQSTWSKVQCEMILAFLSFADYYRPKFFLLENVRNFVSFNQKQTFRLTVASLLEMGYQVRFGILEAGAYGVPQSRKRAFIWAASPEEVLPEWPEPMHVFAVPELKIALSETSYYAAVRSTASGAPFRSLTVRDTIGDLPVVGNGASKTCIEYQGDPVSWFQKKIRGSSITLSDHISKEMNELNLIRCQRIPKRPGADWRDLEDEKVKLSNGQLVDLIPWCLPNTAKRHNQWKGLFGRLDWDGNFPTSITDPQPMGKVGMCFHPDQDRIVTVRECARSQGFPDSYQFAGNILHKHRQIGNAVPPPLAYALGRKLKEAVESKNRLT*

>Sl_DRM7_Solyc04g005250.2.1

MDNNFSGEDNDSIDWDTEDELEIQEMPDATFSSCTNLRSVGYHTVSGHREARSSSEPCQSKFIQQFIVMGFPEESIAKAIEQNGENEGLVLDALLTFKALEDSPEEQPSTSTQMEPCITSDDSSSQYNENFLDDVSEDDSWSLDSDNCVNSAKQSYLNDDNCSLSENEKTLLFLANMGYPAEEASIAMERCGPEAPFPELIDFMCAAQMAREEDVHLPEDEKPKLNSGGYKRKMYNEVRVKKKQRAITDEETIHLPRPMIGFGVPTESLPAVVKRTLPEQAIGPPFFYYENVALAPKGVWDTMTRFLYDIEPEFVDSKYFCATARKRGYIHNLPIEDRFPLLPLPPRTIHEAFPLTKKWWPSWDTRTKLNCLQTSIGSARLADRIRKAMKAMENFDSEPPLMVQKYVLDECRKWNLLWVGRNKVSPLETDEFEMLMGFPRNHTRGGGISRTDRYKSLGNSFQVDTVAYHLSVLKDLYPNGINVLSLFSGIGGAEVALYRLGIPLNNVVAVEISEVNRNILRSWWEQTNQKGNLIDFHDIQQLNGDVLEQMIDSIGGFDLVIGGSPCNNLTGSNRVTRDGLEGKDSSLFYDYVRVVDLVKSIMSNRRV*

>Sl_DRM6_Solyc10g078190.1.1

MDKNLSGEDNDDIDWDTEDELEIQEIQDTVFSSCTDLRTTGQHVVCCDVEASSSSVPFRSKFIQQFVVMGFPEESIAKAIEQNGENSDLVLDSLLTFKALDDSPEEQPSVSPPLEPSISSDDSASEYNKIVLDNVYEDDSWSSDSDNYINTVKQCYLNDEGSSLSEKEKMLLFLGNMGYPAEEASIAMDRCGPKASLPELVDFICAAQMSRAEDPYLLEDVKPNLKDILNDCGGYKKRKMYNELCKRKKQREISVEEPIRLPKPMIGFGIPTESVPRMVQRILPEKIIGPPYFYYENVALAPKGVWDTIKRHLYEIEPEFVDSKYFSATARKRGYLHNLPIENRFPLFPLPPRTIHEALPLSKRWWPSWDTRSKLNCLQTAIGSARLTDKIRKAVEKYDGEPPMEIQKYVLYHCKKWNLVWVGRNKVAPLEPDEVEMLLGFPKNHTRGGGISRTDRYKSLGNSFQVDTVAYHLSVLRDLFPNGINVLSLFSGIGGAEVALYRLGVPLNNVVSVEKSEVNRNIVRSWWEQTNQRGNLIHFDDVQLLSRDRLKKLIESVGGFDLVIGGSPCNNLAGSNRVSRDGLEGKESSLFFDYVRILDDVKSIMSRHR*

>Sl_DRM5_Solyc02g062740.2.1

MDKHLSEEDSDNIDWDTEDELEIQDTTFSSCRDLRTNGQYAISGDGEASSSSVPGQSTFIQKFLVMGFSEESIAKAIEQNGENSDLVLDALLTLKAIEDSPEEQPSASPHLEPCINSDDSSSEYNENFLDDVYDEDSWSSDSDYCTNSVKQCYVKEESNSLSEKEQTILFLANMGYPVEEVSIAMERCGPEASVSELTDFICAAQMAREEDPYLPEDVKPKLNHGSGGYKKRKMFNQLCKSKKPRAIFDEETIRLPKPMIGFGVPTESVSAIVRRTIPEQAFGPPFFYYENVALAPKGVWDTISRFLYDIEPEFVDSKYFCATARKRGYIHNLPTENRFPLLPLPPRTINEALPLTKKWWPSWDPRTKLNCLQTAIGSARLTDRIRKAVEAFDGEPPMRVQKFVLDQCRKWNLVWVGRNKVAPLEPDEFEMLLGFPKNHTRGGGISRTDRYKSLGNSFQVDTVAYHLSVLKDMFPNGMNVLSLFSGIGGAEVALYRLGIQLNNVVSVEKSEVNRNIVRSWWEQTNQRGNLIDFDDVQQLNGDRLEQLIDSCGGFDLLIGGSPCNNLAGSNRVSRDGLEGKESSLFYDYVRILDLVKSIMSRQR*

>Sl_CMT4_Solyc08g005400.2.1

MAKKQNDSSRFSTNLTPSPQKDKPISSSSELALFYPQNEEPVPLIVFYPSSVRRRSSRFTTNKFSTSATEFMTKNRDTIS

PEKTVFLLPSPPTLAGKKTPAEVTRRSPRLVSLSARTTTAKEKGKKVNSRKSEGGIKQVELSRKRKPASCKMDEETRKSP

RFNSDSSNGVQLALPEMSACGALSAGGRTGTKRELLALMTTTPANSSASRKRAARGSDSVNVGNNSRGSRRKDPVFAESP

GTSVKITAESNSAGEKNLRSRKAQGSVNYNESKGSETKRIKSSAEKSVRKQKSNACFIGEPIDTEEAQQQWQWRYELKNR

KTQRQGWKLNSGEEDEIILNVECHYAQAKVAGFIFNIGDCAFVKGEGKKKHIGRILEFFKTTEGEDYFRVQWFFRAEDTV

LQGAASFHDPKRVFYSTLENDNLLDCIVSKVNVVELPTRHDLNKKDVPPAHFYYDMEYCVDYSTFRTLHNVKSSVSPSLV

DVSYKPITTYPLEVSPSCEPMKVELSLLDLYAGCGGMSMGLCLGTKLSGLNLVTKWAVDFNKAACDSLKLNHPQTHVRNE

GVENFLELLKRWEKLIKSYGCSDIKTSSNIELDDRDEGENNDDSQSGSNASSGEYEVLRFVDICYGDPNNDGKSGLHFKV

RWKGYGPSEDTWEPIENLKNCGDSIKDFVRRGQQLKILPLPGDVDMICGGPPCQGISGYNRHRNTDDPLSDEKNRQIIIF

MDVVEFLRPKYVLMENVADILRFDKASLGRYALSRLVHMRYQARLGTMAAGCYGLPQFRLRVFFWGALPSERLPPFPLPS

HDVIVKYWPSPEFERNTVAYEEGQPRDLEEALVLRDAISDMPAVTWHETREERPYEMPPETEFQKYIRLSKHEILSCTST

GVKETKEPVLSDHRPCQLNEDDYLRVCLVPRRKGANFRDLPGVIVGGDNVARRDTKDPKVLPNGKPMVPDCAFNFEHGKS

KRPFARLWWDETVATLVTFPNHRAQAILHPEQDRVLTIREYARLQGFPDFYRFTGTLKERYCQVGNAVAVPVGRALGYAL

GLAYQRLAGNEPLIKLPSNFSFLTPPIDDIVVLQT*

>Sl_CMT3_Solyc01g006100.2.1

MSSKRKASPADSSSDSSKRHALEVVKTVDIASDEVAEGFRDDDEFVEDRDIVCDSSIGESSGQKEVRRVAVRANEEQEGEFYGEIVLDSEARKKWPHRYILKDNVNINSASMSLNCQHDSDELIQAKCHFAQALVDNVIYKLGDDAYVKAAEDEDDYICKIVEFFQGVDDMKYFTAQWFYRAKDTVIKAHDQFIDKKRVFLSDIKDHNPLDCLVKKIKIVPISSNVSLQFKESLRLECDYYYDMKYLVPFSSFISLPSDVLSPDSESNSTISSDGDVVEVKEQKQEKKLLDLYSGCGGMSTGLCLGADVCDVKLVTKPSNTPTHPLLKVGDANVEDDDEGADDDDGGSGDEDEGEIFEVEEILEVCYGDPKEIKKPGLYFKVRWKGYGPDEDTWEPIEGLDGCQNKIKDFVTDGFKRSVLPLPGQVDVVCGGPPCQGISGFNRFRNSANPLQDPKNKQLEVFMSIVEFLKPRFVLMENVVDLLRFAHGYLGRYALSRLVGMNYQARMGMMVAGAYGLPQFRMRVFMWGALPSEKLPQYPLPTHNVIVRGGIPTEFELNAVDFEEGLKVKLKRELLLEDALSDLPPVENNEPRDEMPYIDEPKSVFQRFIRSRRDGTLGTVLYDHRPLQLNEDDYQRVTQIPKQKGANFRDLPGVRVRADNVVEWDPDMERVKLPSGKPLVPDYAMTFVRGTSQKPFGRLWWDEIVSTVVTRAEPHNQAILHPVQDRVLTIRENARLQGFPDYYKLTGPIKERYIQVGNAVAVPVARALGYSLALALKGLSRDQPLLTLPPNFPCLEELVSNDESLDKV*

>Sl_CMT2_Solyc12g100330.1.1

MPSKRKSSPATKPESSSGSRKSKRLVVERPDPVVAQPSDSDFEPEPVLSSKKKSTRRTTAESSVVACQSESNNKKLKKPTVEKAESGVASPADRDFVSESDSETPSKKSTRRAAVKVEPLVDSVAGSDFVEEEEVDGMELGSLKKSLSISPSKRKPKRAEKVKDEECVLAGDPVPDAEARLKWPHRYNKGKENGTKSLNGQDDPDQLIQAKCHFSRADVDGQIYYLEDDAHVKAADGEDDYICKIVEFFEAVDGVQYFTAQWFYRAKDTVIKSHDQFIDKKRVFLSEIKDDNPLDCLVTKLKIVPVPSNATSQFKENVKSNCDFYYDMKYLLPYSSFISLPPDTTSPVSSSSTISSDIDAGEVKEHNLEKKLLDLYSGCGAMSTGLCLGANSKGVKLVTKWAVDLNKHACDSLRLNHPETQVRNEYASDFLSLLKEWVQLCVSCSLIKGSVPPHPHLKVTDEVDEDEENDDEGEDSGDDKEGEIFEVEELLEVCYGDPKENNKPGLYFKVRWRGYGPEEDTWEPIDGLSDCPKKISEFVVKGFKANLLPLPGDVDVVCGGPPCQGISGFNRFRNKENPMQDPKNKQLDVYMDIVDFLKPRFVLMENVVDLVKFSNGFLGRYALSRLVGMNYQARMGMMAAGAYGLPQFRMRVFMFGALSSEKLPQYPLPTHKVIVRGVIPVEFESNTVAYDSVRDLELKKELFLGDALSDLPLVENNEPRDEMPYTDEPKSDFQHFIRMGRDGLLGSVLYDHRPLQLNEDDHQRVCQIPKRKGANFRDLPGVRVRPDNKVEWDPDVERVKLPSGKPLVPDYAMSFVGGSSSKPFGRLWWDETVPTVVTRAEPHNQTIVHPLQDRVLTIRENARLQGFPDYYKLIGPIKERYMQVGNAVAVPVARALGYSLAMSIKGLSGETPLFTLPKNFPSHEDQNCNEVSQ*

>Sl_Dnmt2_Solyc08g067070.2.1

MESEVSRSPWRVLEFYSGIGGLRYSLLKAAVDATVVEAFDINDVANDVYQHNFGHRPFQGNIQTLNAADLDGYNADAWLLSPPCQPYTRQGLQKGSSDARASSFLKILELVPQLLRPPSYLFVENVVGFETSDTHAILVDILEKNNFVTQEFILSPLQFGLPYSRPRYYCLAKRKPLSFEVPEFNNQLLRTPGPLLGQTESTMEKEQLLSPEYWDELLQACHPVDDFLVFKTFGNRKDSSTYSFHANDSVKSDRLDEENDVCFVPSSLIERWGSAMDIVYPHSKRCCCFTKSYYRYVKGTGSLLATAEATFQEKPENRMPSLQDLSLRYFTPREVANLHSFPEDFQFPQHISLRQRYAMLGNSLSVGVVAPLLQYLFINHS*

>Zm_CMT1_GRMZM2G025592_AFW59438.1

MAPSSPSPAAPTRVSGRKRAAKAEEIHQNKEEEEEEEEVVAASSAKRSRKAASSGKKPKSPPKQAKPGRKKKGDAEMKEPVEDDVCAEEPDEEELAMGEEEAEEQAMREEVVAVAAGSPGKKRVGRRNAAAAAGDHEPEFIGSPVAADEARSNWPKRYGRSTAAKKPDEEEELKARCHYRSAKVDNVVYCLGDDVYVKAGENEADYIGRITEFFEGTDQCHYFTCRWFFRAEDTVINSLVSISVDGHKHDPRRVFLSEEKNDNVLDCIISKVKIVHVDPNMDPKAKAQLIESCDLYYDMSYSVAYSTFANISSENGQSDSDTASGISSDDVDLETSSSMPTRTATLLDLYSGCGGMSTGLCLGAALSGLKLETRWAVDFNSFACQSLKYNHPQTEVRNEKADEFLALLKEWAVLCKKYVQDVDSNLASSEDQADEDSPLDKDEFVVEKLVGICYGGSDRENGIYFKVQWEGYGPEEDTWEPIDNLSDCPQKIRDFVQEGHKRKILPLPGDVDVICGGPPCQGISGFNRYRNRDEPLKDEKNKQMVTFMDIVAYLKPKYVLMENVVDILKFADGYLGKYALSCLVAMKYQARLGMMVAGCYGLPQFRMRVFLWGALSSMVLPKYPLPTYDVVVRGGAPNAFSQCMVAYDETQKPSLKKALLLGDAISDLPKVQNHQPNDVMEYGGSPKTEFQRYIRLSRKDMLDWSFGEGAGPDEGKLLDHQPLRLNNDDYERVQQIPVKKGANFRDLKGVRVGANNIVEWDPEIERVKLSSGKPLVPDYAMSFIKGKSLKPFGRLWWDETVPTVVTRAEPHNQVIIHPTQARVLTIRENARLQGFPDYYRLFGPIKEKYIQVGNAVAVPVARALGYCLGQAYLGESEGSDPLYQLPPSFTSVGGRTAGQARASPVGTPAGEVVEQ

>Zm_CMT2_GRMZM2G005310_DAA36033.1

MAPSSPSSARPTRASGRKRSAMAEEIHQNQEEEEEVVAASTAKRRRKAASSGKKPKPTPKQAKPAVAGMKKKGETEKTEPVVDDVCAEEPDEEELAMGEEEAEAEEQAMQEVVAAVAAGSPGKKRVGRRSAAASGDHVPEFIGSPVAAAEAHSNWPKRYERSTAANKPEEDDELKARCHYRSAKVDNIVYCLGDDVYVKAGENEADYIGRITEFFEGTDRCHYFTCRWFFRAEDTVINSLVSINVDGHKHDPRRVFLSEEKNDNVLDCIISKVKIVHVDPNMDPKAKAQLIEHCDLYYDMSYSVAYSTFANISSENGQSGSETASGISSDDAGLETSSNMPERTATLLDLYSGCGGMSTGLCLGAALSGLKLETRWAVDLNSFACQSLKYNHPQTEVRNEKADEFLALLKEWAVLCEKYVHQDVDSNLAGSEDQEDADTLDKDEFVVQKLIGIRYDGTGRKKGVYFKVQWEGYGPEEDTWEPIDNLSDCPLKIREFVQEGRKRKILPLPGDVDVICGGPPCQGISGFNRFRNRDEPLKDEKNKQMVTFMDIVAYLKPKYVLMENVVDILKFADGYLGKYALSCLVAMKYQARLGMMVAGCYGLPQFRMRVFLWGALSSMVLPKYPLPTYDVVVRGGAPNAFSQCMVAYDETQRPSLKKALLLGDAFSDLPKVENHQPNDVMEYGGSPKTEFQRYIRLGRKDMLDWSFGEEAGPDEGKLLDHQPLRLNNDDYERVKQIPVKKGANFRDLKGVKVGANNVVEWDPEVERVYLSSGKPLVPDYAMSFIKGKSLKPFGRLWWDETVPTVVTRAEPHNQVILHPTQARVLTIRENARLQGFPDYYRLFGPIKEK

>Zm_DRM2_GRMZM2G137366_DAA42884.1

MAHWVSDSDGSDNFEWDSDGNGEEPANFNAAGAGSSALRSTNTDAPGPSTRVANGNGKAGPSASLVQKYMDMGFTEEIVRKAMKDNGDNGADSLVELLLTYQELGNDLKVDNGFASGCAPQTVDDSDDDDILENWDDEDADGGRSTRVANSIDDSEDEDFLNEMSQKDKKVDSLVKMGFPEDEAALAITRCGQDASISVLVDSIYASQTAGDGYCGNLSDYEGNSNGGRNKGRFMDGNKKKRKRYGGQAQGNRGPLDGSCEEPMPLPNPMVGFNLPDHWTRPVDRSLPTQAIGPPYFYYENVALAPKGVWTTISRFLYDIQPEFVDSKYFCAAARKRGYIHNLPLERRSPLLPLPPKTIFEAFPRTKRWWPSWDPRRQFNCLQTCTSSAKLLERIRVTLANSSDPPPPLVQKFVLEECRKWNLAWVGLNKVAPLEPDEMEFLLGFPKDHTRGISRTERYRSLGNSFQVDTVAYHLSVLKDRYPQGMNVLSLFTGIGGAEVALHRLGIRMNTVVSVEKSEVNRTILKSWWDQTQTGTLIEINDVQTLTADRIEAYIRRIGGFDLVIGGSPCNNLAGSNRHHRDGLEGEHSSLFYHYFRILDSVKSIMERL

>Zm_DRM2_GRMZM2G065599_AFW82704.1

MEKTLSLLQMGFTEEEVSSAIDNFDQRATVQELADSILARRIANSIEQKEIKVESDFLDEAETDYSSYQPSYSDVSYYDDDNNNTRVKRAKHIFIDDIGASSSHLGDPWSMGQRAGTNDMSVKVELEAMTPGRRANVQGDLAKPPFFLYGNVVEVPKDTWHQLKQFLYNVEPEFVNSQSFSALTRREGYIHNLPVEKRSVVVPKSPMTIEEALPFTRQWWPSWDTRKHISVITTEAAGIEQTCERLGGMVRESRGVLSQARQMQIIHQCRVSNLIWVGRDRLGPLEPRQVEKILGYPYNHTNLFELNQPDRLAAMKYAFQTDTLSYLLSVLKGKYQDGIRVLSIYSGIGGAEVALHRLGIPLKCVISVEESEVNRKILRRWWLKTEQTGVLRQHAGIWKLKTHVIEDLVKEFGGFDLIIGGNYTSCKGGTTVNTTMGMDSNRFYEYNRVVKRVRTAVGLS

>Fa_MET1a_ACQ91178.1

MGSIAAADDAAAAAAATAPLNDAESAPSALSSGTTKKKGRKATQKEAPAGGRGRKRNVPPTGEEQSQSRKMPKRAAACKDFKERSVRVSEKSAFIESKEEQVVPDESLAVVMTCGKDPDDEKPNRRLTQFILHNEDGVAQPLEMVQHGDLFITGTLLPLDASSDNGKGKGKEKGIICEGFGRIEAWDISGYDEGHPIIWLTTDVADYCCVKPASSYKKHYDNFCEKARACIEVYKVLSKCNSDCSLDELLAGIARSMSGNKFFSGSASIKEFIISQGVFIYNQLEGLEESSKKNDRVLAQLPVLAALRQECIKQGYYVTSNPAASSGTLKIASDGGNSSSKVETEEDEDAKLARLIQEEEYFKSMKQKKRQGVTSMSKKFYIKINEDEIANDYPEPAYYKTAIEETDEFIVVDSEDGDLPTHMLHNWCLYNSDSRLISLELLPMKACADIDVTIFGSGVMSEDDGSCGFDLDSIQSSSSGSVAPVAEDAYGMPVYLSSIKEWVIEWGASMIPVSIRTELAWYRLGKPSKQYAKWYAPILKTARVGRSIITMLKAESRVARLSFADVIKRLSEFPNSNGGYISSDPASVERYVVVHGPILLQLFTEFPDGKIKTCPFVHGLRDKMEKRHHTKWEVNKKILQKSESNLNPRASMGPVVSKRKAMQATTTKLINRIWGEYYSNYSPENSSLVETCEKIEEDEADEEVQEDLEEDDAEENSSVVKEAQRPSPILRKIKSSSSSKKGFVDEEPVGKTSSGEALYKHAVLRGEEISVGGAVMVEVDGSDELPVIYFVEYMYESSDGEKMLHGRMMQRGSDTVLGNTANEREVFLTNECINLALKDVKQTVVVGIKSMPWGHKHQKENADAERIDRANAEERKKKGLPTEYYCKSLYCPEKGAFLSLSCDTIGLGSGFCFSCKANEEEKAKEVFEVNSSKTGFIYSGVEYSVHDYVYVSPYHFSVQTIETELFKAGRNLGLKPFVVCQVLGIITKGSKQSEMKSTQVKVRRFFRPEDISVEKAYCSDVREVYYSEELHILPVDSIEGKCEVRRRSDLPECNAPALFQHIFFCEHLYDPSNGSLKQLPANIRVKYSTVGGDTESRKRKGKCKEGEDVPEVEQQRVDSEHMRLATLDIFAGCGGLSEGLHQAGVSITKWAIEYEEPAGQAFQLNHPESKVFINNCNVILKAVMDKCGDTDDCISTTDANDLASALDEKEKSDLPLPGQVDFINGGPPCQGFSGMNRFNTSTWSKVQCEMILAFLSFADYFRPKYFLLENVRNFVAFNKGQTFRLTLASLLEMGYQVRFGILEAGAYGVSQSRKRAFIWAAAPDEILPEWPEPMHVFGVPELKINLSSNSYYAAVRSTASGAPFRPITVRDTIGDLPAVGNGASKVNMEYESDPVSWFQKKIRGNMAVLTDHISKEMNELNLIRCKRIPKRPGADWKDLPEEKVKLSTGQLVDLIPWCLPNTAKRHNQWKGLFGRLDWEGNFPTSITDPQPMGKVGMCFHPDQDRIITVRECARSQGFPDSYQFYGNTLHKHRQIGNAVPPTLAYALGRKLKEAIDSKKRSSSQE

>Fa_MET1b_ACQ91179.1

MGSIAAADEAATAPLNDAESAPSAPSSGTTKKKGTQKEAPAGGRGRKRNAPPTGEGQSQSRKMPKRAAACKDFKERSVRVSEKSAFIESKEEQVVPDESLAVVMTCGKDPDDEKPNRRLTQFILHNEDGVVHPLEMVQHGDLFITGTLLPLDASSDNGKGKGKEKGVICEGFGRIEAWDISGYDEGNPIIWLTTDVADYCCVKPASSYKKHYDNFCEKARACIEVYKVLSKCNSDCSLDELLAGIARSMSGNKFFSGSASIKEFIISQGVFIYNQLEGLEESSKKNDRVLAQLPVLAALRQECIKQGYYVTSNPAASSGTLKIASDGGNSSSKVETEEDEDAKLARLIQEEEYFKSMKQKKRQGVTSMSKKFYIKINEDEIANDYPEPAYYKTAIEETDEFIVVDSEDGDLPTHMLHNWCLYNSDSRLISLELLPMKACADIDVTIFGSGVMSEDDGSCGFDLDSIQSSSSGSVAPVAEDAYGMPVYLSSIKEWVIEWGASMISVSIRTELAWYRLGKPSKQYAKWYAPILKTARVGRSIITMLKAESRVARLSFADAIKRLSEFPNSNGGYISSDPASVERYVVVHGPILLQLFTEFPDGKIKTCPFVHGLRDKMEKRHHTKWEVNKKILQKSESNLNPRASMGPVVSKRKAMQATTTKLINRIWGEYYSNYSPENSSLVETCEKIEEDEADEEVQEDLEEDDAEENSSVVKEAQRPSPILRKIKSSSSSKKGFVDEEPVGKTSSGEALYKHAVLRGEEISVGGAVMVEVDGSDELPVIYFVEYMYESSDGEKMLHGRMMQRGSDTVLGNTANEREVFLTNECINLALKDVKQTVVVGIKSMPWGHKHQKENADAERIDRANAEERKKKGLPTEYYCKSLYCPEKGAFLSLSCDTIGLGSGFCFSCKANEEEKAKEVFEVNSSKTGFIYSGVEYSVHDYVYVSPYHFSVQTIETELFKAGRNLGLKPFVVCQVLGIITKGSKQSEMKSTQVKVRRFFRPEDISVEKAYCSDVREVYYSEELHILPVDSIEGKCEVRRRSDLPECNAPALFQHIFFCEHLYDPSNGSLKQLPANIRVKYSTVGGDTESRKRKGKCKEGEDVPEVEQQRVDSEHMRLATLDIFAGCGGLSEGLHQAGVSITKWAIEYEEPAGQAFQLNHPESKVFINNCNVILKAVMDKCGDTDDCISTTDANDLASALDEKEKSDLPLPGQVDFINGGPPCQGFSGMNRFNTSTWSKVQCEMILAFLSFADYFRPKYFLLENVRNFVAFNKGQTFRLTLASLLEMGYQVRFGILEAGAYGVSQSRKRAFIWAAAPDEILPEWPEPMHVFGVPELKINLSSNSYYAAVRSTASGAPFRPITVRDTIGDLPAVGNGASKVNIEYESDPVSWFQKKIRGNMAVLTDHISKEMNELNLIRCKRIPKRPGADWKDLPEEKVKLSTGQLVDLIPWCLPNTAKRHNQWKGLFGRLDWEGNFPTSITDPQPMGKVGMCFHPDQDRIITVRECARSQGFPDSYQFYGNTLHKHRQIGNAVPPTLAYALGRKLKEAVDSKKRSSSQE

**Supplementary File S2**

>CcDemethylase-like3

MDLKSEFGYFESFYNGFENQKHHQVDLDSAGYVAAADAAHRLMESLTSSNFSSNDCISISIDFHERLEPIVGLIREEVSPMKLSNDDEDKLVGEKQAMTKMQQVKKFRPKVMTPKPITPSPSSSSSRGHTSVKSSCRRALDFDAQTKTMLIYTKGGHCFTRSVNKYMEGRLECNDKEEDMNEQEEELFRQRALSFITSMRHVQGNRGFMGWKGSVVDSVVGVFLTQNAPDNLSSSAFMCLAAKYLIEDPKEGISKHALDWNAVRCAQPYEISHVIQERGMNNRIAARIQVTFLDSIYNHKSGLLDDLEWLRKAKPEKTMEFFSKIYGLGIKSMDCLRLLTLRQHAFPVDRHVARIVVRLGWVPVEKLPDGVLIHELEEYPMMEAVQDYLSQRLSNLDVDTLYELHYQMITFGKVFCTKKKPNCNSCPLKKECKHFASAFGRFPPQGEFKTFVPRTPTPGQSSSRVQVILEEDIEDLCKVHPVIKVMKNAAGKGKEEEEVVVGRFLVPCRTATRGSFPLDGTFFQINEVFADDESCKKPVVVSRNLLSDLTIKTLFCGTSISAIFQVAAVSIDLVPLVTASHELRLCRLPRVAASHELRLCRLPRVAASQSAVSISPPSYYSFLLLLVPPSASFRLLPVGRSSLDNS

>CcDemethylase-like4

MGEEGESSSSRQVYPPEVAYAPATPAKPDRSDWGPIGIDWLKNQFDEVIFEETSAKKSISCWEGNSVSTSHIYDLSGFSMDDVETWNSISCRDLLALADATIRRGSDDGDHDRSDGLDFDNRSNCIDTQQYGWLNLGNYSPDLNLPPEMVMKPLVSTGLSTQITPGTPDQARRAEHKQMGSDIVAKVVADNNKERYNLDEQPQVQVLVEQLQGDVSTIVEANQDFEKGLTAETNLNETPQPKQRRRKHRPKVVREGQQKKAKQSATPQKPDGSSTGKRKYVRKKGVEKSPATPAVEEGSGTIDPSSDQQNKKSCRKKINFDETEKGNEVTVETAEVNITVDKTCSMNQVVETILESQSASPITPSKTELPIKDAKHTYRKVKCRINFLQETHDKRPSSVSSPNESNCSTSASFNKGEAQGSKMELSSKIVGMELWDENAIGVGCNLSKFTNDCSGGKQGMHLPANKKKRIEKCRSSITSGAISSVCSAQSSDCSFLAEQNASKAPQMSKDYVLKDDQQPYKQAFGHLENSKKKRRSKALSLIPDLALFPGIVEGRHWQTPKEGSRYEVAYQQQTYTEAHAADFHVSIATKKRMKKNAKLPSLYQDHLRFTKGCIDSLINQFERLDINSQMAEEGRDALIPYLSRYNEKNALVLYQERGLVPFEGLFNPVKRRKPRPKVDLDEETSRVWTLLLENINSQGIDGTDEDKAKWWEEERRVFRGRADSFIARMHLVQGDRRFSRWKGSVLDSVIGVFLTQNVSDHLSSSAFMALAARYPLKSKSSSEPLHDDESILSVKEPCQVDQDETITWHEKLNQPSGDHGPMMLQDIDLCEEKEVVNSNKFPKNSGCVDLNVSSEGEVPELAEKDLAMYKESVVDQIENDDIASSQNSANMSPSSVQSSVAHTTERLGSCSKEEQKDMSKATIFGGYTSFVELLHMQGTTTVHETYSQQQAEESSNKKIGQDELESVAYLEEQNDGISPHSNSSRALEVETFELREERNITQKKSQEEFASEESGLSAESASQAMVQLVKTTSSQEASKSCNTCHIRLQERSRSRCKMIAVNPNINTEQHTEDNNCEVQEVIAIANVSADNSKATESNNILKASGETAHKVVEINSVDHGTHQIVNGMDEGSSKVKRQKSGKVKQKIEWDNLRLHAEVTEKRERTPNTSDSLDYEAVRTADVNEVADTIKERGMNNVLAARIKDLLDRLVEDHGSIDLEWLRDVPPDKAKEYLLSFRGLGLKSVECVRLLTLHHLAFPVDTNVGRIAVRLGWVPLQPLPESLQLHLLELYPVLESIQKYLWPRLCKLDQRTLYELHYQMITFGKVFCTKSKPNCNACPMRGECRHFASAFASARLGLPAPEERMASLTENRTGQSSIGLIEQCHITLPSASEQWQQLSDIQNCNSGIEEPATPGSTVEVPATSGPIVEVPATSGTIVEVPATSGPIVEVPATPGPIVEVPETPGPIIEVPATPEPEPIQEEFDIEDFCEDSEEIPMIKLNIEEFTQNLQTYMEKHMVLGEGDMSKALVALTSEAASIPTPKLKNVSQLRTEHQVYELPDSHPLLEGLDTREPDDPCSYLLAIWTPGETADSIQPPEGQCCSQESGTLCSEETCFFCNSTREANSQTVRGTLLIPCRTAMRGSFPLNGTYFQVNEVFADHESSLNPIDVPRSWLWNLPRRTVYFGTSIPTIFKGLTTEDIQYCFWRDVDAVSVVNPRYKWQDNHVQTAAADIQILQKTLSSWPEWHDS

>CcDemethylase-like2

MNFERGFPIPRHGNEDSRSSVTHGIPVPPESGIEHHHGSWTATSGGSGTVGGGDARATPRRPIPMDNGTVHGSESWNTINLEQPTPMGNGVTEDRNPTLERTILQRSGIARNANDWKSVIPGNLFPHRNGIGQSRNSWSPATPEKIVNQRSNTENGLESENWQDLIGMYTGLLKEDTVDKNGVLEDINPTPSKVRDYGNQNWVASNNKNATHRSSPSPYWNHTSNQASTTSDPYLKTNDPANWNSNLLATLVGSQNSSTHTSSANKAQTNGIHHISNRSAVPNSANQVESNSMRSTSWTSMLGSQRTMRFTSNNLINDAHNTEDGFPVAYQPGYKPNSPPRSAASSIIDSFPFAPITPDNQGKHMHSQRVPENGNFRVEGTSTPAKDSRENQTVSREDAENLYNELLQTIGDSPPSAISTTQKELGVPENTDEQGIDLNKTPQQKTPTRRKKHRPKVIREGKPKRTPKPKDPSDGTSNETRVKRKYVRKKGINILETQGDDVTKNIPVASVGKRKYVRKKGLEKFGDEQQSRMDDVATSVVGIPAKSCKKQLNFDLDPVAQDESYGIRSSQQGIDVNVETPARSCKKQLKFDLEPVAQDKGYGIRSSQEGININVNPQDIGQERRINSILERPAIEIAQQNISMQSGNQYELNVPITPLANTKHHALNMLARNMTIKNSIPEHDRRGNLYNKVNQRFHGEGIENLVLQADMVSTNLERVREPNLMSTPQSLASKGMLNLDERRGIKRQSPEQMCLNANTMDSLLLYQKLLLGVAHRAYDRNNLSSILLENSKKTKMQSEFQTLVSSEPSCIIPHKPRQETRQINGIYGNGSAMHLLTSSTEGVNPYKTMHVGGNVINGQFRPPMAATHYLQKHQVFSGMQHHPLRSVPERSQRYIQGHDIGSKTAIMSWNLPPPTPSKETSRYAVTAYPATSLEKRQTAKPNSYNQRLNGLNQMFQHHRNDPLKGYQQPTTVARGRPRKQKPELSVDDITYRLEGLHIYDGNKKEQHELVLYRGSNALIPFEPIKKRIPRPKVDLDPETDRLWRLLMGKEGSEGAETLENGKEKWWEEERRVFRGRADSFIARMHLVQGDRRFSRWKGSVVDSVIGVFLTQNVSDHLSSSAFMSLAAKFPPKLSTTKETCCQDGACEEPIEVAEPNGITKCHEKIKQPVPDQSFFVSSKPSEDMTHQISSTRGAANKQSGISEEEVILSQDSFDSSTTQTVDEIRSSSGSNSEADDVTTGFETSKQSDPPVNLIQEKDHSCHDNWSTLIDEPKASIHHLPKEPECSMQLPRMNGVDLNSSSSFIPANSLQQESFVSSGQYQMSATPGPQKAGLLHFGVLGKESTSSLPSSNSEITEACHTSNVTCSENETPKFAGSSQGQYNLPSSHPVHQENFQPEPPVCSSQILNTNHPQVGEFFKETTRHGETLAKGKNGAQKQDTPMFEGIPSLVDKQICFENTVPEAKAKEQNHSSHEPPSGAGTNMSKAQKRKAEDERNRAFDWDSLRKEALSNGEKGERSKDATDSLDYEALRRAHVSEISDAIRERGMNNLLADRIKDFLNRLVRDHGKIDLEWLRDAPPDKAKDYLLSVRGLGLKSVECVRLLTLHHLAFPVDTNVGRIAVRLGWVPLQPLPESLQLHLLEMYPVLESIQKYLWPRLCKLDQLTLYELHYQMITFGKVFCTKSKPNCNACPMRAECRHFASAFASARLALPGPEEKRIVTSDAPVATDPIPPMVIRPMPLPQAENGFDKSERSFGRNCEPIIEEPTTPEPEAAELSISDIEDQYYEDSDEIPSIKLNMEEFTTNLQNIMQDSMELQDDMSKALVALNPNAASIPTPKLKDVSRLRTEHQVYELPDSHRLLEGLDKREPDDPSPYLLAIWTPGETANSVQPPERECSAQQSGKLCDRTTCFSCNNIKEANSQVVRGTILIPCRTAMRGSFPLNGTYFQVNEMFADHASSMNPIDVPRTWIWNLPRRTVYFGTSVSTIFKGLTTGGIQYCFWKGFVCVRGFDRKTRAPRPLMARLHFPASKI

>CcDemethylase-like5

MNFENGPDNRFNKATFGIGTRSFGLEDAVFERNTGGMYDGRFLMNNMWNTIPCSDLLALADAAITTKSEVNMHFQNRNQNQNHEDRQHERTRYLFDLNSPPIATDPQLITGISSQLSPVTPKITRRVDHRQVVSDIINLDEDGSTDEAADKQDNERSDPEQPQLILEQSQDTISMQLEEVNGDFEEELSDDFNPKKTPQPKQRRRKHRPKVVIESKPKSAKKSTPPQPDGSSTGKRKYVRKIMSERSTETPTSEASLPKTRNSCRRKINFEEEQNLHTELQGIKKKTEIELMCDKEFLTDQMTMLSPITPNKSELRHERLAKDVNPMSMRAKRNLSFSRKAHDNDSNCSTSGCFNEDGQETRGSKRECSGDENGLGNRCNNSLEAYLSMITNFPAIYKKKRTEKCQSSVTFRAVSSIWSPEYNNRSFPETSTVYGLNFQQSYKHMLAFGHVESFRKKRSKGVKRMRDLASFAGMIEGKKSASKALCQHHTCMEALSADFSASIATKKRTKRKYIVPSSSNPYDIDALINQFESMSLNNRMVEQDQHALVPYSTRYREKNALVLYKQDKSVVPFEGSYQLRRRKPRPKVDLDDETSRVWRLLLEDINSKGIDGTDEDKEKWWEEERNVFSGRAASFIARMHLVQDSVIGVFLTQNVSDHLSSSAFMNLAARYPLKSKSSNEPLQDDKSSISVKEPCQLDLEETIRWHEGNSNQQPAQDHGSIMLQDADSYEEKEVVNSNERSVRLKDILSGEVLEISRNDSGILESFTQENRGVDDLVSPQNSVDTCPNSVQSSIVDTAERLGSWLVRNSQSEPLDASKPIISEMFTSFASTKLHEVYSQEQAHKPSNKRIGQDKLGSINNSPEQNEGISHDHITSGLRIPPKDHELQTAHGSAALEAEYIELREERIIIDNKPEEKCASEQSEISAESASQATVEMVRTRSFQETPISCNTLQSRTISENSIIVTNQHAHENNCNAQKALDIENCSAEISEVTESNNMTDNSRETAHKIVESNSNEHGNLQTTASGINEGSAEVKKGKTRKAKKIKPNWDSLRIGAQVNHKRETTPNTKDSLDYEAVRCADVNEIADTIKDRGMNNMLAERIKDFLNRLVRDHGSIDLEWLRDVPPDKAKEYLLSFRGLGLKSVECVRLLTLHHLAFPVDTNVGRIAVRLGWVPLQPLPESLQLHLLELYELHYQMITFGKVFCTKNKPNCNACPMRGECRHFASAFASARLALPAPEERSIVSATENKIPDQSPTRITSQLHLTLPLDNHCLHQQSQVQNHDPVIEEPATPEPIVEVPATPEPEKIQQESDIEDFCEDPEEIPMIKLNMEAFTQNLQTYMENNMELAEGDMSRALVALASEAASIPAPKLKNVSQLRTEHQVYELPDSHPLLEGLDKREPDDPCSYLLAIWTPGETAESIQPPGVRCSSEESGTLCHKETCFSCNSIREANSQTIRGTLLVFADHDSSLNPIDVPRSWLWNLPRRTVYFGTSIPTIFKGLTTEGIQYCFWRGFVCVRGFDQKTRAPRPLMARLHFPASKLRRSKGMADET

>CcDemethylase-like1

MVKMVKGRGITLFIVASSDPPAASSSSSFSSPFSRSRSFSLLSISYLCSIVLTGNAIVTSTFLSSSHLDLHQFYQTSPKTLANCSKAKGEMTEPEFGSWRMTPVRSTLIGNGIVGQRDSCTTTHQENGIVVNGEIWNSAMPTKSFPQGNGNGNGTSSWTPVTPGKPVPQRSIPQNQVETENWEDLVEIYQDLLKEETLTLNKVVAQSLYPTPPPSTVGNIGNQHQVASTPNRNLNSTPIPNLNHGSYQPSTSFTYFPSEDPANWDSSSLLAAIVRPKKSSASLNIAQNTSLHASNRTSLPNTSTQVGSNSISVEPDAASSEITGPLAFAPITPDTRQKHTDSQWVLAKDRHESQRNEDGDNHYNEQLHTIGDSTSSAVSTTQKEHLVSEEGDELGIDLNKTPQQKTPARRKKHRPKVIREGKPKKTATPKDPKNTPPNETRVKRKYVRKKDVNVSETPQRNGVEISQNGVPRSSGKRKYVRKKGVENSDVQQKTRVEEATAPVVETPAKSCKKQLNFDLEIVARDGRQDINLNASPRDIEQERRINGILERSAMNVVQNNRYAGVGTHQKISTNHMQAGTQNMALPELLNVPSTPMAKARDVALNVLAQHLTMKNPITVRDVWRNGYNQVGQQQVSPNLEPSGRMVNFDERRGIKRQSFEQMDPHSLNAMDSLIMYQKLLLDRTDASNNLASIILESHKKTKTQSDHLQALVSSTPPLEDNLRGESRQINGVYGNAPASLQLLNSCTGRVEPSYKVMNAGGGNINGRQFQPPRAATQNLQKHMVTSGMQPIAERLQRPTPGHGVNPVTAMISWNRPPATPPKDYSRSALVTYPSPLVDKKRTATSNSSNRRSNGADQVFLQLRKDALEFQQQSFRKPNGGPRKHKVEVLVEDITFKLEGLSIYDGNDKKQNALVPYKGNNAIIPFEPIKKRKPRPKVDLDPETDRLWRLLMGKEGSEATETLDKDKEKWWEDERRVFRGRADSFIARMHLVQGDRRFSRWKGSVVDSVIGVFLTQNVSDHLSSSAFMSLAAKFSPKSTSTNETCCQDGACILVEEPIETVLPNDSTKCHDKIERQPVFNQSSFVSCESSEHMRHHHISSTKAAAIKDNRTSEEVILSQDSLDSSTIQTVDEIRSSSGSNSEAEDQITGFETSKEPGPANPMQAEKVSMFTELFSHDNRSPSLNDRSQYMHHLPKTPPYNMQIPIIGGVNNLNNASRFTPPNSSLHLVQEQLASSSRFQMNMAMGLQNVGSPGFGLLRGGSISSLPSSKSGITEAYHTSNVTYQENEMPRFQAPPLAQYDFLSNHPTHLKSFQPRSHIGSVLNSSHQQGRELYRETTVHAETLAKAQNGAPKQDSCSEDRVSAVDKQNCIENAAAEANSKEQNYASHEPLSGAGTNIPKVRKGTAEDEKKKAFDWDSLRKKVLSNGEKRERSKDAKDSLDYEALRRAHVNEISDAIRERGMNNLLADRIKDFLDRLVRDHESIDLEWLRDVPPDQAKDYLLSIRGLGLKSVECVRLLTLHHLAFPVDTNVGRIAVRLGWVPLQPLPESLQLHLLEMYPVLESIQKYLWPRLCKLDQLTLYELHYQMITFGKVFCTKSKPNCNACPMRAECRHFASAFASARLALPGPEEKRIVTSDAPNGTHTIPPVIMRPMSLPPAENNYSKDAQFSGRECEPIIEEPTTPEPESAELTLSDIEDQYYEDDDEIPTIKLDMNEFTMNLQKMQDSMEGDMSKALVALNPQAASIPTPKLKNVSRLRTEHQVYELPDSHPILKGLDKREPDDPSPYLLAIWTPGETATSTQPPERGCQSQESGMLCDRTTCFSCNCIKEANSQVVRGTILMPCRTAMHGSFPLNGTYFQVNEMFADHASSLNPIAVPRAWIWNLPRRTVYFGTSVSTIFKGINSLDALFVASLSAINTEILVLVGLTTQEIQQCFWRGFVCVRGFDQKTRAPRPLMARLHFPASKLVKTKNEAK

>Sl_DML

METGQGSSWIPATPGKPSFAESPPICSTGQENQQAQVDLSDLQRKQAVEHANGSTAEAQNAVEHANGSTAEAQNAAANRGSTSSVEDQCFTTSEAVVGTKSEMCGGGINMYNNFPSDNVELWSSMSFGDLLAMAHAGGSGTTPADETAYSVKSSFQPLINTQNADESSILSSFPFNLNSPPKMTGATLSSNIQFQFEPVTPDMMKIKGQASNASNLDINVTTAARVIQSNEDIIKGAEANELQQNKEQSVLILEGKLDTELNNTPEQKTRRRKHRPKVVVEDKPKRTPKPKIQKQPGAEETKTEKRKYVRRNKVGEPTATFADEVSNTICHEGKPPSSEKTPTAKRKYVRRNQVNKSTEKPSEEGSSVTIGTPAATSTEEVKNTTFHVGKAPSSEETPTAKRNVRTNQVNMSMEKLSEEGSSGTNDPSEVPHSRKSCRKSLSFELESQASDEYSSYRPSTLDLHANNSGSTAQSVQLGQGKETTSEETEMGITHNITRSLNQEVRNYLSQPRMQYPSPPTPDKVGWNHDKTMVGNHNESTRGNSRIIFSDVTHDKQASILQMTPQSLNSNCGSSSCLPHGKGLKRQHSCRTDEAQFYSINAKGTYFNSMQAYQAILPANKPDVYSNVGMHFPAIYKKMRAEKGHISTSSYIKLFTGETNYVSSSQCYISGSPSNNSATNIGNYGMWNSNVMPAFVEAERLRNKISNGPTQVHDIASLHEIYKQFPTSTSKELTKYGFGERYKTSHLSSACMGTPIADTQAATKKKRQSKKSILVSSAASNLYTHQHVAKNARGSLPALTWRGMSPIDEIAERLRLLDLNRESSQNQGPHGITYHTKFQRESALVLYQRDGSIVPFGSSLVRKRKPRPKVDVDDETDRVWKLLLQDINSEGIDGTDEDKAKWWEEERRVFNSRADSFIARMRLVQGDRRFSPWKGSVVDSVVGVYLTQNVSDHLSSSAFMSLAAHFPLKTDSTQKHEGNTGIIIEEPEECATDPNVSIRWYEDQPNQSTHCQDSSGVYNTDSNEEKPAVNDSESSENSTECIKSAECSVILQSDSSREGSDLYHGSTVTSSQDRKELNDLPSSPSSVVSSEISAVIQASEGTDSSNFCSSTSFLKLLQMAGTSGAQGTRCTEHLQEGENVPFLEKELISPKKSVLSAESAHSALYTTPQNKLDIETMTDAEDNVELQFPTEDSNSNVQQVPEAPACSETIVNVTERASIVFDSCKPEQRGLESSLKNDSNHVRSKVDKVNDNPSKAKNGQLGKEKENIDWDSLRLQAQANGKKRERTANTMDSLDYEAVRCANVNEIAHTIRERGMNNKLAERIQAFLNRIVSDHGSIDLEWLRDVPPDKAKEYLLSIRGLGLKSVECVRLLTLHHLAFPVDVNVGRIAVRLGWVPLQPLPESLQLHLLELYPILESIQQYLWPRLCKLDQRTLYELHYHMITFGKVFCTKSKPNCNACPLRGECRHFASAFASARLALPAPEEKSIVSATEQKATNNNPRENFTHLPLPLPPGNQQPVENQKLINSAPIIEVPATPEPIVEVPSTPEQEQIKAPEIDIEDAYFEDTNEIPMIELNMAEFTQNVKKYVENNMELHQVEMSNALVALTSEAASIPTPKLKNVSRLRTEHQVYELPDSHPLLEGLDKREPDDPSSYLLAIWTPGETANSMQPPETQCNSQESGELCEDETCSSCNSIREAQSQTVRGTLLIPCRTATRGSFPLNGTYFQVNEVFADHDSSLNPINVPRDWLWNLPRRTVYFGTSIPTIFKGLNTESIQHCFWRGFVCVRGFDHKTRAPRPLLARFHFPASKLNRTNGKTNEDKGVAS

>Sl_DML2

MNPGRVFSTPQENGGVQNGDPRIPFSQQKPVLPLPDLVPAEMQRNQIEMTGWPDLLGMYGDFLLMPASETGVVQNSVTSVGWDKGSTGHWSDVVVRNRSSEIDTYSCGNIPDQSKPACTRVNSLEELIGMKNQSNRISTHGRSSNSTRSDIPILRNSYAQVDRRHEQTQLKAAGQTVLNQSQLFKSPNQMVDCYNRHLPLDGMRSPYQVNRSLISPVAQDAGTSPSTNSFFTFAPVTPDHNHFNDNQHFERQNVPIQERSSLEKDGQENVLGSMKSKDNHSDKLLQRVTDSVVVNSPLTEKVDNGNVGNVDIDLNKTPASKTPKRRKHRPKVVIEGETKRTPKRAAPVDGTPNENPSGKRKYVRRDGLKASTTEQTEVNESAAQRNSTPNENPSGKRKYVRRKDPNASTTQQTEVVGKDKVPDAGESEKTCRKMLDFDLEDITKDESLPSTNIHHPEKHQQKKETFDLNLNSQDMELSLAIMEATAISAGQKQRKEEIAEKLLMEKPQELASPLPSANQVTRNNQALNALARSLSMRTVTRYPNSIQLYEPRQLALGRMPLLLRDTAYTDNDGRGSKRDQCPSSPFQPRTFSQMGSVCSEMLGNDNCRRNCSTSSGIPSYTAAAIHDSTKFPSSSLSINRYNRASEEGSRHCASPMVVKHNLQKQTNPSQMHSYAQPIPQHIPQQTAEIHGSQVQATIRNWNHQYQLQSLAMVVQNIERRNSHKKMPAQQNMGKTSPNELSNYVELLPRENKNSRADQHHLTKARGLQETHRHAVSVDTGLLQGLQRHVVSVDTGLQGTHRHAVSVDVITQQLERLFISNSKKNAAQVEQKALVPYKGSGTIIPYEGFDPIKRRKARPRVDLDPETNRLWNVLMGKEESAETMDKDNEKWWEDERKVVRGRVDSFVARMRLVQGDRRFSPWKGSVVDSVIGVFLTQNVSDHLSSSAFMCLAAKFPLPTSTKNTLSQDGCNIVVEEPEVEIIDPDGTTIYHKARLQRRMENHTHTSRAYLVSEHDKRVDEEVISLQNSPDSLILQANEELRSSSGSDLESEDRPSSPNLNKDRTQASHSPPTKWTAAFQEYQSHFMRNGISEKLPVFGNQKIETVADMGRHNENLDAETYLHGYPINPHIQVQEIPIRSASNSWLNMTPEFGKHETACHEKEIDMSKSMKQIAGSSSPLIAQRTTHPFIHAPRMGEIGGVEMQPGKVDNQHSVSSHQNEMAMASQLESSCIRQSVNHSEAVAKGQEEGQAYPSSKQPSITGTSISKTRKRKVEEGDKKAFDWDSLRKEVQSKSGKKERSKDAMDSLNYEAVRSAAVKEISDAIKERGMNNMLAERIKDFLDRLVRDHGSIDLEWLRDVAPDKAKEYLLSIRGLGLKSVECVRLLTLHNLAFPVDTNVGRIAVRLGWVPLQPLPESLQLHLLELYPILESIQKYLWPRLCKLDQRTLYELHYHMITFGKVFCTKSKPNCNACPLRAECRHFASAYASARLALPGPEEKSIVSSAVPIPSEGNAAAAFKPMLLPPAAEVRMAYPYAPIEAGDLPSFLEKSMPIPQEMTDSLNREATVVTNNCQPIIEEPKTPEPLPELLESDIEDGFFEDPDEIPLIELNMKEFTTNLETILQEHNKEGDVSKALVALNPEAASIPTAKLKNVGRLRTEHQVYELPDSHQLLEKWDKREPDDPSPYLLAIWTPGETVNSIQPPETKCDHSGLGNLCNETTCYSCNGIREANTQTVRGTLLIPCRTAMRGSFPLNGTYFQVNEVFADHKSSLDPIHVPRKLLWSLSKRTVYFGTSVSTIFKGLSTEQIQYCFWRGFVCVRGFDREMRAPRPLIARLHFPASKMVKNRSDDKKKEGTAAEKVAGFNSPISVHTK

>Sl_ROS1L1

MYGENNIKTCNDVSTDDIDEWSNVSFGHLLALAHAAGSTAVTENANEEINLALNGSFNSLISSQDADGSSTCSRFPFNLNSPTRMTDEDSSSNNAFPFEPITPYQIKKKGPASDAPGLDINATPIPRHVQSSKDTLKRAEANDLQQNTEKSGLVLNISELSDNMIDKVVDQDAEQNNTPQQKRRKKHRPKVVIEGEHKRTPKPKIPQQHSSMGTKKEKGKYVQRNKIEDPPGTPSDEVNDMTKHEGHLPSSAKIQRARRTYIRRNQVKKFAPKPAEEGSIDPPNVSRPRRYPRRSLNFDSENILSDENSLRWPSSTVEDLHENQSNSSVHPGKGIEASTAKTRLGSVYDLKCSNQELKNCQTHHEMSHTDPFTLKKIGLNHSKFTMNKENGISRGKCKIVFSDETHDKQASILEMTPKSPNSSNCSSSACLIPETPERALKRRRSLRTDQAKLYSTNVRGAYFNSMQAYQAILPATEPYAQSTQGMHFPIIFKKKRTEKGHPSATSYSKPFTCEINYLSLSQSNIGLSQASTSATDNANNLMPNRELVPAFVEAEGLRRKRSKSISKVRDLASLLEICKHFPTSSVKETMVSGFGERYENSDQPNTCMEALVADTRTIMKTKKRSKRSIPVSSTASHMYARSQFPTNARGSIPAITWRSPVDEIAERLQHLNLNRESIHPYQYEENALVIYQRDGSIVPFAGPFVRKRRPRPKVDLDDETTRVWKLLLQDINSEGIDGTDEDKAKWWESEREVFHGRVDSFVARMRLVQGDRRFSPWKGSVVDSVVGVFLTQNVSDHLSSSAFMTLAARFPLKSDISVKKNEERTGIIIEEPEVSTLEPDDTNGWHDYQSTQTTLGQKFFTISSTESDDEKTAVHSSESSENSTNCTSSTENSILQQPGSSRESSCVHHESTTYGSATANAATSFLGDQVEPDDLLSSQNSILSSQDSANFSVVQTSEGTESSNFSGSASFLKLLQIAGTSKSHGVQDQRSENILLEKNINVQLKHVACCSHIQKDGENHRGSIGNDCPCSYLGPCTMSNSGAQQAKFKSDLEEAAKFSDPSGELGDPEQSKSSAEPANQRVAEAPKAPTFSEAIDVREEVSVVVDSSKSEHTVLRSNSNNGKIHAGSTLDGANHNTKAKKEGPGKEKQNVDWDSLRLQAESNGKKREKTANTMDSLDWDAVRCADVNEISHTIRERGMNNMLAERIKDFLNRIFREHGSIDLEWLRDVPPDKAKEYLLSIRGLGLKSVECVRLLTLHNLAFPVDTNVGRIAVRLGWVPLQPLPESLQLHLLELYPVLESIQKYLWPRLCKLDQRTLYELHYHMITFGKVFCSKSKPNCNACPMRGECRHFASAFASARLALPAPEEKSIVSATENNAADQNPFQNFNQQPLTLPQANQTPLEHPKLINSAPIIEVPATPQPIVEEPASPEPEQDAPEIDIEDVCFEDPDEIPTIELNMAQFTQNVKNFVQNNMELQQVEMSKALVALTPAAASIPTPKLKHISRLRTEHQVYELPDSHPLLEGFEKREPDDPSSYLLAIWTPGETSDSIQPPGRQCNSQETGRLCDDETCFACNSIREAHAQTVRGTILIPCRTAMRGSFPLNGTYFQVNEVFADHESSLKPIDVPRNWLWNLPRRTVYFGTSIPSIFKGLTTESIQHCFWRGFVCVRGFDKKLRAPRPLMARLHFPASKLTRTKGKPDEN

>At_DME

MNSRADPGDRYFRVPLENQTQQEFMGSWIPFTPKKPRSSLMVDERVINQDLNGFPGGEFVDRGFCNTGVDHNGVFDHGAHQGVTNLSMMINSLAGSHAQAWSNSERDLLGRSEVTSPLAPVIRNTTGNVEPVNGNFTSDVGMVNGPFTQSGTSQAGYNEFELDDLLNPDQMPFSFTSLLSGGDSLFKVRQYGPPACNKPLYNLNSPIRREAVGSVCESSFQYVPSTPSLFRTGEKTGFLEQIVTTTGHEIPEPKSDKSMQSIMDSSAVNATEATEQNDGSRQDVLEFDLNKTPQQKPSKRKRKFMPKVVVEGKPKRKPRKPAELPKVVVEGKPKRKPRKAATQEKVKSKETGSAKKKNLKESATKKPANVGDMSNKSPEVTLKSCRKALNFDLENPGDARQGDSESEIVQNSSGANSFSEIRDAIGGTNGSFLDSVSQIDKTNGLGAMNQPLEVSMGNQPDKLSTGAKLARDQQPDLLTRNQQCQFPVATQNTQFPMENQQAWLQMKNQLIGFPFGNQQPRMTIRNQQPCLAMGNQQPMYLIGTPRPALVSGNQQLGGPQGNKRPIFLNHQTCLPAGNQLYGSPTDMHQLVMSTGGQQHGLLIKNQQPGSLIRGQQPCVPLIDQQPATPKGFTHLNQMVATSMSSPGLRPHSQSQVPTTYLHVESVSRILNGTTGTCQRSRAPAYDSLQQDIHQGNKYILSHEISNGNGCKKALPQNSSLPTPIMAKLEEARGSKRQYHRAMGQTEKHDLNLAQQIAQSQDVERHNSSTCVEYLDAAKKTKIQKVVQENLHGMPPEVIEIEDDPTDGARKGKNTASISKGASKGNSSPVKKTAEKEKCIVPKTPAKKGRAGRKKSVPPPAHASEIQLWQPTPPKTPLSRSKPKGKGRKSIQDSGKARGPSGELLCQDSIAEIIYRMQNLYLGDKEREQEQNAMVLYKGDGALVPYESKKRKPRPKVDIDDETTRIWNLLMGKGDEKEGDEEKDKKKEKWWEEERRVFRGRADSFIARMHLVQGDRRFSPWKGSVVDSVIGVFLTQNVSDHLSSSAFMSLAARFPPKLSSSREDERNVRSVVVEDPEGCILNLNEIPSWQEKVQHPSDMEVSGVDSGSKEQLRDCSNSGIERFNFLEKSIQNLEEEVLSSQDSFDPAIFQSCGRVGSCSCSKSDAEFPTTRCETKTVSGTSQSVQTGSPNLSDEICLQGNERPHLYEGSGDVQKQETTNVAQKKPDLEKTMNWKDSVCFGQPRNDTNWQTTPSSSYEQCATRQPHVLDIEDFGMQGEGLGYSWMSISPRVDRVKNKNVPRRFFRQGGSVPREFTGQIIPSTPHELPGMGLSGSSSAVQEHQDDTQHNQQDEMNKASHLQKTFLDLLNSSEECLTRQSSTKQNITDGCLPRDRTAEDVVDPLSNNSSLQNILVESNSSNKEQTAVEYKETNATILREMKGTLADGKKPTSQWDSLRKDVEGNEGRQERNKNNMDSIDYEAIRRASISEISEAIKERGMNNMLAVRIKDFLERIVKDHGGIDLEWLRESPPDKAKDYLLSIRGLGLKSVECVRLLTLHNLAFPVDTNVGRIAVRMGWVPLQPLPESLQLHLLELYPVLESIQKFLWPRLCKLDQRTLYELHYQLITFGKVFCTKSRPNCNACPMRGECRHFASAYASARLALPAPEERSLTSATIPVPPESYPPVAIPMIELPLPLEKSLASGAPSNRENCEPIIEEPASPGQECTEITESDIEDAYYNEDPDEIPTIKLNIEQFGMTLREHMERNMELQEGDMSKALVALHPTTTSIPTPKLKNISRLRTEHQVYELPDSHRLLDGMDKREPDDPSPYLLAIWTPGETANSAQPPEQKCGGKASGKMCFDETCSECNSLREANSQTVRGTLLIPCRTAMRGSFPLNGTYFQVNELFADHESSLKPIDVPRDWIWDLPRRTVYFGTSVTSIFRGLSTEQIQFCFWKGFVCVRGFEQKTRAPRPLMARLHFPASKLKNNKT

>At_DML2

MEVEGEVREKEARVKGRQPETEVLHGLPQEQSIFNNMQHNHQPDSDRRRLSLENLPGLYNMSCTQLLALANATVATGSSIGASSSSLSSQHPTDSWINSWKMDSNPWTLSKMQKQQYDVSTPQKFLCDLNLTPEELVSTSTQRTEPESPQITLKTPGKSLSETDHEPHDRIKKSVLGTGSPAAVKKRKIARNDEKSQLETPTLKRKKIRPKVVREGKTKKASSKAGIKKSSIAATATKTSEESNYVRPKRLTRRSIRFDFDLQEEDEEFCGIDFTSAGHVEGSSGEENLTDTTLGMFGHVPKGRRGQRRSNGFKKTDNDCLSSMLSLVNTGPGSFMESEEDRPSDSQISLGRQRSIMATRPRNFRSLKKLLQRIIPSKRDRKGCKLPRGLPKLTVASKLQLKVFRKKRSQRNRVASQFNARILDLQWRRQNPTGTSLADIWERSLTIDAITKLFEELDINKEGLCLPHNRETALILYKKSYEEQKAIVKYSKKQKPKVQLDPETSRVWKLLMSSIDCDGVDGSDEEKRKWWEEERNMFHGRANSFIARMRVVQGNRTFSPWKGSVVDSVVGVFLTQNVADHSSSSAYMDLAAEFPVEWNFNKGSCHEEWGSSVTQETILNLDPRTGVSTPRIRNPTRVIIEEIDDDENDIDAVCSQESSKTSDSSITSADQSKTMLLDPFNTVLMNEQVDSQMVKGKGHIPYTDDLNDLSQGISMVSSASTHCELNLNEVPPEVELCSHQQDPESTIQTQDQQESTRTEDVKKNRKKPTTSKPKKKSKESAKSTQKKSVDWDSLRKEAESGGRKRERTERTMDTVDWDALRCTDVHKIANIIIKRGMNNMLAERIKAFLNRLVKKHGSIDLEWLRDVPPDKAKEYLLSINGLGLKSVECVRLLSLHQIAFPVDTNVGRIAVRLGWVPLQPLPDELQMHLLELYPVLESVQKYLWPRLCKLDQKTLYELHYHMITFGKVFCTKVKPNCNACPMKAECRHYSSARASARLALPEPEESDRTSVMIHERRSKRKPVVVNFRPSLFLYQEKEQEAQRSQNCEPIIEEPASPEPEYIEHDIEDYPRDKNNVGTSEDPWENKDVIPTIILNKEAGTSHDLVVNKEAGTSHDLVVLSTYAAAIPRRKLKIKEKLRTEHHVFELPDHHSILEGFERREAEDIVPYLLAIWTPGETVNSIQPPKQRCALFESNNTLCNENKCFQCNKTREEESQTVRGTILIPCRTAMRGGFPLNGTYFQTNEVFADHDSSINPIDVPTELIWDLKRRVAYLGSSVSSICKGLSVEAIKYNFQEGYVCVRGFDRENRKPKSLVKRLHCSHVAIRTKEKTEE

>At_DML3

MLTDGSQHTYQNGETKNSKEHERKCDESAHLQDNSQTTHKKKEKKNSKEKHGIKHSESEHLQDDISQRVTGKGRRRNSKGTPKKLRFNRPRILEDGKKPRNPATTRLRTISNKRRKKDIDSEDEVIPELATPTKESFPKRRKNEKIKRSVARTLNFKQEIVLSCLEFDKICGPIFPRGKKRTTTRRRYDFLCFLLPMPVWKKQSRRSKRRKNMVRWARIASSSKLLEETLPLIVSHPTINGQADASLHIDDTLVRHVVSKQTKKSANNVIEHLNRQITYQKDHGLSSLADVPLHIEDTLIKSASSVLSERPIKKTKDIAKLIKDMGRLKINKKVTTMIKADKKLVTAKVNLDPETIKEWDVLMVNDSPSRSYDDKETEAKWKKEREIFQTRIDLFINRMHRLQGNRKFKQWKGSVVDSVVGVFLTQNTTDYLSSNAFMSVAAKFPVDAREGLSYYIEEPQDAKSSECIILSDESISKVEDHENTAKRKNEKTGIIEDEIVDWNNLRRMYTKEGSRPEMHMDSVNWSDVRLSGQNVLETTIKKRGQFRILSERILKFLNDEVNQNGNIDLEWLRNAPSHLVKRYLLEIEGIGLKSAECVRLLGLKHHAFPVDTNVGRIAVRLGLVPLEPLPNGVQMHQLFEYPSMDSIQKYLWPRLCKLPQETLYELHYQMITFGKVFCTKTIPNCNACPMKSECKYFASAYVSSKVLLESPEEKMHEPNTFMNAHSQDVAVDMTSNINLVEECVSSGCSDQAICYKPLVEFPSSPRAEIPESTDIEDVPFMNLYQSYASVPKIDFDLDALKKSVEDALVISGRMSSSDEEISKALVIPTPENACIPIKPPRKMKYYNRLRTEHVVYVLPDNHELLHDFERRKLDDPSPYLLAIWQPGETSSSFVPPKKKCSSDGSKLCKIKNCSYCWTIREQNSNIFRGTILIPCRTAMRGAFPLNGTYFQTNEVFADHETSLNPIVFRRELCKGLEKRALYCGSTVTSIFKLLDTRRIELCFWTGFLCLRAFDRKQRDPKELVRRLHTPPDERGPKFMSDDDI

>At_ROS1

MEKQRREESSFQQPPWIPQTPMKPFSPICPYTVEDQYHSSQLEERRFVGNKDMSGLDHLSFGDLLALANTASLIFSGQTPIPTRNTEVMQKGTEEVESLSSVSNNVAEQILKTPEKPKRKKHRPKVRREAKPKREPKPRAPRKSVVTDGQESKTPKRKYVRKKVEVSKDQDATPVESSAAVETSTRPKRLCRRVLDFEAENGENQTNGDIREAGEMESALQEKQLDSGNQELKDCLLSAPSTPKRKRSQGKRKGVQPKKNGSNLEEVDISMAQAAKRRQGPTCCDMNLSGIQYDEQCDYQKMHWLYSPNLQQGGMRYDAICSKVFSGQQHNYVSAFHATCYSSTSQLSANRVLTVEERREGIFQGRQESELNVLSDKIDTPIKKKTTGHARFRNLSSMNKLVEVPEHLTSGYCSKPQQNNKILVDTRVTVSKKKPTKSEKSQTKQKNLLPNLCRFPPSFTGLSPDELWKRRNSIETISELLRLLDINREHSETALVPYTMNSQIVLFGGGAGAIVPVTPVKKPRPRPKVDLDDETDRVWKLLLENINSEGVDGSDEQKAKWWEEERNVFRGRADSFIARMHLVQGDRRFTPWKGSVVDSVVGVFLTQNVSDHLSSSAFMSLASQFPVPFVPSSNFDAGTSSMPSIQITYLDSEETMSSPPDHNHSSVTLKNTQPDEEKDYVPSNETSRSSSEIAISAHESVDKTTDSKEYVDSDRKGSSVEVDKTDEKCRVLNLFPSEDSALTCQHSMVSDAPQNTERAGSSSEIDLEGEYRTSFMKLLQGVQVSLEDSNQVSPNMSPGDCSSEIKGFQSMKEPTKSSVDSSEPGCCSQQDGDVLSCQKPTLKEKGKKVLKEEKKAFDWDCLRREAQARAGIREKTRSTMDTVDWKAIRAADVKEVAETIKSRGMNHKLAERIQGFLDRLVNDHGSIDLEWLRDVPPDKAKEYLLSFNGLGLKSVECVRLLTLHHLAFPVDTNVGRIAVRLGWVPLQPLPESLQLHLLEMYPMLESIQKYLWPRLCKLDQKTLYELHYQMITFGKVFCTKSKPNCNACPMKGECRHFASAFASARLALPSTEKGMGTPDKNPLPLHLPEPFQREQGSEVVQHSEPAKKVTCCEPIIEEPASPEPETAEVSIADIEEAFFEDPEEIPTIRLNMDAFTSNLKKIMEHNKELQDGNMSSALVALTAETASLPMPKLKNISQLRTEHRVYELPDEHPLLAQLEKREPDDPCSYLLAIWTPGETADSIQPSVSTCIFQANGMLCDEETCFSCNSIKETRSQIVRGTILIPCRTAMRGSFPLNGTYFQVNEVFADHASSLNPINVPRELIWELPRRTVYFGTSVPTIFKGLSTEKIQACFWKGYVCVRGFDRKTRGPKPLIARLHFPASKLKGQQANLA
