# Supplementary material for: The genome-wide identification and transcriptional levels of DNA methyltransferases and demethylases in globe artichoke
Source: PLoS One. 2017 Jul 26;12(7):e0181669. doi: 10.1371/journal.pone.0181669 (PMC5529103; doi:10.1371/journal.pone.0181669)
Supplement: S2 File — A. thaliana and S. lycopersicum sequences have also been used for searching for globe artichoke homologs. (DOCX) [file pone.0181669.s007.docx]

**S2 File**

>CcDemethylase-like3

MDLKSEFGYFESFYNGFENQKHHQVDLDSAGYVAAADAAHRLMESLTSSNFSSNDCISISIDFHERLEPIVGLIREEVSPMKLSNDDEDKLVGEKQAMTKMQQVKKFRPKVMTPKPITPSPSSSSSRGHTSVKSSCRRALDFDAQTKTMLIYTKGGHCFTRSVNKYMEGRLECNDKEEDMNEQEEELFRQRALSFITSMRHVQGNRGFMGWKGSVVDSVVGVFLTQNAPDNLSSSAFMCLAAKYLIEDPKEGISKHALDWNAVRCAQPYEISHVIQERGMNNRIAARIQVTFLDSIYNHKSGLLDDLEWLRKAKPEKTMEFFSKIYGLGIKSMDCLRLLTLRQHAFPVDRHVARIVVRLGWVPVEKLPDGVLIHELEEYPMMEAVQDYLSQRLSNLDVDTLYELHYQMITFGKVFCTKKKPNCNSCPLKKECKHFASAFGRFPPQGEFKTFVPRTPTPGQSSSRVQVILEEDIEDLCKVHPVIKVMKNAAGKGKEEEEVVVGRFLVPCRTATRGSFPLDGTFFQINEVFADDESCKKPVVVSRNLLSDLTIKTLFCGTSISAIFQVAAVSIDLVPLVTASHELRLCRLPRVAASHELRLCRLPRVAASQSAVSISPPSYYSFLLLLVPPSASFRLLPVGRSSLDNS

>CcDemethylase-like4

MGEEGESSSSRQVYPPEVAYAPATPAKPDRSDWGPIGIDWLKNQFDEVIFEETSAKKSISCWEGNSVSTSHIYDLSGFSMDDVETWNSISCRDLLALADATIRRGSDDGDHDRSDGLDFDNRSNCIDTQQYGWLNLGNYSPDLNLPPEMVMKPLVSTGLSTQITPGTPDQARRAEHKQMGSDIVAKVVADNNKERYNLDEQPQVQVLVEQLQGDVSTIVEANQDFEKGLTAETNLNETPQPKQRRRKHRPKVVREGQQKKAKQSATPQKPDGSSTGKRKYVRKKGVEKSPATPAVEEGSGTIDPSSDQQNKKSCRKKINFDETEKGNEVTVETAEVNITVDKTCSMNQVVETILESQSASPITPSKTELPIKDAKHTYRKVKCRINFLQETHDKRPSSVSSPNESNCSTSASFNKGEAQGSKMELSSKIVGMELWDENAIGVGCNLSKFTNDCSGGKQGMHLPANKKKRIEKCRSSITSGAISSVCSAQSSDCSFLAEQNASKAPQMSKDYVLKDDQQPYKQAFGHLENSKKKRRSKALSLIPDLALFPGIVEGRHWQTPKEGSRYEVAYQQQTYTEAHAADFHVSIATKKRMKKNAKLPSLYQDHLRFTKGCIDSLINQFERLDINSQMAEEGRDALIPYLSRYNEKNALVLYQERGLVPFEGLFNPVKRRKPRPKVDLDEETSRVWTLLLENINSQGIDGTDEDKAKWWEEERRVFRGRADSFIARMHLVQGDRRFSRWKGSVLDSVIGVFLTQNVSDHLSSSAFMALAARYPLKSKSSSEPLHDDESILSVKEPCQVDQDETITWHEKLNQPSGDHGPMMLQDIDLCEEKEVVNSNKFPKNSGCVDLNVSSEGEVPELAEKDLAMYKESVVDQIENDDIASSQNSANMSPSSVQSSVAHTTERLGSCSKEEQKDMSKATIFGGYTSFVELLHMQGTTTVHETYSQQQAEESSNKKIGQDELESVAYLEEQNDGISPHSNSSRALEVETFELREERNITQKKSQEEFASEESGLSAESASQAMVQLVKTTSSQEASKSCNTCHIRLQERSRSRCKMIAVNPNINTEQHTEDNNCEVQEVIAIANVSADNSKATESNNILKASGETAHKVVEINSVDHGTHQIVNGMDEGSSKVKRQKSGKVKQKIEWDNLRLHAEVTEKRERTPNTSDSLDYEAVRTADVNEVADTIKERGMNNVLAARIKDLLDRLVEDHGSIDLEWLRDVPPDKAKEYLLSFRGLGLKSVECVRLLTLHHLAFPVDTNVGRIAVRLGWVPLQPLPESLQLHLLELYPVLESIQKYLWPRLCKLDQRTLYELHYQMITFGKVFCTKSKPNCNACPMRGECRHFASAFASARLGLPAPEERMASLTENRTGQSSIGLIEQCHITLPSASEQWQQLSDIQNCNSGIEEPATPGSTVEVPATSGPIVEVPATSGTIVEVPATSGPIVEVPATPGPIVEVPETPGPIIEVPATPEPEPIQEEFDIEDFCEDSEEIPMIKLNIEEFTQNLQTYMEKHMVLGEGDMSKALVALTSEAASIPTPKLKNVSQLRTEHQVYELPDSHPLLEGLDTREPDDPCSYLLAIWTPGETADSIQPPEGQCCSQESGTLCSEETCFFCNSTREANSQTVRGTLLIPCRTAMRGSFPLNGTYFQVNEVFADHESSLNPIDVPRSWLWNLPRRTVYFGTSIPTIFKGLTTEDIQYCFWRDVDAVSVVNPRYKWQDNHVQTAAADIQILQKTLSSWPEWHDS

>CcDemethylase-like2

MNFERGFPIPRHGNEDSRSSVTHGIPVPPESGIEHHHGSWTATSGGSGTVGGGDARATPRRPIPMDNGTVHGSESWNTINLEQPTPMGNGVTEDRNPTLERTILQRSGIARNANDWKSVIPGNLFPHRNGIGQSRNSWSPATPEKIVNQRSNTENGLESENWQDLIGMYTGLLKEDTVDKNGVLEDINPTPSKVRDYGNQNWVASNNKNATHRSSPSPYWNHTSNQASTTSDPYLKTNDPANWNSNLLATLVGSQNSSTHTSSANKAQTNGIHHISNRSAVPNSANQVESNSMRSTSWTSMLGSQRTMRFTSNNLINDAHNTEDGFPVAYQPGYKPNSPPRSAASSIIDSFPFAPITPDNQGKHMHSQRVPENGNFRVEGTSTPAKDSRENQTVSREDAENLYNELLQTIGDSPPSAISTTQKELGVPENTDEQGIDLNKTPQQKTPTRRKKHRPKVIREGKPKRTPKPKDPSDGTSNETRVKRKYVRKKGINILETQGDDVTKNIPVASVGKRKYVRKKGLEKFGDEQQSRMDDVATSVVGIPAKSCKKQLNFDLDPVAQDESYGIRSSQQGIDVNVETPARSCKKQLKFDLEPVAQDKGYGIRSSQEGININVNPQDIGQERRINSILERPAIEIAQQNISMQSGNQYELNVPITPLANTKHHALNMLARNMTIKNSIPEHDRRGNLYNKVNQRFHGEGIENLVLQADMVSTNLERVREPNLMSTPQSLASKGMLNLDERRGIKRQSPEQMCLNANTMDSLLLYQKLLLGVAHRAYDRNNLSSILLENSKKTKMQSEFQTLVSSEPSCIIPHKPRQETRQINGIYGNGSAMHLLTSSTEGVNPYKTMHVGGNVINGQFRPPMAATHYLQKHQVFSGMQHHPLRSVPERSQRYIQGHDIGSKTAIMSWNLPPPTPSKETSRYAVTAYPATSLEKRQTAKPNSYNQRLNGLNQMFQHHRNDPLKGYQQPTTVARGRPRKQKPELSVDDITYRLEGLHIYDGNKKEQHELVLYRGSNALIPFEPIKKRIPRPKVDLDPETDRLWRLLMGKEGSEGAETLENGKEKWWEEERRVFRGRADSFIARMHLVQGDRRFSRWKGSVVDSVIGVFLTQNVSDHLSSSAFMSLAAKFPPKLSTTKETCCQDGACEEPIEVAEPNGITKCHEKIKQPVPDQSFFVSSKPSEDMTHQISSTRGAANKQSGISEEEVILSQDSFDSSTTQTVDEIRSSSGSNSEADDVTTGFETSKQSDPPVNLIQEKDHSCHDNWSTLIDEPKASIHHLPKEPECSMQLPRMNGVDLNSSSSFIPANSLQQESFVSSGQYQMSATPGPQKAGLLHFGVLGKESTSSLPSSNSEITEACHTSNVTCSENETPKFAGSSQGQYNLPSSHPVHQENFQPEPPVCSSQILNTNHPQVGEFFKETTRHGETLAKGKNGAQKQDTPMFEGIPSLVDKQICFENTVPEAKAKEQNHSSHEPPSGAGTNMSKAQKRKAEDERNRAFDWDSLRKEALSNGEKGERSKDATDSLDYEALRRAHVSEISDAIRERGMNNLLADRIKDFLNRLVRDHGKIDLEWLRDAPPDKAKDYLLSVRGLGLKSVECVRLLTLHHLAFPVDTNVGRIAVRLGWVPLQPLPESLQLHLLEMYPVLESIQKYLWPRLCKLDQLTLYELHYQMITFGKVFCTKSKPNCNACPMRAECRHFASAFASARLALPGPEEKRIVTSDAPVATDPIPPMVIRPMPLPQAENGFDKSERSFGRNCEPIIEEPTTPEPEAAELSISDIEDQYYEDSDEIPSIKLNMEEFTTNLQNIMQDSMELQDDMSKALVALNPNAASIPTPKLKDVSRLRTEHQVYELPDSHRLLEGLDKREPDDPSPYLLAIWTPGETANSVQPPERECSAQQSGKLCDRTTCFSCNNIKEANSQVVRGTILIPCRTAMRGSFPLNGTYFQVNEMFADHASSMNPIDVPRTWIWNLPRRTVYFGTSVSTIFKGLTTGGIQYCFWKGFVCVRGFDRKTRAPRPLMARLHFPASKI

>CcDemethylase-like5

MNFENGPDNRFNKATFGIGTRSFGLEDAVFERNTGGMYDGRFLMNNMWNTIPCSDLLALADAAITTKSEVNMHFQNRNQNQNHEDRQHERTRYLFDLNSPPIATDPQLITGISSQLSPVTPKITRRVDHRQVVSDIINLDEDGSTDEAADKQDNERSDPEQPQLILEQSQDTISMQLEEVNGDFEEELSDDFNPKKTPQPKQRRRKHRPKVVIESKPKSAKKSTPPQPDGSSTGKRKYVRKIMSERSTETPTSEASLPKTRNSCRRKINFEEEQNLHTELQGIKKKTEIELMCDKEFLTDQMTMLSPITPNKSELRHERLAKDVNPMSMRAKRNLSFSRKAHDNDSNCSTSGCFNEDGQETRGSKRECSGDENGLGNRCNNSLEAYLSMITNFPAIYKKKRTEKCQSSVTFRAVSSIWSPEYNNRSFPETSTVYGLNFQQSYKHMLAFGHVESFRKKRSKGVKRMRDLASFAGMIEGKKSASKALCQHHTCMEALSADFSASIATKKRTKRKYIVPSSSNPYDIDALINQFESMSLNNRMVEQDQHALVPYSTRYREKNALVLYKQDKSVVPFEGSYQLRRRKPRPKVDLDDETSRVWRLLLEDINSKGIDGTDEDKEKWWEEERNVFSGRAASFIARMHLVQDSVIGVFLTQNVSDHLSSSAFMNLAARYPLKSKSSNEPLQDDKSSISVKEPCQLDLEETIRWHEGNSNQQPAQDHGSIMLQDADSYEEKEVVNSNERSVRLKDILSGEVLEISRNDSGILESFTQENRGVDDLVSPQNSVDTCPNSVQSSIVDTAERLGSWLVRNSQSEPLDASKPIISEMFTSFASTKLHEVYSQEQAHKPSNKRIGQDKLGSINNSPEQNEGISHDHITSGLRIPPKDHELQTAHGSAALEAEYIELREERIIIDNKPEEKCASEQSEISAESASQATVEMVRTRSFQETPISCNTLQSRTISENSIIVTNQHAHENNCNAQKALDIENCSAEISEVTESNNMTDNSRETAHKIVESNSNEHGNLQTTASGINEGSAEVKKGKTRKAKKIKPNWDSLRIGAQVNHKRETTPNTKDSLDYEAVRCADVNEIADTIKDRGMNNMLAERIKDFLNRLVRDHGSIDLEWLRDVPPDKAKEYLLSFRGLGLKSVECVRLLTLHHLAFPVDTNVGRIAVRLGWVPLQPLPESLQLHLLELYELHYQMITFGKVFCTKNKPNCNACPMRGECRHFASAFASARLALPAPEERSIVSATENKIPDQSPTRITSQLHLTLPLDNHCLHQQSQVQNHDPVIEEPATPEPIVEVPATPEPEKIQQESDIEDFCEDPEEIPMIKLNMEAFTQNLQTYMENNMELAEGDMSRALVALASEAASIPAPKLKNVSQLRTEHQVYELPDSHPLLEGLDKREPDDPCSYLLAIWTPGETAESIQPPGVRCSSEESGTLCHKETCFSCNSIREANSQTIRGTLLVFADHDSSLNPIDVPRSWLWNLPRRTVYFGTSIPTIFKGLTTEGIQYCFWRGFVCVRGFDQKTRAPRPLMARLHFPASKLRRSKGMADET

>CcDemethylase-like1

MVKMVKGRGITLFIVASSDPPAASSSSSFSSPFSRSRSFSLLSISYLCSIVLTGNAIVTSTFLSSSHLDLHQFYQTSPKTLANCSKAKGEMTEPEFGSWRMTPVRSTLIGNGIVGQRDSCTTTHQENGIVVNGEIWNSAMPTKSFPQGNGNGNGTSSWTPVTPGKPVPQRSIPQNQVETENWEDLVEIYQDLLKEETLTLNKVVAQSLYPTPPPSTVGNIGNQHQVASTPNRNLNSTPIPNLNHGSYQPSTSFTYFPSEDPANWDSSSLLAAIVRPKKSSASLNIAQNTSLHASNRTSLPNTSTQVGSNSISVEPDAASSEITGPLAFAPITPDTRQKHTDSQWVLAKDRHESQRNEDGDNHYNEQLHTIGDSTSSAVSTTQKEHLVSEEGDELGIDLNKTPQQKTPARRKKHRPKVIREGKPKKTATPKDPKNTPPNETRVKRKYVRKKDVNVSETPQRNGVEISQNGVPRSSGKRKYVRKKGVENSDVQQKTRVEEATAPVVETPAKSCKKQLNFDLEIVARDGRQDINLNASPRDIEQERRINGILERSAMNVVQNNRYAGVGTHQKISTNHMQAGTQNMALPELLNVPSTPMAKARDVALNVLAQHLTMKNPITVRDVWRNGYNQVGQQQVSPNLEPSGRMVNFDERRGIKRQSFEQMDPHSLNAMDSLIMYQKLLLDRTDASNNLASIILESHKKTKTQSDHLQALVSSTPPLEDNLRGESRQINGVYGNAPASLQLLNSCTGRVEPSYKVMNAGGGNINGRQFQPPRAATQNLQKHMVTSGMQPIAERLQRPTPGHGVNPVTAMISWNRPPATPPKDYSRSALVTYPSPLVDKKRTATSNSSNRRSNGADQVFLQLRKDALEFQQQSFRKPNGGPRKHKVEVLVEDITFKLEGLSIYDGNDKKQNALVPYKGNNAIIPFEPIKKRKPRPKVDLDPETDRLWRLLMGKEGSEATETLDKDKEKWWEDERRVFRGRADSFIARMHLVQGDRRFSRWKGSVVDSVIGVFLTQNVSDHLSSSAFMSLAAKFSPKSTSTNETCCQDGACILVEEPIETVLPNDSTKCHDKIERQPVFNQSSFVSCESSEHMRHHHISSTKAAAIKDNRTSEEVILSQDSLDSSTIQTVDEIRSSSGSNSEAEDQITGFETSKEPGPANPMQAEKVSMFTELFSHDNRSPSLNDRSQYMHHLPKTPPYNMQIPIIGGVNNLNNASRFTPPNSSLHLVQEQLASSSRFQMNMAMGLQNVGSPGFGLLRGGSISSLPSSKSGITEAYHTSNVTYQENEMPRFQAPPLAQYDFLSNHPTHLKSFQPRSHIGSVLNSSHQQGRELYRETTVHAETLAKAQNGAPKQDSCSEDRVSAVDKQNCIENAAAEANSKEQNYASHEPLSGAGTNIPKVRKGTAEDEKKKAFDWDSLRKKVLSNGEKRERSKDAKDSLDYEALRRAHVNEISDAIRERGMNNLLADRIKDFLDRLVRDHESIDLEWLRDVPPDQAKDYLLSIRGLGLKSVECVRLLTLHHLAFPVDTNVGRIAVRLGWVPLQPLPESLQLHLLEMYPVLESIQKYLWPRLCKLDQLTLYELHYQMITFGKVFCTKSKPNCNACPMRAECRHFASAFASARLALPGPEEKRIVTSDAPNGTHTIPPVIMRPMSLPPAENNYSKDAQFSGRECEPIIEEPTTPEPESAELTLSDIEDQYYEDDDEIPTIKLDMNEFTMNLQKMQDSMEGDMSKALVALNPQAASIPTPKLKNVSRLRTEHQVYELPDSHPILKGLDKREPDDPSPYLLAIWTPGETATSTQPPERGCQSQESGMLCDRTTCFSCNCIKEANSQVVRGTILMPCRTAMHGSFPLNGTYFQVNEMFADHASSLNPIAVPRAWIWNLPRRTVYFGTSVSTIFKGINSLDALFVASLSAINTEILVLVGLTTQEIQQCFWRGFVCVRGFDQKTRAPRPLMARLHFPASKLVKTKNEAK

>Sl_DML

METGQGSSWIPATPGKPSFAESPPICSTGQENQQAQVDLSDLQRKQAVEHANGSTAEAQNAVEHANGSTAEAQNAAANRGSTSSVEDQCFTTSEAVVGTKSEMCGGGINMYNNFPSDNVELWSSMSFGDLLAMAHAGGSGTTPADETAYSVKSSFQPLINTQNADESSILSSFPFNLNSPPKMTGATLSSNIQFQFEPVTPDMMKIKGQASNASNLDINVTTAARVIQSNEDIIKGAEANELQQNKEQSVLILEGKLDTELNNTPEQKTRRRKHRPKVVVEDKPKRTPKPKIQKQPGAEETKTEKRKYVRRNKVGEPTATFADEVSNTICHEGKPPSSEKTPTAKRKYVRRNQVNKSTEKPSEEGSSVTIGTPAATSTEEVKNTTFHVGKAPSSEETPTAKRNVRTNQVNMSMEKLSEEGSSGTNDPSEVPHSRKSCRKSLSFELESQASDEYSSYRPSTLDLHANNSGSTAQSVQLGQGKETTSEETEMGITHNITRSLNQEVRNYLSQPRMQYPSPPTPDKVGWNHDKTMVGNHNESTRGNSRIIFSDVTHDKQASILQMTPQSLNSNCGSSSCLPHGKGLKRQHSCRTDEAQFYSINAKGTYFNSMQAYQAILPANKPDVYSNVGMHFPAIYKKMRAEKGHISTSSYIKLFTGETNYVSSSQCYISGSPSNNSATNIGNYGMWNSNVMPAFVEAERLRNKISNGPTQVHDIASLHEIYKQFPTSTSKELTKYGFGERYKTSHLSSACMGTPIADTQAATKKKRQSKKSILVSSAASNLYTHQHVAKNARGSLPALTWRGMSPIDEIAERLRLLDLNRESSQNQGPHGITYHTKFQRESALVLYQRDGSIVPFGSSLVRKRKPRPKVDVDDETDRVWKLLLQDINSEGIDGTDEDKAKWWEEERRVFNSRADSFIARMRLVQGDRRFSPWKGSVVDSVVGVYLTQNVSDHLSSSAFMSLAAHFPLKTDSTQKHEGNTGIIIEEPEECATDPNVSIRWYEDQPNQSTHCQDSSGVYNTDSNEEKPAVNDSESSENSTECIKSAECSVILQSDSSREGSDLYHGSTVTSSQDRKELNDLPSSPSSVVSSEISAVIQASEGTDSSNFCSSTSFLKLLQMAGTSGAQGTRCTEHLQEGENVPFLEKELISPKKSVLSAESAHSALYTTPQNKLDIETMTDAEDNVELQFPTEDSNSNVQQVPEAPACSETIVNVTERASIVFDSCKPEQRGLESSLKNDSNHVRSKVDKVNDNPSKAKNGQLGKEKENIDWDSLRLQAQANGKKRERTANTMDSLDYEAVRCANVNEIAHTIRERGMNNKLAERIQAFLNRIVSDHGSIDLEWLRDVPPDKAKEYLLSIRGLGLKSVECVRLLTLHHLAFPVDVNVGRIAVRLGWVPLQPLPESLQLHLLELYPILESIQQYLWPRLCKLDQRTLYELHYHMITFGKVFCTKSKPNCNACPLRGECRHFASAFASARLALPAPEEKSIVSATEQKATNNNPRENFTHLPLPLPPGNQQPVENQKLINSAPIIEVPATPEPIVEVPSTPEQEQIKAPEIDIEDAYFEDTNEIPMIELNMAEFTQNVKKYVENNMELHQVEMSNALVALTSEAASIPTPKLKNVSRLRTEHQVYELPDSHPLLEGLDKREPDDPSSYLLAIWTPGETANSMQPPETQCNSQESGELCEDETCSSCNSIREAQSQTVRGTLLIPCRTATRGSFPLNGTYFQVNEVFADHDSSLNPINVPRDWLWNLPRRTVYFGTSIPTIFKGLNTESIQHCFWRGFVCVRGFDHKTRAPRPLLARFHFPASKLNRTNGKTNEDKGVAS

>Sl_DML2

MNPGRVFSTPQENGGVQNGDPRIPFSQQKPVLPLPDLVPAEMQRNQIEMTGWPDLLGMYGDFLLMPASETGVVQNSVTSVGWDKGSTGHWSDVVVRNRSSEIDTYSCGNIPDQSKPACTRVNSLEELIGMKNQSNRISTHGRSSNSTRSDIPILRNSYAQVDRRHEQTQLKAAGQTVLNQSQLFKSPNQMVDCYNRHLPLDGMRSPYQVNRSLISPVAQDAGTSPSTNSFFTFAPVTPDHNHFNDNQHFERQNVPIQERSSLEKDGQENVLGSMKSKDNHSDKLLQRVTDSVVVNSPLTEKVDNGNVGNVDIDLNKTPASKTPKRRKHRPKVVIEGETKRTPKRAAPVDGTPNENPSGKRKYVRRDGLKASTTEQTEVNESAAQRNSTPNENPSGKRKYVRRKDPNASTTQQTEVVGKDKVPDAGESEKTCRKMLDFDLEDITKDESLPSTNIHHPEKHQQKKETFDLNLNSQDMELSLAIMEATAISAGQKQRKEEIAEKLLMEKPQELASPLPSANQVTRNNQALNALARSLSMRTVTRYPNSIQLYEPRQLALGRMPLLLRDTAYTDNDGRGSKRDQCPSSPFQPRTFSQMGSVCSEMLGNDNCRRNCSTSSGIPSYTAAAIHDSTKFPSSSLSINRYNRASEEGSRHCASPMVVKHNLQKQTNPSQMHSYAQPIPQHIPQQTAEIHGSQVQATIRNWNHQYQLQSLAMVVQNIERRNSHKKMPAQQNMGKTSPNELSNYVELLPRENKNSRADQHHLTKARGLQETHRHAVSVDTGLLQGLQRHVVSVDTGLQGTHRHAVSVDVITQQLERLFISNSKKNAAQVEQKALVPYKGSGTIIPYEGFDPIKRRKARPRVDLDPETNRLWNVLMGKEESAETMDKDNEKWWEDERKVVRGRVDSFVARMRLVQGDRRFSPWKGSVVDSVIGVFLTQNVSDHLSSSAFMCLAAKFPLPTSTKNTLSQDGCNIVVEEPEVEIIDPDGTTIYHKARLQRRMENHTHTSRAYLVSEHDKRVDEEVISLQNSPDSLILQANEELRSSSGSDLESEDRPSSPNLNKDRTQASHSPPTKWTAAFQEYQSHFMRNGISEKLPVFGNQKIETVADMGRHNENLDAETYLHGYPINPHIQVQEIPIRSASNSWLNMTPEFGKHETACHEKEIDMSKSMKQIAGSSSPLIAQRTTHPFIHAPRMGEIGGVEMQPGKVDNQHSVSSHQNEMAMASQLESSCIRQSVNHSEAVAKGQEEGQAYPSSKQPSITGTSISKTRKRKVEEGDKKAFDWDSLRKEVQSKSGKKERSKDAMDSLNYEAVRSAAVKEISDAIKERGMNNMLAERIKDFLDRLVRDHGSIDLEWLRDVAPDKAKEYLLSIRGLGLKSVECVRLLTLHNLAFPVDTNVGRIAVRLGWVPLQPLPESLQLHLLELYPILESIQKYLWPRLCKLDQRTLYELHYHMITFGKVFCTKSKPNCNACPLRAECRHFASAYASARLALPGPEEKSIVSSAVPIPSEGNAAAAFKPMLLPPAAEVRMAYPYAPIEAGDLPSFLEKSMPIPQEMTDSLNREATVVTNNCQPIIEEPKTPEPLPELLESDIEDGFFEDPDEIPLIELNMKEFTTNLETILQEHNKEGDVSKALVALNPEAASIPTAKLKNVGRLRTEHQVYELPDSHQLLEKWDKREPDDPSPYLLAIWTPGETVNSIQPPETKCDHSGLGNLCNETTCYSCNGIREANTQTVRGTLLIPCRTAMRGSFPLNGTYFQVNEVFADHKSSLDPIHVPRKLLWSLSKRTVYFGTSVSTIFKGLSTEQIQYCFWRGFVCVRGFDREMRAPRPLIARLHFPASKMVKNRSDDKKKEGTAAEKVAGFNSPISVHTK

>Sl_ROS1L1

MYGENNIKTCNDVSTDDIDEWSNVSFGHLLALAHAAGSTAVTENANEEINLALNGSFNSLISSQDADGSSTCSRFPFNLNSPTRMTDEDSSSNNAFPFEPITPYQIKKKGPASDAPGLDINATPIPRHVQSSKDTLKRAEANDLQQNTEKSGLVLNISELSDNMIDKVVDQDAEQNNTPQQKRRKKHRPKVVIEGEHKRTPKPKIPQQHSSMGTKKEKGKYVQRNKIEDPPGTPSDEVNDMTKHEGHLPSSAKIQRARRTYIRRNQVKKFAPKPAEEGSIDPPNVSRPRRYPRRSLNFDSENILSDENSLRWPSSTVEDLHENQSNSSVHPGKGIEASTAKTRLGSVYDLKCSNQELKNCQTHHEMSHTDPFTLKKIGLNHSKFTMNKENGISRGKCKIVFSDETHDKQASILEMTPKSPNSSNCSSSACLIPETPERALKRRRSLRTDQAKLYSTNVRGAYFNSMQAYQAILPATEPYAQSTQGMHFPIIFKKKRTEKGHPSATSYSKPFTCEINYLSLSQSNIGLSQASTSATDNANNLMPNRELVPAFVEAEGLRRKRSKSISKVRDLASLLEICKHFPTSSVKETMVSGFGERYENSDQPNTCMEALVADTRTIMKTKKRSKRSIPVSSTASHMYARSQFPTNARGSIPAITWRSPVDEIAERLQHLNLNRESIHPYQYEENALVIYQRDGSIVPFAGPFVRKRRPRPKVDLDDETTRVWKLLLQDINSEGIDGTDEDKAKWWESEREVFHGRVDSFVARMRLVQGDRRFSPWKGSVVDSVVGVFLTQNVSDHLSSSAFMTLAARFPLKSDISVKKNEERTGIIIEEPEVSTLEPDDTNGWHDYQSTQTTLGQKFFTISSTESDDEKTAVHSSESSENSTNCTSSTENSILQQPGSSRESSCVHHESTTYGSATANAATSFLGDQVEPDDLLSSQNSILSSQDSANFSVVQTSEGTESSNFSGSASFLKLLQIAGTSKSHGVQDQRSENILLEKNINVQLKHVACCSHIQKDGENHRGSIGNDCPCSYLGPCTMSNSGAQQAKFKSDLEEAAKFSDPSGELGDPEQSKSSAEPANQRVAEAPKAPTFSEAIDVREEVSVVVDSSKSEHTVLRSNSNNGKIHAGSTLDGANHNTKAKKEGPGKEKQNVDWDSLRLQAESNGKKREKTANTMDSLDWDAVRCADVNEISHTIRERGMNNMLAERIKDFLNRIFREHGSIDLEWLRDVPPDKAKEYLLSIRGLGLKSVECVRLLTLHNLAFPVDTNVGRIAVRLGWVPLQPLPESLQLHLLELYPVLESIQKYLWPRLCKLDQRTLYELHYHMITFGKVFCSKSKPNCNACPMRGECRHFASAFASARLALPAPEEKSIVSATENNAADQNPFQNFNQQPLTLPQANQTPLEHPKLINSAPIIEVPATPQPIVEEPASPEPEQDAPEIDIEDVCFEDPDEIPTIELNMAQFTQNVKNFVQNNMELQQVEMSKALVALTPAAASIPTPKLKHISRLRTEHQVYELPDSHPLLEGFEKREPDDPSSYLLAIWTPGETSDSIQPPGRQCNSQETGRLCDDETCFACNSIREAHAQTVRGTILIPCRTAMRGSFPLNGTYFQVNEVFADHESSLKPIDVPRNWLWNLPRRTVYFGTSIPSIFKGLTTESIQHCFWRGFVCVRGFDKKLRAPRPLMARLHFPASKLTRTKGKPDEN

>At_DME

MNSRADPGDRYFRVPLENQTQQEFMGSWIPFTPKKPRSSLMVDERVINQDLNGFPGGEFVDRGFCNTGVDHNGVFDHGAHQGVTNLSMMINSLAGSHAQAWSNSERDLLGRSEVTSPLAPVIRNTTGNVEPVNGNFTSDVGMVNGPFTQSGTSQAGYNEFELDDLLNPDQMPFSFTSLLSGGDSLFKVRQYGPPACNKPLYNLNSPIRREAVGSVCESSFQYVPSTPSLFRTGEKTGFLEQIVTTTGHEIPEPKSDKSMQSIMDSSAVNATEATEQNDGSRQDVLEFDLNKTPQQKPSKRKRKFMPKVVVEGKPKRKPRKPAELPKVVVEGKPKRKPRKAATQEKVKSKETGSAKKKNLKESATKKPANVGDMSNKSPEVTLKSCRKALNFDLENPGDARQGDSESEIVQNSSGANSFSEIRDAIGGTNGSFLDSVSQIDKTNGLGAMNQPLEVSMGNQPDKLSTGAKLARDQQPDLLTRNQQCQFPVATQNTQFPMENQQAWLQMKNQLIGFPFGNQQPRMTIRNQQPCLAMGNQQPMYLIGTPRPALVSGNQQLGGPQGNKRPIFLNHQTCLPAGNQLYGSPTDMHQLVMSTGGQQHGLLIKNQQPGSLIRGQQPCVPLIDQQPATPKGFTHLNQMVATSMSSPGLRPHSQSQVPTTYLHVESVSRILNGTTGTCQRSRAPAYDSLQQDIHQGNKYILSHEISNGNGCKKALPQNSSLPTPIMAKLEEARGSKRQYHRAMGQTEKHDLNLAQQIAQSQDVERHNSSTCVEYLDAAKKTKIQKVVQENLHGMPPEVIEIEDDPTDGARKGKNTASISKGASKGNSSPVKKTAEKEKCIVPKTPAKKGRAGRKKSVPPPAHASEIQLWQPTPPKTPLSRSKPKGKGRKSIQDSGKARGPSGELLCQDSIAEIIYRMQNLYLGDKEREQEQNAMVLYKGDGALVPYESKKRKPRPKVDIDDETTRIWNLLMGKGDEKEGDEEKDKKKEKWWEEERRVFRGRADSFIARMHLVQGDRRFSPWKGSVVDSVIGVFLTQNVSDHLSSSAFMSLAARFPPKLSSSREDERNVRSVVVEDPEGCILNLNEIPSWQEKVQHPSDMEVSGVDSGSKEQLRDCSNSGIERFNFLEKSIQNLEEEVLSSQDSFDPAIFQSCGRVGSCSCSKSDAEFPTTRCETKTVSGTSQSVQTGSPNLSDEICLQGNERPHLYEGSGDVQKQETTNVAQKKPDLEKTMNWKDSVCFGQPRNDTNWQTTPSSSYEQCATRQPHVLDIEDFGMQGEGLGYSWMSISPRVDRVKNKNVPRRFFRQGGSVPREFTGQIIPSTPHELPGMGLSGSSSAVQEHQDDTQHNQQDEMNKASHLQKTFLDLLNSSEECLTRQSSTKQNITDGCLPRDRTAEDVVDPLSNNSSLQNILVESNSSNKEQTAVEYKETNATILREMKGTLADGKKPTSQWDSLRKDVEGNEGRQERNKNNMDSIDYEAIRRASISEISEAIKERGMNNMLAVRIKDFLERIVKDHGGIDLEWLRESPPDKAKDYLLSIRGLGLKSVECVRLLTLHNLAFPVDTNVGRIAVRMGWVPLQPLPESLQLHLLELYPVLESIQKFLWPRLCKLDQRTLYELHYQLITFGKVFCTKSRPNCNACPMRGECRHFASAYASARLALPAPEERSLTSATIPVPPESYPPVAIPMIELPLPLEKSLASGAPSNRENCEPIIEEPASPGQECTEITESDIEDAYYNEDPDEIPTIKLNIEQFGMTLREHMERNMELQEGDMSKALVALHPTTTSIPTPKLKNISRLRTEHQVYELPDSHRLLDGMDKREPDDPSPYLLAIWTPGETANSAQPPEQKCGGKASGKMCFDETCSECNSLREANSQTVRGTLLIPCRTAMRGSFPLNGTYFQVNELFADHESSLKPIDVPRDWIWDLPRRTVYFGTSVTSIFRGLSTEQIQFCFWKGFVCVRGFEQKTRAPRPLMARLHFPASKLKNNKT

>At_DML2

MEVEGEVREKEARVKGRQPETEVLHGLPQEQSIFNNMQHNHQPDSDRRRLSLENLPGLYNMSCTQLLALANATVATGSSIGASSSSLSSQHPTDSWINSWKMDSNPWTLSKMQKQQYDVSTPQKFLCDLNLTPEELVSTSTQRTEPESPQITLKTPGKSLSETDHEPHDRIKKSVLGTGSPAAVKKRKIARNDEKSQLETPTLKRKKIRPKVVREGKTKKASSKAGIKKSSIAATATKTSEESNYVRPKRLTRRSIRFDFDLQEEDEEFCGIDFTSAGHVEGSSGEENLTDTTLGMFGHVPKGRRGQRRSNGFKKTDNDCLSSMLSLVNTGPGSFMESEEDRPSDSQISLGRQRSIMATRPRNFRSLKKLLQRIIPSKRDRKGCKLPRGLPKLTVASKLQLKVFRKKRSQRNRVASQFNARILDLQWRRQNPTGTSLADIWERSLTIDAITKLFEELDINKEGLCLPHNRETALILYKKSYEEQKAIVKYSKKQKPKVQLDPETSRVWKLLMSSIDCDGVDGSDEEKRKWWEEERNMFHGRANSFIARMRVVQGNRTFSPWKGSVVDSVVGVFLTQNVADHSSSSAYMDLAAEFPVEWNFNKGSCHEEWGSSVTQETILNLDPRTGVSTPRIRNPTRVIIEEIDDDENDIDAVCSQESSKTSDSSITSADQSKTMLLDPFNTVLMNEQVDSQMVKGKGHIPYTDDLNDLSQGISMVSSASTHCELNLNEVPPEVELCSHQQDPESTIQTQDQQESTRTEDVKKNRKKPTTSKPKKKSKESAKSTQKKSVDWDSLRKEAESGGRKRERTERTMDTVDWDALRCTDVHKIANIIIKRGMNNMLAERIKAFLNRLVKKHGSIDLEWLRDVPPDKAKEYLLSINGLGLKSVECVRLLSLHQIAFPVDTNVGRIAVRLGWVPLQPLPDELQMHLLELYPVLESVQKYLWPRLCKLDQKTLYELHYHMITFGKVFCTKVKPNCNACPMKAECRHYSSARASARLALPEPEESDRTSVMIHERRSKRKPVVVNFRPSLFLYQEKEQEAQRSQNCEPIIEEPASPEPEYIEHDIEDYPRDKNNVGTSEDPWENKDVIPTIILNKEAGTSHDLVVNKEAGTSHDLVVLSTYAAAIPRRKLKIKEKLRTEHHVFELPDHHSILEGFERREAEDIVPYLLAIWTPGETVNSIQPPKQRCALFESNNTLCNENKCFQCNKTREEESQTVRGTILIPCRTAMRGGFPLNGTYFQTNEVFADHDSSINPIDVPTELIWDLKRRVAYLGSSVSSICKGLSVEAIKYNFQEGYVCVRGFDRENRKPKSLVKRLHCSHVAIRTKEKTEE

>At_DML3

MLTDGSQHTYQNGETKNSKEHERKCDESAHLQDNSQTTHKKKEKKNSKEKHGIKHSESEHLQDDISQRVTGKGRRRNSKGTPKKLRFNRPRILEDGKKPRNPATTRLRTISNKRRKKDIDSEDEVIPELATPTKESFPKRRKNEKIKRSVARTLNFKQEIVLSCLEFDKICGPIFPRGKKRTTTRRRYDFLCFLLPMPVWKKQSRRSKRRKNMVRWARIASSSKLLEETLPLIVSHPTINGQADASLHIDDTLVRHVVSKQTKKSANNVIEHLNRQITYQKDHGLSSLADVPLHIEDTLIKSASSVLSERPIKKTKDIAKLIKDMGRLKINKKVTTMIKADKKLVTAKVNLDPETIKEWDVLMVNDSPSRSYDDKETEAKWKKEREIFQTRIDLFINRMHRLQGNRKFKQWKGSVVDSVVGVFLTQNTTDYLSSNAFMSVAAKFPVDAREGLSYYIEEPQDAKSSECIILSDESISKVEDHENTAKRKNEKTGIIEDEIVDWNNLRRMYTKEGSRPEMHMDSVNWSDVRLSGQNVLETTIKKRGQFRILSERILKFLNDEVNQNGNIDLEWLRNAPSHLVKRYLLEIEGIGLKSAECVRLLGLKHHAFPVDTNVGRIAVRLGLVPLEPLPNGVQMHQLFEYPSMDSIQKYLWPRLCKLPQETLYELHYQMITFGKVFCTKTIPNCNACPMKSECKYFASAYVSSKVLLESPEEKMHEPNTFMNAHSQDVAVDMTSNINLVEECVSSGCSDQAICYKPLVEFPSSPRAEIPESTDIEDVPFMNLYQSYASVPKIDFDLDALKKSVEDALVISGRMSSSDEEISKALVIPTPENACIPIKPPRKMKYYNRLRTEHVVYVLPDNHELLHDFERRKLDDPSPYLLAIWQPGETSSSFVPPKKKCSSDGSKLCKIKNCSYCWTIREQNSNIFRGTILIPCRTAMRGAFPLNGTYFQTNEVFADHETSLNPIVFRRELCKGLEKRALYCGSTVTSIFKLLDTRRIELCFWTGFLCLRAFDRKQRDPKELVRRLHTPPDERGPKFMSDDDI

>At_ROS1

MEKQRREESSFQQPPWIPQTPMKPFSPICPYTVEDQYHSSQLEERRFVGNKDMSGLDHLSFGDLLALANTASLIFSGQTPIPTRNTEVMQKGTEEVESLSSVSNNVAEQILKTPEKPKRKKHRPKVRREAKPKREPKPRAPRKSVVTDGQESKTPKRKYVRKKVEVSKDQDATPVESSAAVETSTRPKRLCRRVLDFEAENGENQTNGDIREAGEMESALQEKQLDSGNQELKDCLLSAPSTPKRKRSQGKRKGVQPKKNGSNLEEVDISMAQAAKRRQGPTCCDMNLSGIQYDEQCDYQKMHWLYSPNLQQGGMRYDAICSKVFSGQQHNYVSAFHATCYSSTSQLSANRVLTVEERREGIFQGRQESELNVLSDKIDTPIKKKTTGHARFRNLSSMNKLVEVPEHLTSGYCSKPQQNNKILVDTRVTVSKKKPTKSEKSQTKQKNLLPNLCRFPPSFTGLSPDELWKRRNSIETISELLRLLDINREHSETALVPYTMNSQIVLFGGGAGAIVPVTPVKKPRPRPKVDLDDETDRVWKLLLENINSEGVDGSDEQKAKWWEEERNVFRGRADSFIARMHLVQGDRRFTPWKGSVVDSVVGVFLTQNVSDHLSSSAFMSLASQFPVPFVPSSNFDAGTSSMPSIQITYLDSEETMSSPPDHNHSSVTLKNTQPDEEKDYVPSNETSRSSSEIAISAHESVDKTTDSKEYVDSDRKGSSVEVDKTDEKCRVLNLFPSEDSALTCQHSMVSDAPQNTERAGSSSEIDLEGEYRTSFMKLLQGVQVSLEDSNQVSPNMSPGDCSSEIKGFQSMKEPTKSSVDSSEPGCCSQQDGDVLSCQKPTLKEKGKKVLKEEKKAFDWDCLRREAQARAGIREKTRSTMDTVDWKAIRAADVKEVAETIKSRGMNHKLAERIQGFLDRLVNDHGSIDLEWLRDVPPDKAKEYLLSFNGLGLKSVECVRLLTLHHLAFPVDTNVGRIAVRLGWVPLQPLPESLQLHLLEMYPMLESIQKYLWPRLCKLDQKTLYELHYQMITFGKVFCTKSKPNCNACPMKGECRHFASAFASARLALPSTEKGMGTPDKNPLPLHLPEPFQREQGSEVVQHSEPAKKVTCCEPIIEEPASPEPETAEVSIADIEEAFFEDPEEIPTIRLNMDAFTSNLKKIMEHNKELQDGNMSSALVALTAETASLPMPKLKNISQLRTEHRVYELPDEHPLLAQLEKREPDDPCSYLLAIWTPGETADSIQPSVSTCIFQANGMLCDEETCFSCNSIKETRSQIVRGTILIPCRTAMRGSFPLNGTYFQVNEVFADHASSLNPINVPRELIWELPRRTVYFGTSVPTIFKGLSTEKIQACFWKGYVCVRGFDRKTRGPKPLIARLHFPASKLKGQQANLA
